# Chromosome 11q13 amplification correlates with poor response and prognosis to PD-1 blockade in unresectable hepatocellular carcinoma

**DOI:** 10.3389/fimmu.2023.1116057

**Published:** 2023-03-28

**Authors:** Kai Yan, Ding Zhang, Yanan Chen, Wenfeng Lu, Mengli Huang, Jinping Cai, Shiqing Chen, Ting Bei, Yuezong Bai, Jian Lv, Yong Fu, Haibin Zhang

**Affiliations:** ^1^ Department of Hepatic Surgery (V), The Third Affiliated Hospital of Naval Medical University, Shanghai, China; ^2^ The Medical Department, 3D Medicines Inc., Shanghai, China; ^3^ Department of Thoracic Surgery, Changzheng Hospital, Shanghai, China

**Keywords:** hyperprogressive disease, hepatocellular carcinoma, programmed cell death protein-1, chromosome 11q13 amplification, next-generation sequencing, multiplex immunofluorescence

## Abstract

**Background & aims:**

Little is known about molecular biomarkers that predict the response and prognosis in unresectable hepatocellular carcinoma (HCC) treated with programmed death (PD)-1 inhibitors.

**Methods:**

A total of 62 HCC patients who underwent next-generation sequencing were retrospectively included in our department for this study. Patients with unresectable disease were subjected to systemic therapy. PD-1 inhibitors intervention (PD-1Ab) group and nonPD-1Ab group included 20 and 13 patients, respectively. Primary resistance was defined as initial on-treatment progression or progression with an initial stable disease of less than 6 months.

**Results:**

Chromosome 11q13 amplification (Amp11q13) was the most common copy number variation in our cohort. Fifteen (24.2%) patients harbored Amp11q13 in our dataset. Patients with Amp11q13 showed higher level of Des-γ-carboxy-prothrombin (DCP), tumor number and were more prone to be combined with portal vein tumor thrombosis (PVTT). In the PD-1Ab group, the proportion of progressive disease (PD) in patients with Amp11q13 was significantly higher than that in patients with nonAmp11q13 (100% vs 33.3%, *P*=0.03). In the nonPD-1Ab group, the proportion of PD in patients with Amp11q13 and nonAmp11q13 had no significant difference (0% vs 11.1%, *P*>0.99). In the PD-1Ab group, the median progression-free survival (PFS) was 1.5 months in Amp11q13 patients vs 16.2 months in non-Amp11q13 patients (HR, 0.05; 95% CI 0.01-0.45; *P* = 0.0003). No significant difference was observed in the nonPD-1Ab group. Notably, we found that hyperprogressive disease (HPD) might be associated with Amp11q13. The increased density of Foxp3+ Treg cells in HCC patients with Amp11q13 might be one of potential mechanisms.

**Conclusion:**

HCC patients with Amp11q13 are less likely to benefit from PD-1 blockade therapies. These findings may help guide the use of immunotherapy for HCC in routine clinical practice.

## Introduction

Hepatocellular carcinoma (HCC) is one of the most common malignancies with a poor prognosis, which ranks as the fourth leading cause of cancer-related mortality worldwide (1). Chronic alcohol consumption, diabetes, non-alcoholic steatohepatitis related to obesity, and hepatitis virus infections are among the primary risk factors associated with the development of HCC (2). Overall, the prognosis of HCC is generally poor, with a five-year survival rate of less than 20% (3). For patients with early-stage HCC, hepatic resection and transplantation have become the mainstay curative treatments (4) However, most patients with HCC are diagnosed with advanced-stage or unresectable diseases (5). Recently, systemic therapies, including immune checkpoint inhibitors (ICIs), tyrosine kinase inhibitors (TKIs), and vascular endothelial growth factor/vascular endothelial growth factor receptor (VEGF/VEGFR) monoclonal antibodies, have challenged the treatment landscape of unresectable HCC (uHCC) with remarkable survival benefits. Specifically, ICIs represented by programmed cell death protein 1 (PD-1)/programmed cell death protein ligand 1 (PD-L1) blockade were developed to reinvigorate exhausted T cells in the tumor microenvironment (6). The objective response rate (ORR) of PD-1 antibody monotherapy was reported to be in the range of 18-20% (7, 8). The combination of ICIs with TKIs or VEGF antibodies further improved the treatment efficacy (9, 10). For instance, the combinational usage of atezolizumab (a PD-L1 inhibitor) and bevacizumab has become the standard of care with a median overall survival (OS) of 19.2 months (11).

Compared to other treatment approaches, immunotherapies have demonstrated the ability to generate responses that are rapid and durable in some patients (12). Currently, there is no reliable biomarker for predicting the efficacy of immunotherapy for HCC, despite the widespread use of PD-L1 expression as a marker in several tumor types (13). In HCC, there is no consistent conclusion regarding the predictive value of PD-L1 expression. CheckMate-459, KEYNOTE-224, and IMbrave150 studies showed higher response rates for PD-L1 positive advanced HCC patients treated with immunotherapy, while the CheckMate-040 study showed no significant difference between PD-L1 positive and negative patients treated with nivolumab (11, 14). The potential predictive value of tumor mutational burden (TMB), microsatellite instability (MSI), and gut microbiota in the immunotherapy of HCC needs further investigation (15, 16).

Although PD-1 monotherapy has significantly improved the landscape of hepatocellular carcinoma treatment, a large proportion of patients do not respond to treatment, or develop progression after a variable period of benefit. Resistance to ICIs stems from both cancer cell intrinsic and extrinsic factors, including impaired antigen presentation, overexpression of inhibitory immune checkpoints, abrogation of the interferon-gamma signaling pathway, and recruitment of immunosuppressive cells (17). Somatic genetic factors also play vital roles in the response of ICIs. In patients with non-cutaneous melanoma, genetic aberrations in the cyclin-dependent kinase 4 (CDK4) pathway were shown to be associated with innate resistance to PD-1 blockade (18). Cyclin D1 (CCND1) amplification was also associated with a poor prognosis in patients receiving ICIs, even those with a high TMB (19). For patients with HCC, genetic aberrations such as Wnt/β-Catenin mutations and lower immune scores may contribute to the resistance in immunotherapy (20, 21). Nevertheless, the molecular profiles of HCC patients with the resistance to PD-1 blockade have not been fully characterized.

Hyperprogressive disease (HPD), a special form of primary resistance characterized by paradoxically accelerated progression after immunotherapy, has been highlighted by several publications (22, 23). It is assessed by calculating the change in tumor growth dynamics. Depending on the criteria used to define HPD, the incidence of HPD ranges from 4% to 29% (24). Due to the rapid deterioration of clinical status, HPD patients often have limited opportunities to receive other therapies. Several biomarkers have been proposed to predict HPD following immunotherapy, but large-scale validation is needed. However, some debates that HPD may reflect merely the natural course of disease in a subset of patients (25).

In this study, based on genetic profiling, we sought to identify genetic biomarkers to predict the response to PD-1 blockade. Our data suggested that the amplification of the chromosome 11q13 locus, including CCND1, fibroblast growth factor 3 (FGF3), FGF4 and FGF19, might predict poor response and prognosis to PD-1 blockade in uHCC.

## Patients and methods

### Patients and samples

**Figure 1:**
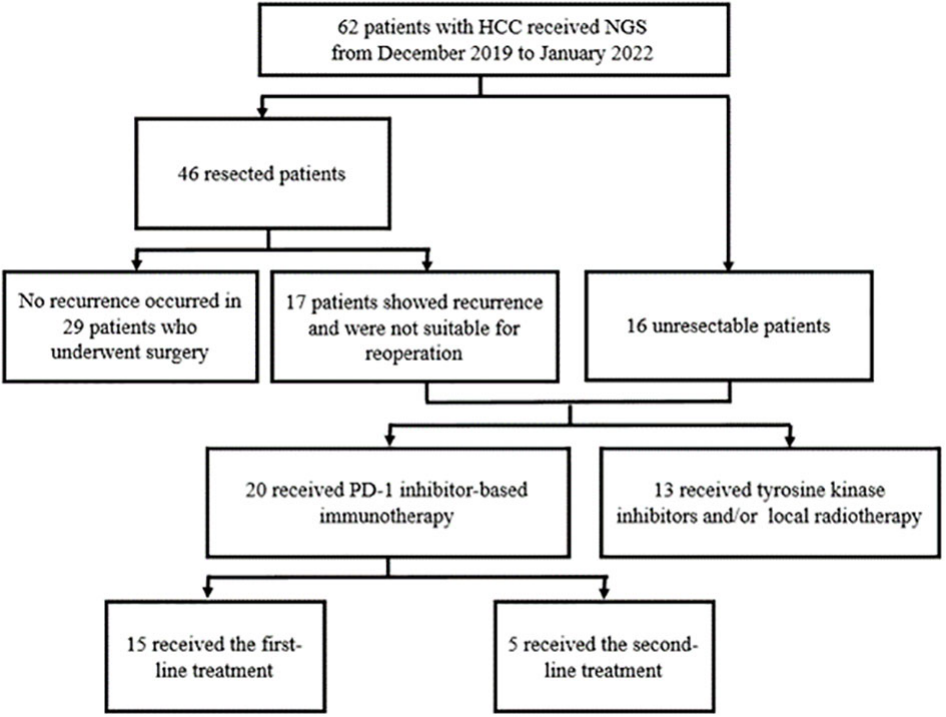
Patient flow diagram.

To explore the correlation between genetic phenotype and response to PD-1 inhibitors, we reviewed the NGS data from patients with uHCC who underwent systemic therapy in our center. The process of case inclusion is shown in **Figure 1**. A total of 62 patients underwent genetic testing from January 2019 to March 2022, of which 46 patients received surgical resection and 16 patients were diagnosed with uHCC. In clinical practice, the indications of NGS testing for patients who underwent surgery are tumors with high risk factors for recurrence, such as tumor size >5 cm, positive MVI, multiple tumors, or tumors with portal vein tumor thrombosis (PVTT). Of the patients who underwent surgical resection, 17 developed recurrence and were diagnosed with uHCC. Therefore, a total of 33 patients with uHCC (17 recurred and 16 uHCC) with available sequencing data were retrospectively reviewed.

According to the NCCN guidelines (Version 5.2022), uHCC is defined as HCC with insufficient hepatic functional reserve (Child-Pugh B or C); insufficient residual liver volume (<40% for patients with Child Pugh grade A cirrhosis); complicated tumor location which was not suitable for surgical resection; or tumors invading large blood vessels or with distant metastases.

For genetic sequencing, next-generation sequencing (NGS) was performed on formalin-fixed paraffin-embedded (FFPE) tumor specimens or blood samples by 3D Medicines Inc. (Shanghai, China), which is certified and accredited by Clinical Laboratory Improvement Amendments (CLIA) and College of American Pathologist (CAP). The panel covered 733 cancer-related genes. Specifically, for patients who developed recurrence, NGS was performed on the resected specimen, which was collected during the initial operation; for patients with uHCC, NGS was performed on tissue or blood samples.

The treatment strategy for each patient with uHCC was determined by a multidisciplinary team. The options included locoregional therapies, TKIs, anti-VEGF antibodies, and PD-1 inhibitors. According to whether or not they had received PD-1 inhibitors, patients were divided into two groups. Namely, those who received PD-1 inhibitors as first- or second-line therapies were classified into the PD-1Ab group, and the others into the nonPD-1Ab group, respectively. The response to treatments was measured regularly, and the treatment strategy was replaced upon progressive disease (PD). All procedures were conducted in accordance with the Helsinki Declaration and with approval from the Ethics Committee of the Third Affiliated Hospital of Naval Medical University (ID: ChiECRCT20220048). Written informed consent was obtained from all participants. Clinicopathological information was extracted from the hospital information management system (HIMS). Prognostic information was obtained by telephone calls. All patients were followed for >6 months. Primary resistance was defined as initial on-treatment progression or progression with an initial stable disease of less than 6 months.

### DNA extractions, library preparation, targeted capture, targeted sequencing and data processing

The assay methodology of DNA extraction and sequencing followed the methods published in a previous paper with some modifications (26). Namely, tumor genomic DNA in the FFPE specimens and cell-free DNA (cfDNA) in the plasma were extracted using QIAamp DNA FFPE Tissue kit (Qiagen GmbH, Hilden, Germany), and normal genomic DNA was extracted from peripheral blood mononuclear cells using QIAamp DNA Blood Mini kit (Qiagen GmbH, Hilden, Germany), respectively, following the manufacturer’s protocols. Libraries were prepared with the KAPA Hyper Prep Kit (KAPA Biosystems, Japan) following the guidelines of the manufacturer. Barcoded with unique molecular identifiers (UMIs) for cfDNA library preparation were individually adopted. For targeted capture, indexed libraries were subjected to probe-based hybridization with a customized NGS panel targeting 733 cancer-related genes. NGS sequencing was performed on the NovaSeq 6000 platform (Illumina, USA) for 100 bp paired-end sequencing with an average coverage depth of 2000× for tumor specimens and 35000× for cfDNA.

Raw data of paired samples were mapped to the reference human genome hg19 using the Burrows-Wheeler Aligner (v0.7.12) (27). PCR duplicate reads were removed and sequence metrics were collected using Picard (v1.130) and SAMtools (v1.1.19) for tissue-based testing, and an in-house developed software was used to generate duplex consensus sequences based on dual UMI integrated at the end of the DNA fragments for ctDNA-based testing. Variant calling was performed only in the targeted regions. Somatic single nucleotide variants (SNVs) were detected using an in-house developed R package to execute a variant detection model based on a binomial test. Local realignment was performed to detect indels. Variants were then filtered by their unique supporting read depth, strand bias, and base quality as previously described (28). Single-nucleotide polymorphism (SNPs) and indels were annotated by ANNOVAR against the following databases: dbSNP (v138), 1000Genome and ESP6500 (population frequency > 0.015). Only missense, stopgain, frameshift and non-frameshift indel mutations were kept. Copy number variations (CNVs) and gene rearrangements were detected as described previously (28).

### Efficacy evaluation

Patients receiving locoregional therapies (LRTs) or systemic therapies were monitored for response evaluation every 6 weeks. If any symptoms or signs suggesting a progressive disease were observed at any time, one extra assessment was performed. Tumor size measurement using radiologic imaging was conducted by radiologists from the Third Affiliated Hospital of Naval Medical University. Assessment of objective response was confirmed by clinicians per Response Evaluation Criteria in Solid Tumors (RECIST) version 1.1 and the date of disease progression was documented (29). The definition of primary resistance was initial on-treatment progression or progression with an initial stable disease of less than 6 months (30). Progression-free survival (PFS) was defined as the time from the onset of treatment to disease progression or death by any cause.

### Assessment of tumor growth dynamics and definition of HPD

Tumor growth dynamics were evaluated based on tumor growth rate (TGR) and tumor growth kinetics (TGK), as described before (31, 32). Briefly, TGR was calculated as the log-scale calibrated change in the sum of the volumes of the target lesions per month according to RECIST 1.1 criteria (33). TGK was calculated as the change in the sum of the longest diameters of the target lesions according to RECIST 1.1 criteria per month (32). Changes in the tumour growth dynamics were assessed by calculating fold changes in TGK and TGR (34).

The defined criteria for HPD in our study were as follows: (1) time from the beginning therapy to treatment failure (TTF) < 2 months; (2) at least 50% increase in tumor burden for the first-line treatment or 2-fold increases in both TGK and tumor TGR ratios for the second-line treatment.

### Multiplex immunofluorescence

Thirty FFPE samples were subjected to assessment of mIHC using the Akoya OPAL Polaris 7-Color Automation IHC kit (NEL871001KT). FFPE tissue samples were deparaffinized in a BOND RX system (Leica Biosystems) and then incubated sequentially with primary antibodies targeting CD68 (Abcam, ab213363, 1:1000), PD-L1 (CST, E1L3N, 13684S, 1:400), PD-1 (CST, D4W2J, 86163S, 1:200), CD163 (Abcam, ab182422, 1:500), CD3 (Dako, A0452), CD8 (Abcam, ab178089, 1:100), CD4 (Abcam, ab133616, 1:100), CD20 (Dako, L26, IR604), CD56 (Abcam, ab75813, 1:100), FOXP3 (Abcam, ab20034, 1:100) and pan-CK (Abcam, ab7753, 1:100) (Akoya Biosciences). Next, secondary antibodies and reactive Opal fluorophores were incubated. DAPI was used to stain the nucleic acids. Multiplex stained slides were scanned at 20 nm wavelength intervals from 440 nm to 780 nm with a fixed exposure time and an absolute magnification of 200 utilizing an Akoya Biosciences Vectra Polaris Quantitative Pathology Imaging System. All scans for each slide were then superimposed to create a single image. Multilayer images were imported into APTIME software (3D Medicines Inc.) for quantitative image analysis. Pan-CK staining was used to distinguish the tumor parenchyma and stroma. The numbers of stained cells per square millimeter in all nucleated cells were used to express the amounts of distinct cell types.

### Statistical analysis

The demographic characteristics of patients were compared *via* the Chi-Square (χ2) test. PFS was analyzed using the Kaplan-Meier method with the log-rank test and drawn with R (version 4.0.2, R Development Core Team). Cox regression was implemented to calculate the HR for PFS, in both univariable and multivariable analyses. Interaction tests were performed to explore the interaction effect between Amp11q13 and treatment choice (PD-1Ab vs. nonPD-1Ab). Differences in immune cell subsets between the Amp11q13 group and the non-Amp11q13 group were analyzed using the Mann–Whitney U test. All statistical tests were double-sided; *p ≤*0.05 was considered significant.

## Results

### Patient characteristics and treatment groups

Between January 2019 and March 2022, 62 patients with HCC were enrolled in this study, including 46 with initially resected HCC and 16 with uHCC. Tissue samples accounted for 87.1% of all analyzed samples and liquid biopsy specimens were the rest. During the follow-up period, 17 patients who underwent previous resection showed recurrence, that was not suitable for operation and were classified into the uHCC group (**Figure 1**). Among all patients, the median age was 55.5 years (range, 30–76) and 16.1% (10/62) were women. Most patients did not have portal vein tumor thrombosis (PVTT) (49, 79.0%) and extrahepatic metastases (58, 93.5%) (**Table 1**). 33 patients with uHCC status were divided into PD-1Ab group (*n*=20) and nonPD-1Ab group (*n*=13). The baseline characteristics of these 33 patients for survival analysis are summarized in **Supplementary Table 2**.

**Table 1:** Baseline characteristics of the enrolled 62 patients.

Characteristics	Number of Cases(%)	Number of Amp11q13 Cases(%)	Number of nonAmp11q13 Cases(%)	chiq P-value
Age				0.19
<=60	41(66.1)	12(80)	29(61.7)	
>60	21(33.9)	3(20)	18(38.3)	
Gender				0.74
female	10(16.1)	2(13.3)	8(17)	
male	52(83.9)	13(86.7)	39(83)	
Cirrhosis				0.95
Non	8(12.9)	2(13.3)	6(12.8)	
Yes	54(87.1)	13(86.7)	41(87.2)	
AFP				0.1
<200	33(53.2)	5(33.3)	28(59.6)	
>=200	27(43.5)	10(66.7)	17(36.2)	
NA	2(3.2)		2(4.3)	
DCP				0.02
<200	17(27.4)	2(13.3)	15(31.9)	
>=200	35(56.5)	13(86.7)	22(46.8)	
NA	10(16.1)		10(21.3)	
BCLC.stage				0.08
A/B	44(71)	8(53.3)	36(76.6)	
C	18(29)	7(46.7)	11(23.4)	
Tumor.number				0.02
1	40(64.5)	6(40)	34(72.3)	
multiple	22(35.5)	9(60)	13(27.7)	
Size				0.58
<=5	27(43.5)	5(33.3)	22(46.8)	
>5	30(48.4)	9(60)	21(44.7)	
NA	5(8.1)	1(6.7)	4(8.5)	
Portal.vein.tumor.thrombosis				0.0004
Non	49(79)	7(46.7)	42(89.4)	
Yes	13(21)	8(53.3)	5(10.6)	
Extrahepatic.metastases				0.97
YES	4(6.5)	1(6.7)	3(6.4)	
non	58(93.5)	14(93.3)	44(93.6)	
HBsAg				0.64
0	10(16.1)	3(20)	7(14.9)	
1	52(83.9)	12(80)	40(85.1)	
HBV.DNA				0.86
>0	26(41.9)	6(40)	20(42.6)	
0	36(58.1)	9(60)	27(57.4)	
TB				0.19
<13	21(33.9)	3(20)	18(38.3)	
>=13	41(66.1)	12(80)	29(61.7)	
ALB				/
<50	62(100)	15(100)	47(100)	
ALT				0.67
<41	40(64.5)	9(60)	31(66)	
>=41	22(35.5)	6(40)	16(34)	
AST				0.08
<38	36(58.1)	5(33.3)	31(66)	
>=38	24(38.7)	9(60)	15(31.9)	
NA	2(3.2)	1(6.7)	1(2.1)	
type				0.55
HCC	29(46.8)	6(40)	23(48.9)	
uHCC	33(53.2)	9(60)	24(51.1)	
Treatment.Strategies				0.89
1st Line PD1Ab	15(24.2)	4(26.7)	11(23.4)	
2nd Line PD1Ab	5(8.1)	1(6.7)	4(8.5)	
LRT	5(8.1)	1(6.7)	4(8.5)	
NA	29(46.8)	6(40)	23(48.9)	
TKIs with or without LRT	8(12.9)	3(20)	5(10.6)	

### Genomic characteristics

**Figure 2:**
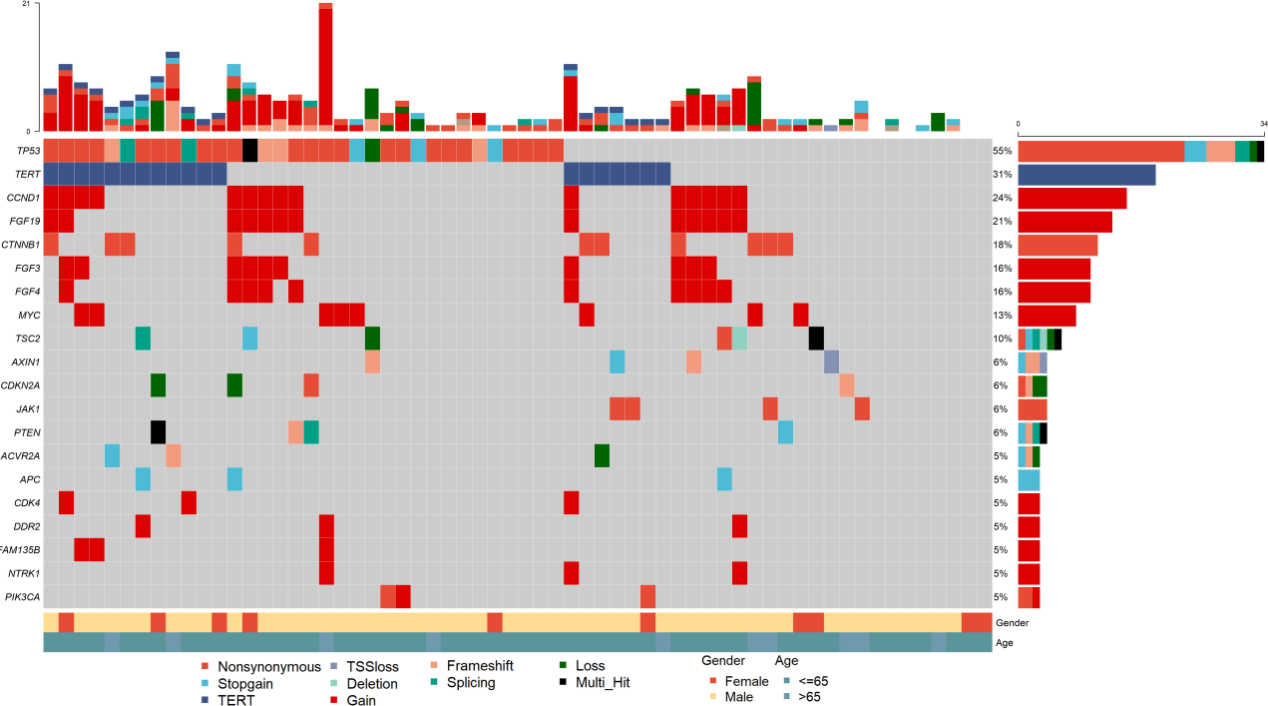
Summary of frequently (Top 20) genomic characterized alterations among 62 patients with HCC.

The mutation profiles of all 62 patients are depicted. 54 (87.1%) individuals had at least one pathogenic or likely pathogenic alteration in DNA. The most frequently altered gene was TP53, which was observed in 54.8% of the patients (34/62), followed by TERT, CCND1, FGF19, and CTNNB1. The common gene with copy number amplification were CCND1, FGF19, FGF3 and FGF4 (**Figures 2**, **S1, S2**). These four genes were located in the chromosome 11q13 locus, also known as Amp11q13. In our dataset, 15 (24.2%) patients harbored Amp11q13. The clinical characteristics of the Amp11q13 and non-Amp11q13 groups are presented in **Table 1**. Patients with Amp11q13 showed higher level of Des-γ-carboxy-prothrombin (DCP), tumor number and were more prone to be combined with portal vein tumor thrombosis (PVTT, all *P* < 0.05). There was no significant difference in the molecular profiling distribution between Amp11q13 and non-Amp11q13 patients (**Figure S3**).

### Association of Amp11q13 with the clinical outcome

**Figure 3:**
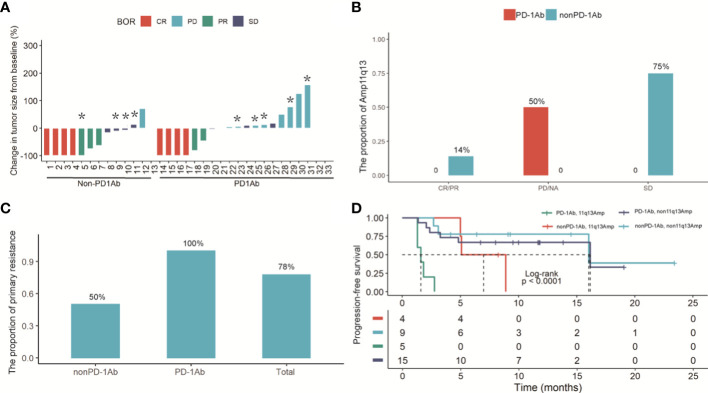
**(A)** Waterfall plot of the maximum reduction in target lesion diameter in 33 patients with recurrent HCC and initially diagnosed uHCC. Lesions caused by the death were not evaluable in 3 patients. **(B)** Histogram analysis of the proportion of the Amp11q13 and tumor response in PD-1Ab and nonPD-L1Ab groups. **(C)** The proportion of primary resistance in patients with Amp 11q13 among total patients, patients with the PD-1Ab and patients with nonPD-1Ab. * refer to patients with Amp11q13. **(D)** Kaplan-Meier estimates of PFS in Amp11q13 and nonAmp11q13 patients in PD-1Ab and Non-PD-1Ab treatment group.

Thirty-three patients with recurrent HCC and initially diagnosed uHCC had a median follow-up period of 11 months (interquartile range 5.9‐21.5). For 20 patients in the PD-1Ab group, the median PFS was 10.5 months (95% CI 2.37–NE). The median PFS was 16.0 months (95% CI 5.10–NE) in the nonPD-1Ab group. All patients with nonPD-1Ab in our study received first-line therapy (**Figure S4**). Tumor shrinkage was observed in 18 (54.5%) of 33 patients with baseline and post-baseline assessments (**Figure 3A**). Of the 20 patients who were evaluable for response in the PD-1Ab group, 6 (30%) patients had an objective response. Response was not determined in three patients because of withdrawal for clinical deterioration before an initial response assessment. The Amp11q13 rate was more common in the PD-1Ab group with PD (50%) and the nonPD-1Ab group with stable disease (SD) (75%) (**Figure 3B**). In the PD-1Ab group, the proportion of patients with PD as BOR in patients with Amp11q13 was significantly higher than that in patients with nonAmp11q13 (100% vs 33.3%, *P*=0.03, **Table S4**). In the nonPD-1Ab group, the proportion of PD in patients with Amp11q13 and nonAmp11q13 had no significant difference (0% vs 11.1%, p>0.99, **Table S4**). Notably, all patients with Amp11q13 who received PD-1Ab showed primary resistance (**Figure 3C**).

By repeating Fisher’s exact test, the association of each gene with the response to systemic therapy was analyzed in detail. Notably, all the patients who harbored amplification of the either one gene (*FGF3*, *FGF4*, *FGF19*, or *CCND1*) in the 11q13 amplicon developed progressive disease (PD) upon ICI treatment. However, only *CCND1* was significantly associated with poor response to ICI treatment (*P* = 0.033). In the nonPD-1Ab group, no such correlation was found.

Kaplan‐Meier curves showed that Amp11q13 was associated with inferior PFS, regardless of the treatment strategy. Notably, patients who received immunotherapy with Amp 11q13 had the shortest PFS compared with other groups (log rank *P* < 0.001) (**Figure 3D**).

**Figure 4:**
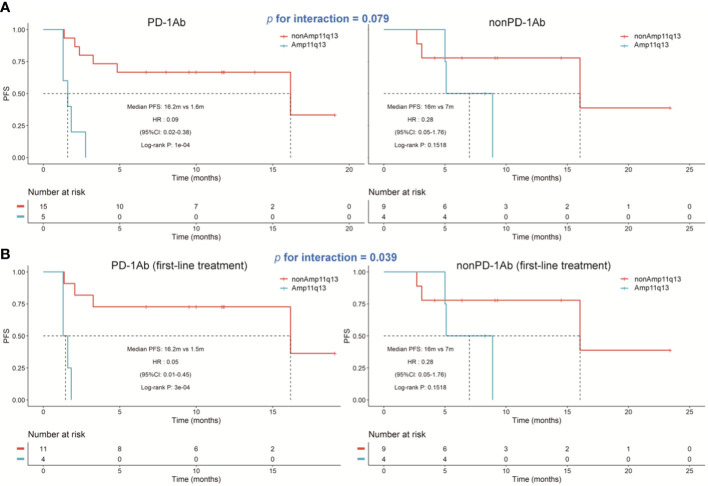
Kaplan‐Meier estimates of progression‐free survival. **(A)** Kaplan‐Meier survival curves of progression-free survival comparing PD-1Ab therapy and nonPD-1Ab therapy. **(B)** Kaplan‐Meier survival curves of progression‐free survival comparing first-line PD-1Ab therapy and nonPD-1Ab therapy.

In the PD-1Ab group, patients with Amp11q13 presented significantly shorter PFS than those without Amp11q13 (adjusted hazard ratio [HR], 0.09; 95% CI, 0.02-0.38; *P*<0.0001), while no such correlation was observed in the nonPD-1Ab group (adjusted hazard ratio [HR], 0.28; 95% CI, 0.05-1.76; *P*=0.15). However, there was no significant interaction in PFS between these two groups (*P* for interaction =0.0794, **Figure 4A**). No association was found between Amp11q13 and the prognosis in The Cancer Genome Atlas (TCGA) database (**Figure S5**).

We also analyzed the difference in the Amp11q13 effect on PFS between patients receiving first-line PD-1Ab and patients with nonPD-1Ab (**Figure 4B**). In the PD-1Ab group, the median PFS was 1.5 months in Amp 11q13 patients vs 16.2 months in nonAmp 11q13 patients (HR, 0.05; 95% CI 0.01-0.45; *P* = 0.0003). In the nonPD-1Ab group, the median PFS was 7.0months in Amp 11q13 patients vs 16.0 months in nonAmp 11q13 patients (HR, 0.28; 95% CI 0.05-1.76; *P* = 0.15). The interaction between Amp 11q13 status and treatment was significant (*P* for interaction = 0.0389). In the multivariable model, Amp11q13 status was the only independent predictor for PFS in the PD-1Ab group (HR, 17.83; 95% CI 2.93-108.4; *P* = 0.002) (**Table 2**).

**Table 2:** Univariable and multivariable analysis of PFS in PD-1Ab group.

Characteristics	Univariate analysis			Multivariate analysis		
	Hazard.Ratio	CI95	P.Value.	Hazard.Ratio	CI95	P.Value
AFP	1.07	0.27-4.14	0.926			
Age	1	0.26-3.85	1			
ALT	0.69	0.19-2.46	0.569			
AST	1.59	0.46-5.45	0.462			
BCLC	1.09	0.32-3.78	0.89			
Cirrhosis	0.66	0.17-2.54	0.541			
Amp11q13	11.59	2.63-51.09	0.001	17.83	2.93-108.4	0.002
DCP	1.07	0.23-5.06	0.931			
Extrahepatic.metastases	0.26	0.03-2.09	0.206			
Gender	0.8	0.17-3.77	0.777			
HBsAg	0.64	0.13-3.03	0.572			
HBV.DNA	2.05	0.43-9.76	0.365			
Portal.vein.tumor.thrombosis	2.65	0.76-9.26	0.127	2.09	0.52-8.37	0.297
Size	2.99	0.63-14.12	0.168	3.62	0.57-23.15	0.173
TB	2.67	0.57-12.46	0.212			
Treatment.Strategies	1.04	0.27-4.04	0.955			
Tumor.number	3.79	0.48-30.01	0.206			

We also conducted repeated survival analyses to determine the contribution of each gene in predicting the prognosis. We discovered that the amplification of the either one gene (*FGF3*, *FGF4*, *FGF19*, or *CCND1*) predicted inferior PFS on PD-1Ab treatment separately (all *P* values < 0.05), but this association was not observed in patients treated with nonPD-1Ab.

### Tumor microenvironment characteristics in patients with and without Amp11q13

**Figure 5:**
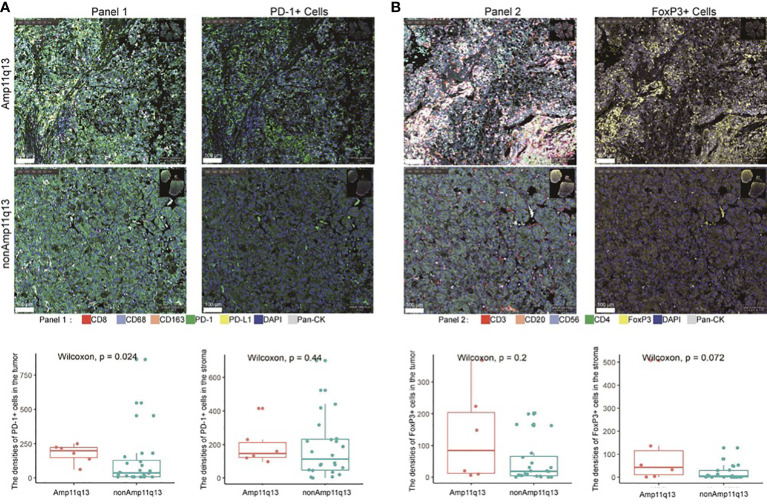
Relationship between immune cell subsets in tumor immune microenvironment and Amp11q13 status. Images of representative mIHC results (top, scale: 500 μm) were showed in two patients with and without Amp11q13. PD-1+ cells and FoxP3+ cells (bottom) in patients with Amp11q13 compared with nonAmp11q13 patients were also displayed. **(A)** Panel 1 referred to multiple stained slides and representative merged images show the distribution of individual markers [CD8, CD163, CD68, PD-1, PD-L1, pan-CK, DAPI for nucleic acids (blue)] and the separable vision of immunohistochemical staining with PD-1 antibody (green). **(B)** Panel 2 referred to multiple stained slides and representative merged images show the distribution of individual markers [CD3, CD20, FoxP3, CD56, CD4, pan-CK, DAPI for nucleic acids (blue)] and the separable vision of immunohistochemical staining with FoxP3 antibody (yellow).

To investigate the difference in the tumor immune microenvironment between HCC patients with and without Amp11q13, we assessed 30 FFPE samples that were subjected to both NGS and mIHC. Six of these harbored Amp11q13. Compared with samples without Amp11q13, samples with Amp11q13 were associated with higher densities of PD-1^+^ cells in the tumor (*P*=0.024) (**Figure 5A**). Similar results were also observed for densities of FoxP3^+^ cells in the stroma, despite that the significance of the difference was limited by the small sample size (*P*=0.072) (**Figure 5B**). No remarkable differences were observed in the density of other immune cell subsets in the tumor microenvironment (**Figure S6**).

To validate the findings, data from the TCGA (283 patients with HCC, include 24 with Amp11q13 and 259 with nonAmp11q13) were analyzed. The results showed that the expression of PD-1 and the infiltration of Treg cells was slightly higher in the Amp11q13 group compared to the nonAmp11q13 group, which is consistent with the findings of our study.

### Hyper-progressive disease case series

HPD was observed in 3 of 20 patients (15.0%) (**Table 3**). All three cases were 11q13 amplified, less than 75 years old, without extra-hepatic metastases, and had a good ECOG performance status (0-1). Most importantly, all three cases were with Amp11q13. The proportion of HPD in patients with Amp11q13 was significantly higher than patients with nonAmp11q13 in the PD-1Ab group (3/5, 60% vs 0/15, 0%, *P*=0.0075). No HPD was found in the nonPD-1 group.

**Table 3:** HPD evaluation of 5 patients with Amp11q13 receiving immunotherapy.

Patient ID	Line	TTF<2 months	TGK	TGR	Tumor Burden Increase%	HPD
31	2	yes	7.91	6.2	593.667	yes
29	1	yes			579.527	yes
25	1	yes			35.1115	no
23	1	yes			9.41931	no
26	1	yes			53.4903	yes

#### Case 1

**Figure 6:**
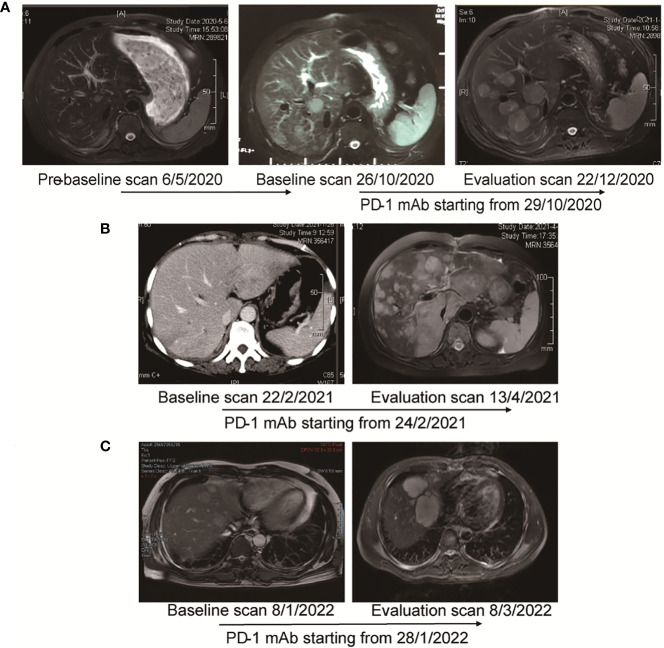
MR images and the timeline of therapy in three cases with HPD. **(A)** Case 1. Pre-baseline MR was obtained before ablation. After confirmed progression 5 months later, the patient adopted immunotherapy. Evaluation scan after 1.8 months showed significant radiological progression of disease with occurrence of new lesions. The TGR fold was calculated to be 11.90. **(B)** Case 2. After 2 cycles of immunotherapy, MR showed significant progression of intra-hepatic lesions. TTF was 1.6 months. TBI was 579.5%. **(C)** Case 3. After 2 cycles of treatment, MR scan showed significant progression of disease with increase in size of hepatic lesions. TTF was 1.3 months. TBI was 53.5%.

This 61-year-old man was first diagnosed with HCC in segment 6 and underwent local resection in 2017. He had a local recurrence of disease in segment 7 and the left lateral lobe and underwent trans-arterial chemoembolization (TACE) in December 2017. Apatinib (a selective VEGFR-2 TKI, Hengrui, Jiangsu, China) was administered subsequently. However, MR showed 6 new lesions, with a maximum diameter of 2.8 cm in May 2020. Ablation therapy were performed thereafter. Unfortunately, further progression led to the development of 3 new lesions 5 months later. He was referred to TACE plus the PD-1 monoclonal antibody toripalimab in late October 2019. MR scan performed 1.8 months after the initiation of ICI showed significant radiological progression of disease with the occurrence of new lesions (totally >= 15). His TGK ratio was 11.95, and the TGR fold was 11.90 (**Figure 6A**). CtDNA test showed amplification of 11q13. Toripalimab was ceased thereafter and a therapy strategy containing hepatic artery infusion chemotherapy (HAIC) and regorafenib was given. Notably, the lesions were reduced following the cessation of ICI therapy. In March 2022, regorafenib was suspended for acute myocardial infarction. In June 2022, a dramatic progression of intra-hepatic lesions was observed by MR scan. Regorafenib was then continued. This patient was alive until the cut-off date.

#### Case 2

A 56-year-old woman was first diagnosed with HCC in January 2019. She underwent resection as an initial treatment. During the operation, a mass of 4.0 cm in diameter was found in in segment 7. Two years after surgery (February 2021), an abdominal CT scan showed a recurrence of HCC in the left lateral lobe, with a diameter of 5.2 cm. TACE plus PD-1 monoclonal antibody sintilimab (Innovent, Suzhou, China) was given. Imaging performed after two cycles of treatment (April 2021) demonstrated significant radiological progression of disease with an increase in the number and size of hepatic lesions, accompanied with the occurrence of pulmonary lesions (**Figure 6B**). TTF was 1.6 months. HPD was diagnosed for a TBI of 579.5%. The NGS test based on the resected specimen showed an amplification of 11q13. PD-1 immunotherapy was ceased, and HAIC was given subsequently. However, she continued to deteriorate and passed away in June 2021.

#### Case 3

The 41-year-old man went to the local clinic for abdominal pain in December 2021. MR scan showed a large infiltrative mass involving almost the entire left hepatic lobe, with several nodules of 0.6 cm to 2.8 cm in the remaining left medial lobe, the right anterior S8 lobe and the S1-caudate lobe. Tumor thrombosis was seen in the left and main portal vein. He was diagnosed with locally extensive infiltrative and multifocal HCCs. Genetic sequencing of tumor biopsy showed the amplification of CCND1, FGF3 and MYC and somatic mutation of TP53 and TERT. Stereotactic body radiotherapy focusing on PVTT was performed 6 times from Month 2022, combined with oral lenvatinib and intravenous pembrolizumab. Two doses of pembrolizumab were administered when he was referred to our hospital. Unfortunately, as shown by the MR scan, the tumor progressed with a significant increase in the size of the multifocal hepatic lesions (**Figure 6C**). TTF was 1.3 months. HPD was diagnosed for a TBI of 53.5%. PD-1 immunotherapy was ceased and TACE was given. He passed away 4 months later.

## Discussion

In this study, we retrospectively analyzed the clinical features and genomic profiles of 62 HCC patients in our institution. The total prevalence rate of Amp11q13 was 24.2%, which appeared more frequently in patients with poor clinical indicators. In patients with the primary resistance to systemic therapy, the proportion of Amp11q13 was 43.8%. Furthermore, three of 13 patients with primary resistance to PD-1 blockade therapy showed HPD, as proved by assessing tumor growth dynamics. All HPD patients showed Amp11q13.

Immunotherapy, represented by ICIs, has entered the therapeutic arsenal for many advanced-stage malignancies (35). ICIs reactivate exhausted T cells by targeting their immunological synapse. Besides, ICIs are associated with novel patterns of anti-tumor efficacy, such as longer duration of response, pseudoprogression (an initial disease progression followed by objective response), and even HPD (12). However, a notable number of individuals remain resistant to PD-1 blockade. In phase II and randomized phase III trials, PD-1 monoclonal antibodies showed a consistent 15−20% response rate in patients with HCC(7, 8). Approximately 30~40% of patients showed primary resistance to PD-1 blockade therapy.

The following pathways may contribute to PD-1 blockade therapeutic resistance: the absence of antigenic proteins, impaired antigen presentation, lack of T cells with tumor antigen-specific T-cell receptors, insensitivity to T cells (mutations in the interferon gamma pathway), overexpression of other inhibitory immune checkpoints and immunosuppressive cell recruitment (17, 36–38). Resistance to ICIs may also be caused by genetic T cell exclusion. For instance, oncogenic signaling through the MAPK pathway results in the production of VEGF and IL-8, which have known inhibitory effects on T cell recruitment and function (39). In a murine model, tumors with elevated beta-catenin lacked a subset of dendritic cells (DCs) known as CD103+ DCs, due to decreased expression of CCL4, a chemokine that attracts CD103+ DCs (40). Activation of the CDK4/6 pathway has also been found to be associated with resistance to PD-1 blockade. Whole-exome sequencing in patients with metastatic melanoma treated with anti-PD-1 antibodies revealed CDK4 amplification in patients with no clinical improvement. Defective TNF-alpha signaling, inflammatory response, and IFN-gamma response were found in tumors with CDK4 amplification (18). A bioinformatic analysis of the TCGA and MSKCC datasets revealed that CCND1 amplification was associated with shorter overall survival and inferior outcomes with ICIs (19). Furthermore, CCND1 amplification was associated with immune cell exclusion and a variety of aggressive, immunosuppressive oncogenic pathways, including KRAS and mTOR signaling (19). By using single-cell RNA sequencing for 33 melanoma tumor specimens, Jerby-Arnon et al. developed a cancer cell program to predict T cell exclusion and resistance to checkpoint blockade (41). Interestingly, CDK appears to be the master regulators that governs the expression of the genes in the program. In our study, we sought to determine the genetic factors that conferred responsiveness to PD-1 blockade in HCC. Our results confirmed the role of CCND1 amplification as a pivotal regulator of PD-1 resistance in HCC.

CCND1 is a downstream effector in the Wnt2/β-catenin pathway and activates the cyclin-dependent kinases (CDKs) CDK4 and CDK6 to promote cancer proliferation (42). FGF signaling promotes cancer proliferation, migration and angiogenesis by downstream activation of the RAS–RAF–MAPK, PI3K–AKT, signal transducer and activator of transcription and phospholipase Cγ pathways. The locus CCND1-ORAOV1-FGF19-FGF4-FGF3-TMEM16A-FADD-PPFIA1-CTTN was located on chromosome 11q13. The amplifications of CCND1, FGF3, FGF4 and FGF19 were characterized as Amp11q13 (43–45). Indeed, Amp11q13 is present in many cancers, including esophageal cancer, head and neck tumors, sarcoma, breast cancer, bladder tumors and hepatobiliary malignancies (43). Amp11q13 was most prevalent in head and neck squamous cell carcinoma, with an incidence of 30-36% (46, 47). In individuals with head and neck squamous cell carcinoma, Amp11q13 was also associated with decreased PFS (48). In HCC, Amp 11q13 was found in approximately 10% of the population (49–51). In the current study, we found that Amp11q13 was associated with a worse prognosis in the PD-1Ab group. Especially for patients with first-line treatment, there was a significant interaction between Amp11q13 status and treatment. In the multivariable analysis, Amp11q13 was an independent predictor of PFS in the PD-1Ab group.

Several immune cell subtypes in the tumor microenvironment have recently been found to be correlated with combination treatment with ICIs and antiangiogenic drugs in HCC (9, 52). Patients with a high infiltration of M1 macrophages had a significantly longer PFS and OS, suggesting that the high infiltration of M1 macrophages in HCC might be a potential positive indicator for the efficacy of immunotherapy (53). In our study, increased densities of PD-1+ cells in the tumor and FoxP3+ cells in the stroma were observed in patients with Amp11q13 compared with patients without Amp11q13. It was reported that effector Treg cells were activated and thus mediated suppression of antitumor immunity in HPD patients with gastric cancer (54). HPD might be triggered by activating Tregs in 11q13 amplified patients. However, the mechanisms through which tumor cell with 11q13 amplification induce the infiltration of Tregs are still needed to be elucidated. These findings showed that PD-1+ cells and FoxP3+ cells might be associated with hampered immunological response of HCC patients.

Patients with Amp11q13 may be sensitive to therapies other than immunotherapy. It was reported that FGF19, a biomarker of proliferation propensity, is an important driver gene in HCC (55, 56). HCC harboring FGF19 amplification may represent a subset of cancers that are strongly addicted to the FGFR pathway. The interaction of FGF19 and FGFR4 has been exploited for drug development. In FGF19-positive patients as measured by Immunohistochemistry, the overall response rate of FGFR4 antibody fisogatinib was 17% with a median duration of response of 5.3 months (57), while 0% in FGF19-negative patients. In addition, FGF3/FGF4 amplification was found to predict an increased response to sorafenib in patients with HCC (58). In addition, CCND1 could be targeted by palbociclib (59). These findings support future exploration of individualized combined targeted therapy based upon genetic sequencing in clinical setting.

Defined by its signal growth kinetics, HPD has raised many concerns regarding its incidence, mechanism, and potential biomarkers. Some debates that HPD represents the natural accelerating growth pace of the disease course (25). HPD after ICIs treatment in patients with Amp11q13 has been reported in non-small cell lung cancer, esophageal adenocarcinoma and lung cancer with neuroendocrine features. The incidence of hyperprogression was 43% in patients with 11q13 amplification (60). Previously, MDM2/4 amplification, EGFR alterations, and loss of CDKN2A/B have also been reported as potential biomarkers for HPD (61, 62). It was revealed that 12.7% of nivolumab-treated patients with HCC had HPD. The potential risk factors for HPD in HCC include previous radiotherapy (63), elevated neutrophil-to-lymphocyte ratio and hemoglobin level, PVTT and Child-Pugh score (64–66). However, little is known about whether HPD is associated with somatic mutations in HCC. In our study, the incidence of HPD was 60.0% in patients receiving ICIs with Amp11q13, supporting the role of Amp11q13 as an HPD predictor.

Currently, most investigators recommend the withdrawal of PD-1 blockade therapy and consider other potentially effective therapies upon HPD (23). In the first case, once ICI-related HPD was diagnosed, we stopped immunotherapy immediately, and HAIC and regorafenib were given subsequently. Imaging study following the adjustment of treatment strategy showed remarkable tumor shrinkage (the maximal shrinkage rate was 29.3%, compared with the HPD imaging). However, in another two patients with deteriorating liver function, salvage therapies failed to improve the survival.

There are several limitations to our study. First, limited number of patients and lack of external validation may lead to bias. From January 2019 to March 2022, only 33 cases of uHCC with complete clinical and genotypic data met the inclusion criteria. Second, it should be noted that due to the progressive nature of advanced stage malignancy, it is difficult to dissociate HPD from primary resistance to immunotherapy, even though a specific growth kinetic model was used. Third, our findings of mIHC lacked an exploration of the relationship between treatment efficacy and immune cell infiltration due to the limitations of sample size. Therefore, we are unable to conclusively demonstrate how Amp11q13 affects primary resistance and HPD.

## Conclusions

In conclusion, we found that Amp11q13 was seen more frequently in advanced stage HCC patients, and was associated with HPD in patients receiving anti-PD-1 immunotherapy. These findings may help guide the use of immunotherapy for HCC in routine clinical practice. Patients with Amp11q13 are less likely to benefit from PD-1 blockade therapies. Other options, including locoregional therapies, such as HAIC, and other systemic regimens, including CDK4/6 inhibitor, FGFR4 antibody might be considered as potential effective strategies. Further studies are urgently needed to validate the value of NGS in the prediction of PD-1 blockade responsiveness and interrogate the mechanism of resistance in patients with certain genetic aberrations.

## Data availability statement

The datasets presented in this study can be found in online repositories. The names of the repository/repositories and accession number(s) can be found below: OMIX repository (OMIX002465), BioProject ID: PRJCA013528, accession: https://ngdc.cncb.ac.cn/omix/release/OMIX002465.

## Ethics statement

The studies involving human participants were reviewed and approved by the Chinese Ethics Committee of Registering Clinical Trials. The patients/participants provided their written informed consent to participate in this study. Written informed consent was obtained from the individual(s) for the publication of any potentially identifiable images or data included in this article.

## Author contributions

HZ, KY and DZ designed the study. YC and DZ acquired and analyzed the NGS and mIHC data. Data collection and data cleansing with clinical information were executed by KY and JL. KY, DZ and YC wrote the manuscript. HZ, WL, YF, MH, JC, SC, TB and YB critically reviewed the manuscript. All authors contributed to the article and approved the submitted version.

## Supplementary material

Supplementary Figure 1 Summary of frequently (Top 20) genomic characterized copy number alterations among 62 patients with HCC.

Supplementary Figure 2 Summary of frequently (Top 20) genomic characterized single-nucleotide variants among 62 patients with HCC.

Supplementary Figure 3 The molecular profiling distribution between Amp11q13 and nonAmp11q13 patients

Supplementary Figure 4 Treatment efficacy in 33 patients with PD-1Ab group and nonPD-1Ab group

Supplementary Figure 5 Kaplan-Meier curves of PFS comparing Amp11q13 and nonAmp11q13 groups in TCGA cohort.

Supplementary Figure 6 Immune cell subsets in the tumor microenvironment for patients in Amp11q13 and nonAmp11q13 groups.

Supplementary Figure 7 Comparison of the expression of PD-1 and infiltration of Treg cells in TCGA database.

Supplementary Table 1 Specific treatment strategies for patients in PD-1Ab group.

Supplementary Table 2 Baseline characteristics of 33 patients with recurrent HCC and initially diagnosed uHCC.

Supplementary Table 3 Demonstration of patients’ response and the amplification status of individual genes within the 11q13 amplicon.

## References

[1] Siegel RL Miller KD Fuchs HE Jemal A. Cancer statistics 2021. CA: A Cancer J Clin (2021) 71:7–33. doi: 10.3322/caac.21654 33433946

[2] Llovet JM Kelley RK Villanueva A Singal AG Pikarsky E Roayaie S. Hepatocellular carcinoma. Nat Rev Dis Primers (2021) 7:6. doi: 10.1038/s41572-020-00240-3 33479224 PMC13537433

[3] Hassanipour S Vali M Gaffari-Fam S Nikbakht HA Abdzadeh E Joukar F. The survival rate of hepatocellular carcinoma in Asian countries: A systematic review and meta-analysis. Excli J (2020) 19:108–30. doi: 10.17179/excli2019-1842 PMC7003639 32038120

[4] European Association For The Study Of The Liver. EASL-EORTC clinical practice guidelines: Management of hepatocellular carcinoma. Journal of hepatology (2012) 56(4):908–43. doi: 10.1016/j.jhep.2011.12.001 22424438

[5] Park JW Chen M Colombo M Roberts LR Schwartz M Chen PJ. Global patterns of hepatocellular carcinoma management from diagnosis to death: The BRIDGE study. Liver Int (2015) 35:2155–66. doi: 10.1111/liv.12818 PMC4691343 25752327

[6] Ribas A Wolchok JD. Cancer immunotherapy using checkpoint blockade. Science (2018) 359:1350–5. doi: 10.1126/science.aar4060 PMC7391259 29567705

[7] El-Khoueiry AB Sangro B Yau T Crocenzi TS Kudo M Hsu C. Nivolumab in patients with advanced hepatocellular carcinoma (CheckMate 040): An open-label, non-comparative, phase 1/2 dose escalation and expansion trial. Lancet (2017) 389:2492–502. doi: 10.1016/S0140-6736(17)31046-2 PMC7539326 28434648

[8] Zhu AX Finn RS Edeline J Cattan S Ogasawara S Palmer D. Pembrolizumab in patients with advanced hepatocellular carcinoma previously treated with sorafenib (KEYNOTE-224): A non-randomised, open-label phase 2 trial. Lancet Oncol (2018) 19:940–52. doi: 10.1016/S1470-2045(18)30351-6 29875066

[9] Finn RS Ikeda M Zhu AX Sung MW Baron AD Kudo M. Phase ib study of lenvatinib plus pembrolizumab in patients with unresectable hepatocellular carcinoma. J Clin Oncol (2020) 38:2960–70. doi: 10.1200/JCO.20.00808 PMC7479760 32716739

[10] Finn RS Qin S Ikeda M Galle PR Ducreux M Kim TY. Atezolizumab plus bevacizumab in unresectable hepatocellular carcinoma. N Engl J Med (2020) 382:1894–905. doi: 10.1056/NEJMoa1915745 32402160

[11] Cheng A-L Qin S Ikeda M Galle PR Ducreux M Kim T-Y. Updated efficacy and safety data from IMbrave150: Atezolizumab plus bevacizumab vs. sorafenib for unresectable hepatocellular carcinoma. J Hepatol (2022) 76:862–73. doi: 10.1016/j.jceh.2022.07.003 34902530

[12] Borcoman E Kanjanapan Y Champiat S Kato S Servois V Kurzrock R. Novel patterns of response under immunotherapy. Ann Oncol (2019) 30:385–96. doi: 10.1093/annonc/mdz003 30657859

[13] Hou W Yi C Zhu H. Predictive biomarkers of colon cancer immunotherapy: Present and future. Front Immunol (2022) 13:1032314. doi: 10.3389/fimmu.2022.1032314 36483562 PMC9722772

[14] Rizzo A Ricci AD Di Federico A Frega G Palloni A Tavolari S. Predictive biomarkers for checkpoint inhibitor-based immunotherapy in hepatocellular carcinoma: Where do we stand? Front Oncol (2021) 11:803133. doi: 10.3389/fonc.2021.803133 34976841 PMC8718608

[15] Rizzo A Cusmai A Gadaleta-Caldarola G Palmiotti G. Which role for predictors of response to immune checkpoint inhibitors in hepatocellular carcinoma? Expert Rev Gastroenterol Hepatol (2022) 16:333–9. doi: 10.1080/17474124.2022.2064273 35403533

[16] Viscardi G Tralongo AC Massari F Lambertini M Mollica V Rizzo A. Comparative assessment of early mortality risk upon immune checkpoint inhibitors alone or in combination with other agents across solid malignancies: A systematic review and meta-analysis. Eur J Cancer (2022) 177:175–85. doi: 10.1016/j.ejca.2022.09.031 36368251

[17] Schoenfeld AJ Hellmann MD. Acquired resistance to immune checkpoint inhibitors. Cancer Cell (2020) 37:443–55. doi: 10.1016/j.ccell.2020.03.017 PMC7182070 32289269

[18] Yu J Yan J Guo Q Chi Z Tang B Zheng B. Genetic aberrations in the CDK4 pathway are associated with innate resistance to PD-1 blockade in Chinese patients with non-cutaneous melanoma. Clin Cancer Res (2019) 25:6511–23. doi: 10.1158/1078-0432.CCR-19-0475 31375512

[19] Chen Y Huang Y Gao X Li Y Lin J Chen L. CCND1 amplification contributes to immunosuppression and is associated with a poor prognosis to immune checkpoint inhibitors in solid tumors. Front Immunol (2020) 11. doi: 10.3389/fimmu.2020.01620 PMC7438829 32903763

[20] Guo Y Yang J Ren K Tian X Gao H Tian X. The heterogeneity of immune cell infiltration landscape and its immunotherapeutic implications in hepatocellular carcinoma. Front Immunol (2022) 13:861525. doi: 10.3389/fimmu.2022.861525 35355983 PMC8959995

[21] Hong T Su W Pan Y Tian C Lei G. Aging-related features predict prognosis and immunotherapy efficacy in hepatocellular carcinoma. Front Immunol (2022) 13:951459. doi: 10.3389/fimmu.2022.951459 36189258 PMC9521435

[22] Champiat S Dercle L Ammari S Massard C Hollebecque A Postel-Vinay S. Hyperprogressive disease is a new pattern of progression in cancer patients treated by anti-PD-1/PD-L1. Clin Cancer Res (2017) 23:1920–8. doi: 10.1158/1078-0432.CCR-16-1741 27827313

[23] Champiat S Ferrara R Massard C Besse B Marabelle A Soria JC. Hyperprogressive disease: Recognizing a novel pattern to improve patient management. Nat Rev Clin Oncol (2018) 15:748–62. doi: 10.1038/s41571-018-0111-2 30361681

[24] Camelliti S Le Noci V Bianchi F Moscheni C Arnaboldi F Gagliano N. Mechanisms of hyperprogressive disease after immune checkpoint inhibitor therapy: What we (don't) know. J Exp Clin Cancer Res (2020) 39:236. doi: 10.1186/s13046-020-01721-9 33168050 PMC7650183

[25] Kang Y-K Reck M Nghiem P Feng Y Plautz G Kim HR. Assessment of hyperprogression versus the natural course of disease development with nivolumab with or without ipilimumab versus placebo in phase III, randomized, controlled trials. J ImmunoTherapy Cancer (2022) 10:e004273. doi: 10.1136/jitc-2021-004273 PMC8983994 35383114

[26] Yang N Li Y Liu Z Qin H Du D Cao X. The characteristics of ctDNA reveal the high complexity in matching the corresponding tumor tissues. BMC Cancer (2018) 18:319. doi: 10.1186/s12885-018-4199-7 29566644 PMC5865353

[27] Li H Durbin R. Fast and accurate short read alignment with burrows-wheeler transform. Bioinformatics (2009) 25:1754–60. doi: 10.1093/bioinformatics/btp324 PMC2705234 19451168

[28] Su D Zhang D Chen K Lu J Wu J Cao X. High performance of targeted next generation sequencing on variance detection in clinical tumor specimens in comparison with current conventional methods. J Exp Clin Cancer Res (2017) 36:121. doi: 10.1186/s13046-017-0591-4 28882180 PMC5590190

[29] Lencioni R Llovet JM. Modified RECIST (mRECIST) assessment for hepatocellular carcinoma. Semin Liver Dis (2010) 30:52–60. doi: 10.1055/s-0030-1247132 20175033 PMC12268942

[30] Salkeni MA Shin JY Gulley JL. Resistance to immunotherapy: Mechanisms and means for overcoming. Adv Exp Med Biol (2021) 1342:45–80. doi: 10.1007/978-3-030-79308-1_2 34972962

[31] Le Tourneau C Servois V Diéras V Ollivier L Tresca P Paoletti X. Tumour growth kinetics assessment: Added value to RECIST in cancer patients treated with molecularly targeted agents. Br J Cancer (2012) 106:854–7. doi: 10.1038/bjc.2012.10 PMC3305968 22281665

[32] Saâda-Bouzid E Defaucheux C Karabajakian A Coloma VP Servois V Paoletti X. Hyperprogression during anti-PD-1/PD-L1 therapy in patients with recurrent and/or metastatic head and neck squamous cell carcinoma. Ann Oncol (2017) 28:1605–11. doi: 10.1093/annonc/mdx178 28419181

[33] Ferté C Fernandez M Hollebecque A Koscielny S Levy A Massard C. Tumor growth rate is an early indicator of antitumor drug activity in phase I clinical trials. Clin Cancer Res (2014) 20:246–52. doi: 10.1158/1078-0432.CCR-13-2098 PMC3947306 24240109

[34] Wolchok JD Hoos A O'day S Weber JS Hamid O Lebbé C. Guidelines for the evaluation of immune therapy activity in solid tumors: immune-related response criteria. Clin Cancer Res (2009) 15:7412–20. doi: 10.1158/1078-0432.CCR-09-1624 19934295

[35] Llovet JM Castet F Heikenwalder M Maini MK Mazzaferro V Pinato DJ. Immunotherapies for hepatocellular carcinoma. Nat Rev Clin Oncol (2022) 19:151–72. doi: 10.1038/s41571-021-00573-2 34764464

[36] Sharma P Hu-Lieskovan S Wargo JA Ribas A. Primary, adaptive, and acquired resistance to cancer immunotherapy. Cell (2017) 168:707–23. doi: 10.1016/j.cell.2017.01.017 PMC5391692 28187290

[37] Jackson CM Choi J Lim M. Mechanisms of immunotherapy resistance: lessons from glioblastoma. Nat Immunol (2019) 20:1100–9. doi: 10.1038/s41590-019-0433-y 31358997

[38] Billan S Kaidar-Person O Gil Z. Treatment after progression in the era of immunotherapy. Lancet Oncol (2020) 21:e463–76. doi: 10.1016/S1470-2045(20)30328-4 33002442

[39] Liu C Peng W Xu C Lou Y Zhang M Wargo JA. BRAF inhibition increases tumor infiltration by T cells and enhances the antitumor activity of adoptive immunotherapy in mice. Clin Cancer Res (2013) 19:393–403. doi: 10.1158/1078-0432.CCR-12-1626 23204132 PMC4120472

[40] Spranger S Bao R Gajewski TF. Melanoma-intrinsic β-catenin signalling prevents anti-tumour immunity. Nature (2015) 523:231–5. doi: 10.1038/nature14404 25970248

[41] Jerby-Arnon L Shah P Cuoco MS Rodman C Su MJ Melms JC. A cancer cell program promotes T cell exclusion and resistance to checkpoint blockade. Cell (2018) 175:984–997.e924. doi: 10.1016/j.cell.2018.09.006 30388455 PMC6410377

[42] Jin Y Jin C Law S Chu KM Zhang H Strombeck B. Cytogenetic and fluorescence *in situ* hybridization characterization of clonal chromosomal aberrations and CCND1 amplification in esophageal carcinomas. Cancer Genet Cytogenet (2004) 148:21–8. doi: 10.1016/S0165-4608(03)00213-9 14697637

[43] Katoh M Katoh M. Comparative genomics on mammalian Fgf3-Fgf4 locus. Int J Oncol (2005) 27:281–5. doi: 10.3892/ijo.27.1.281 15942670

[44] Katoh M Katoh M. Identification and characterization of TMEM16H gene in silico. Int J Mol Med (2005) 15:353–8. doi: 10.3892/ijmm.15.2.353 15647853

[45] Brown J Bothma H Veale R Willem P. Genomic imbalances in esophageal carcinoma cell lines involve wnt pathway genes. World J Gastroenterol (2011) 17:2909–23. doi: 10.3748/wjg.v17.i24.2909 PMC3129505 21734802

[46] Williams ME Gaffey MJ Weiss LM Wilczynski SP Schuuring E Levine PA. Chromosome 11Q13 amplification in head and neck squamous cell carcinoma. Arch Otolaryngol Head Neck Surg (1993) 119:1238–43. doi: 10.1001/archotol.1993.01880230084013 8217084

[47] Hermida-Prado F Menéndez ST Albornoz-Afanasiev P. Distinctive expression and amplification of genes at 11q13 in relation to HPV status with impact on survival in head and neck cancer patients. J Clin Med (2018) 7(12), 501. doi: 10.3390/jcm7120501 PMC6306931 30513772

[48] Dou S Zhang L Wang C Yao Y Jiang W Ye L. And 11q13 amplification are potential predictive biomarkers for immunotherapy in head and neck squamous cell carcinoma. Front Immunol (2022) 13:813732. doi: 10.3389/fimmu.2022.813732 35371031 PMC8965897

[49] Schulze K Imbeaud S Letouzé E Alexandrov LB Calderaro J Rebouissou S. Exome sequencing of hepatocellular carcinomas identifies new mutational signatures and potential therapeutic targets. Nat Genet (2015) 47:505–11. doi: 10.1038/ng.3252 PMC4587544 25822088

[50] Khemlina G Ikeda S Kurzrock R. The biology of hepatocellular carcinoma: Implications for genomic and immune therapies. Mol Cancer (2017) 16, 149. doi: 10.1186/s12943-017-0712-x PMC5577674 28854942

[51] Ricketts CJ De Cubas AA Fan H Smith CC Lang M Reznik E. The cancer genome atlas comprehensive molecular characterization of renal cell carcinoma. Cell Rep (2018) 23:313–326.e315. doi: 10.1016/j.celrep.2018.03.075 29617669 PMC6075733

[52] Yi C Chen L Lin Z Liu L Shao W Zhang R. Lenvatinib targets FGF receptor 4 to enhance antitumor immune response of anti-programmed cell death-1 in HCC. Hepatology (2021) 74:2544–60. doi: 10.1002/hep.31921 34036623

[53] Zhang W Gong C Peng X Bi X Sun Y Zhou J. Serum concentration of CD137 and tumor infiltration by M1 macrophages predict the response to sintilimab plus bevacizumab biosimilar in advanced hepatocellular carcinoma patients. Clin Cancer Res (2022) 28:3499–508. doi: 10.1158/1078-0432.CCR-21-3972 PMC9662860 35275208

[54] Kamada T Togashi Y Tay C Ha D Sasaki A Nakamura Y. PD-1+ regulatory T cells amplified by PD-1 blockade promote hyperprogression of cancer. Proc Natl Acad Sci (2019) 116:9999–10008. doi: 10.1073/pnas.1822001116 31028147 PMC6525547

[55] Nicholes K Guillet S Tomlinson E Hillan K Wright B Frantz GD. A mouse model of hepatocellular carcinoma: Ectopic expression of fibroblast growth factor 19 in skeletal muscle of transgenic mice. Am J Pathol (2002) 160:2295–307. doi: 10.1016/S0002-9440(10)61177-7 PMC1850847 12057932

[56] Sawey ET Chanrion M Cai C Wu G Zhang J Zender L. Identification of a therapeutic strategy targeting amplified FGF19 in liver cancer by oncogenomic screening. Cancer Cell (2011) 19:347–58. doi: 10.1016/j.ccr.2011.01.040 PMC3061399 21397858

[57] Kim RD Sarker D Meyer T Yau T Macarulla T Park J-W. First-in-Human phase I study of fisogatinib (BLU-554) validates aberrant FGF19 signaling as a driver event in hepatocellular carcinoma. Cancer Discovery (2019) 9:1696–707. doi: 10.1158/2159-8290.CD-19-0555 31575541

[58] Arao T Ueshima K Matsumoto K Nagai T Kimura H Hagiwara S. FGF3/FGF4 amplification and multiple lung metastases in responders to sorafenib in hepatocellular carcinoma. Hepatology (2013) 57:1407–15. doi: 10.1002/hep.25956 22890726

[59] verÁlvarez-Fernández M Malumbres M. Mechanisms of sensitivity and resistance to CDK4/6 inhibition. Cancer Cell (2020) 37:514–29. doi: 10.1016/j.ccell.2020.03.010 32289274

[60] Singavi AK Menon S Kilari D Alqwasmi A Ritch PS Thomas JP. 1140PD - predictive biomarkers for hyper-progression (HP) in response to immune checkpoint inhibitors (ICI) – analysis of somatic alterations (SAs). Ann Oncol (2017) 28:v405. doi: 10.1093/annonc/mdx376.006

[61] Kato S Goodman A Walavalkar V Barkauskas DA Sharabi A Kurzrock R. Hyperprogressors after immunotherapy: Analysis of genomic alterations associated with accelerated growth rate. Clin Cancer Res (2017) 23:4242–50. doi: 10.1158/1078-0432.CCR-16-3133 PMC5647162 28351930

[62] Giusti R Mazzotta M Filetti M Marinelli D Di Napoli A Scarpino S. CDKN2A/B gene loss and MDM2 alteration as a potential molecular signature for hyperprogressive disease in advanced NSCLC: A next-generation-sequencing approach. J Clin Oncol (2019) 37:e20628–8. doi: 10.1200/JCO.2019.37.15_suppl.e20628

[63] Wong DJ Lee J Choo SP Thng CH Hennedige T. Hyperprogressive disease in hepatocellular carcinoma with immune checkpoint inhibitor use: A case series. Immunotherapy (2019) 11:167–75. doi: 10.2217/imt-2018-0126 30730278

[64] Choi W-M Kim JY Choi J Lee D Shim JH Lim Y-S. Kinetics of the neutrophil-lymphocyte ratio during PD-1 inhibition as a prognostic factor in advanced hepatocellular carcinoma. Liver Int (2021) 41:2189–99. doi: 10.1111/liv.14932 33966338

[65] Kim CG Kim C Yoon SE Kim KH Choi SJ Kang B. Hyperprogressive disease during PD-1 blockade in patients with advanced hepatocellular carcinoma. J Hepatol (2021) 74:350–9. doi: 10.1016/j.jhep.2020.08.010 32810553

[66] Zhang L Wu L Chen Q Zhang B Liu J Liu S. Predicting hyperprogressive disease in patients with advanced hepatocellular carcinoma treated with anti-programmed cell death 1 therapy. EClinicalMedicine (2021) 31:100673. doi: 10.1016/j.eclinm.2020.100673 33554079 PMC7846667

