# Supplementary material for: Chromosome 11q13 amplification correlates with poor response and prognosis to PD-1 blockade in unresectable hepatocellular carcinoma
Source: Front Immunol. 2023 Mar 28;14:1116057. doi: 10.3389/fimmu.2023.1116057 (PMC10086239; doi:10.3389/fimmu.2023.1116057)
Supplement: Supplementary Figure 1 — Summary of frequently (Top 20) genomic characterized copy number alterations among 62 patients with HCC. [file DataSheet_1.zip › coming fig 5.pptx]

## Slide 1
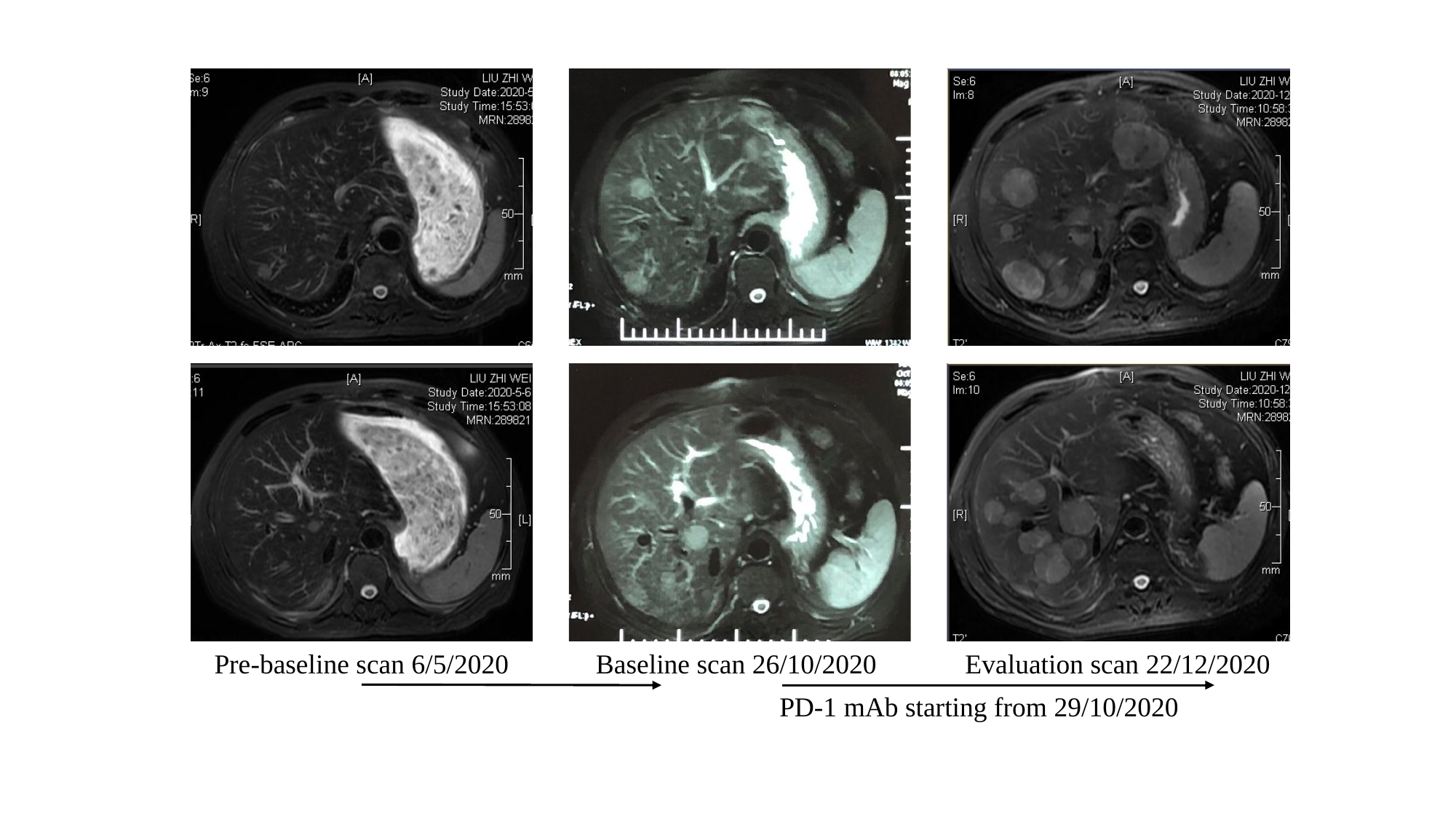

Pre-baseline scan 6/5/2020
Baseline scan 26/10/2020
Evaluation scan 22/12/2020
PD-1 mAb starting from 29/10/2020

## Slide 2
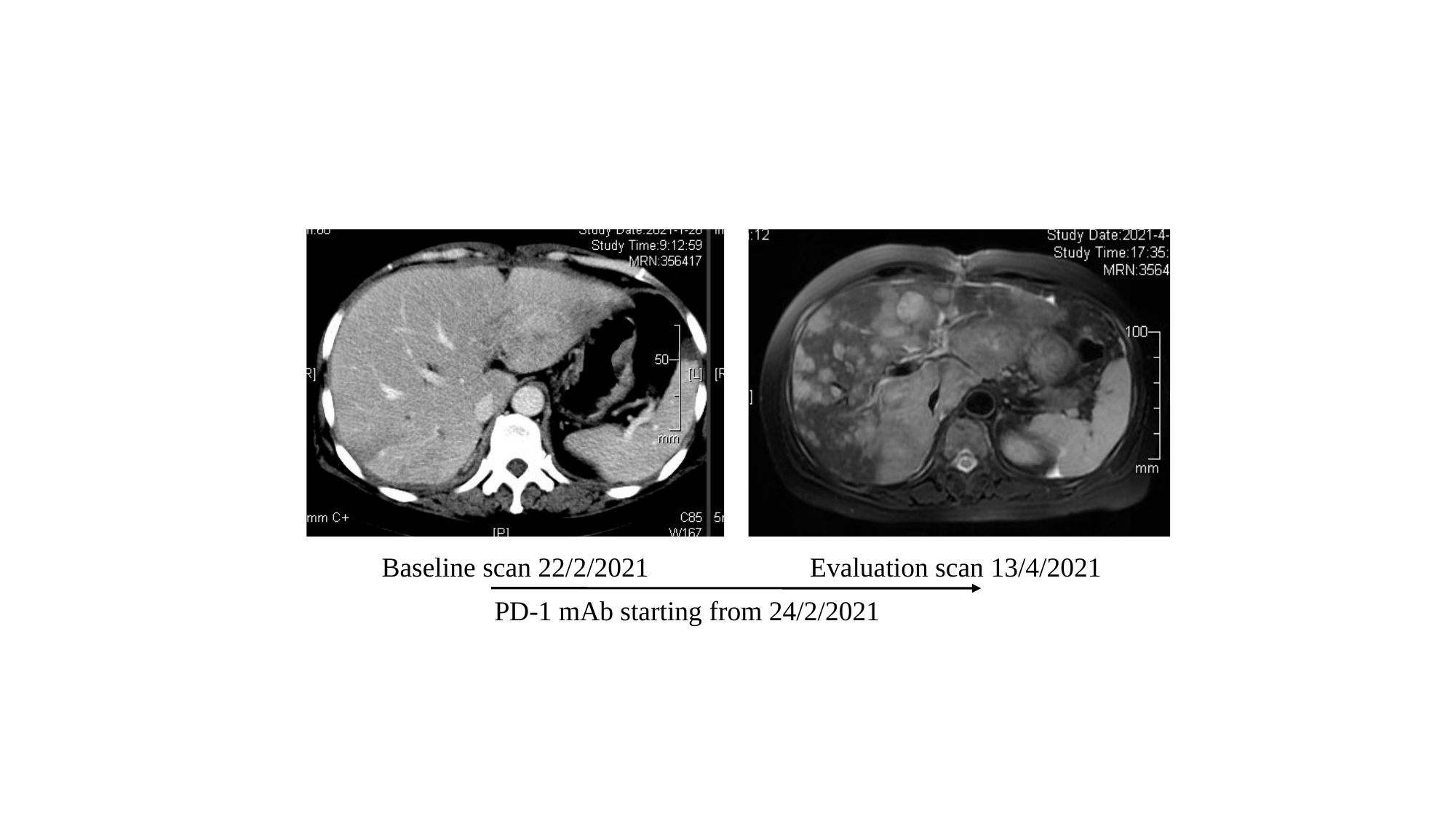

Baseline scan 22/2/2021
Evaluation scan 13/4/2021
PD-1 mAb starting from 24/2/2021

## Slide 3
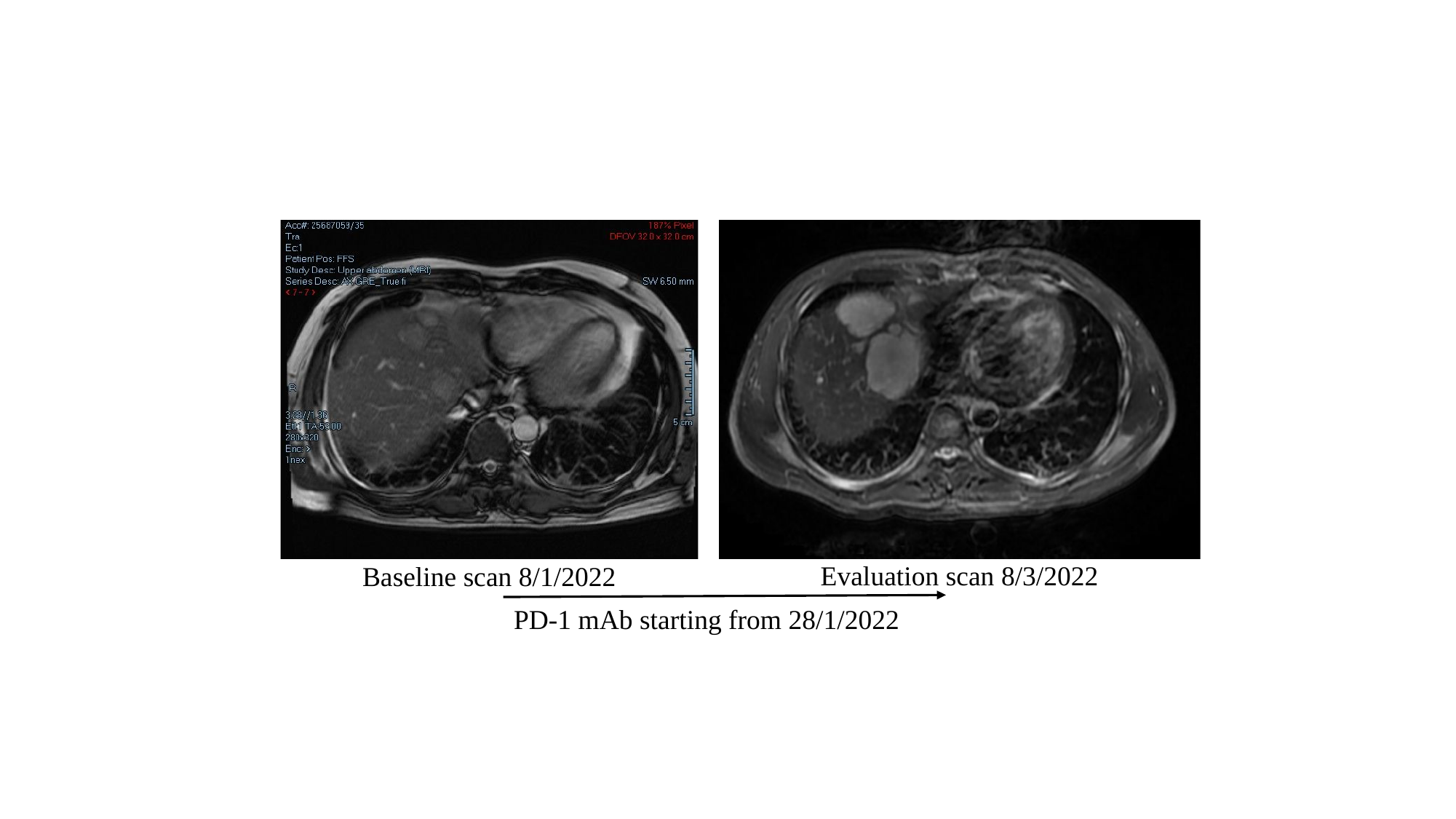

Evaluation scan 8/3/2022
Baseline scan 8/1/2022
PD-1 mAb starting from 28/1/2022
