# Supplementary figures and images for: Chromosome 11q13 amplification correlates with poor response and prognosis to PD-1 blockade in unresectable hepatocellular carcinoma
Source: Front Immunol. 2023 Mar 28;14:1116057. doi: 10.3389/fimmu.2023.1116057 (PMC10086239; doi:10.3389/fimmu.2023.1116057)

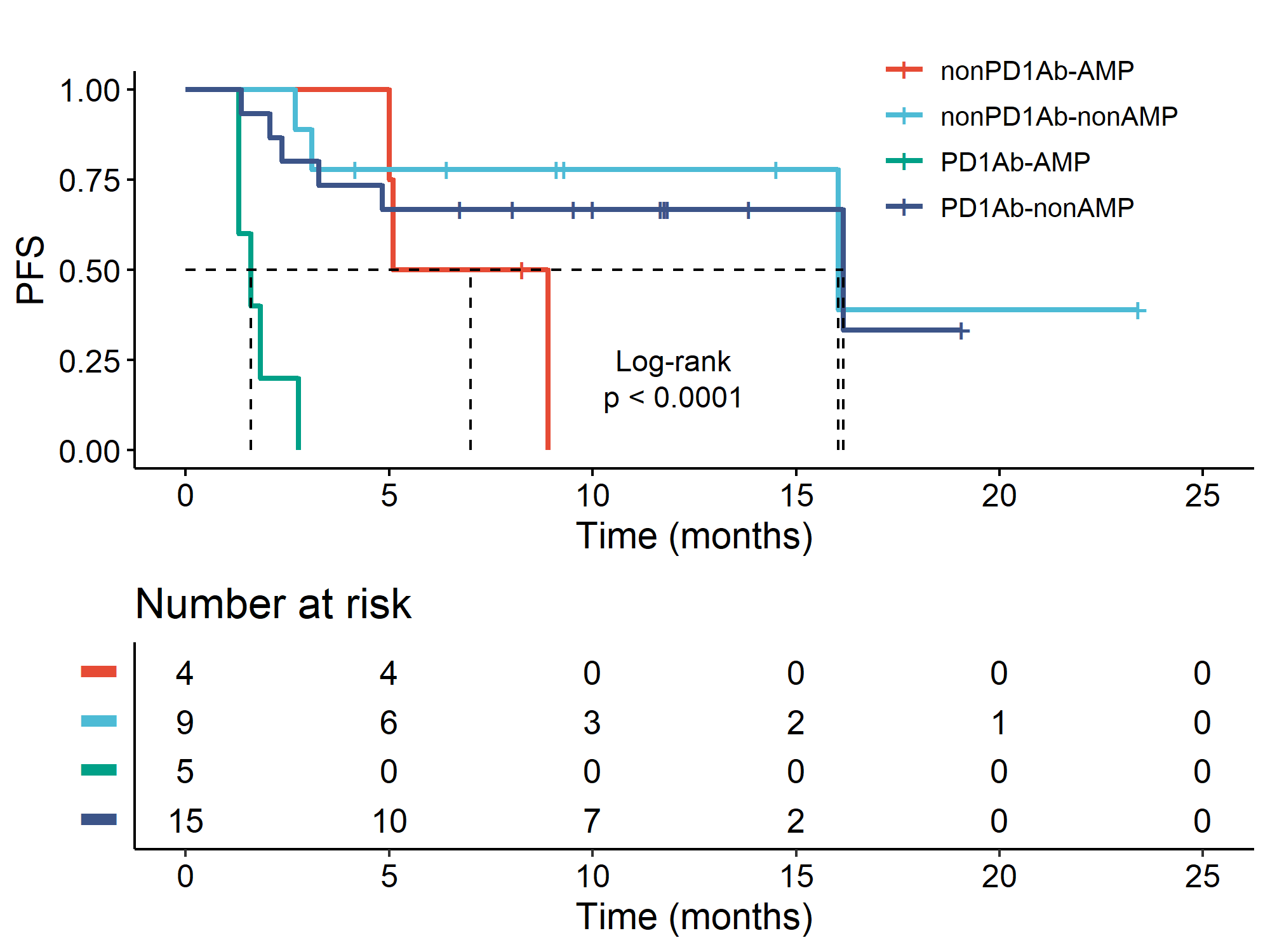

Supplement: Supplementary Figure 1 — Summary of frequently (Top 20) genomic characterized copy number alterations among 62 patients with HCC. [file DataSheet_1.zip › clinical/Fig 3D.tiff]

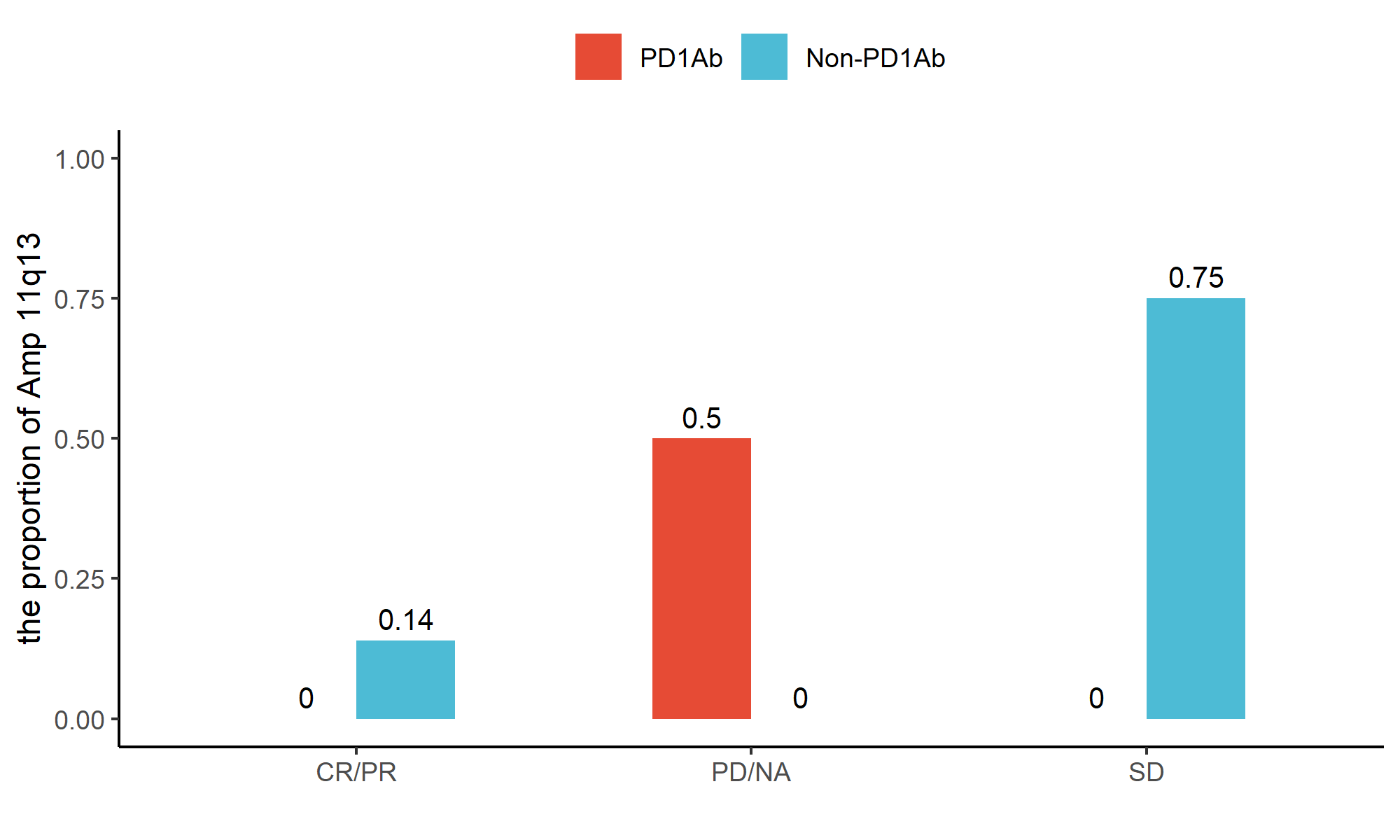

Supplement: Supplementary Figure 1 — Summary of frequently (Top 20) genomic characterized copy number alterations among 62 patients with HCC. [file DataSheet_1.zip › clinical/Fig3_amp_proportion_20220821.tiff]

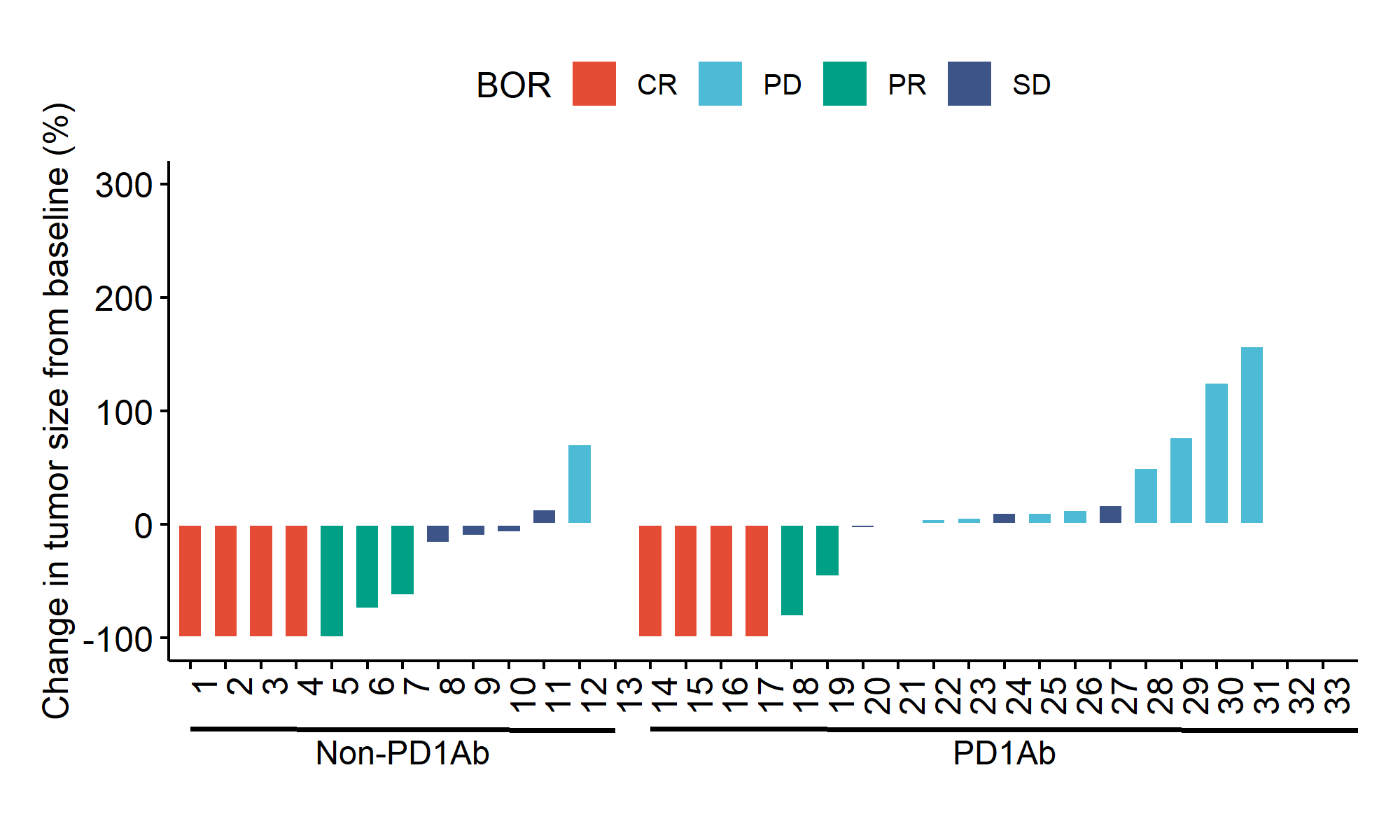

Supplement: Supplementary Figure 1 — Summary of frequently (Top 20) genomic characterized copy number alterations among 62 patients with HCC. [file DataSheet_1.zip › clinical/Fig3_flow_20220821.tiff]

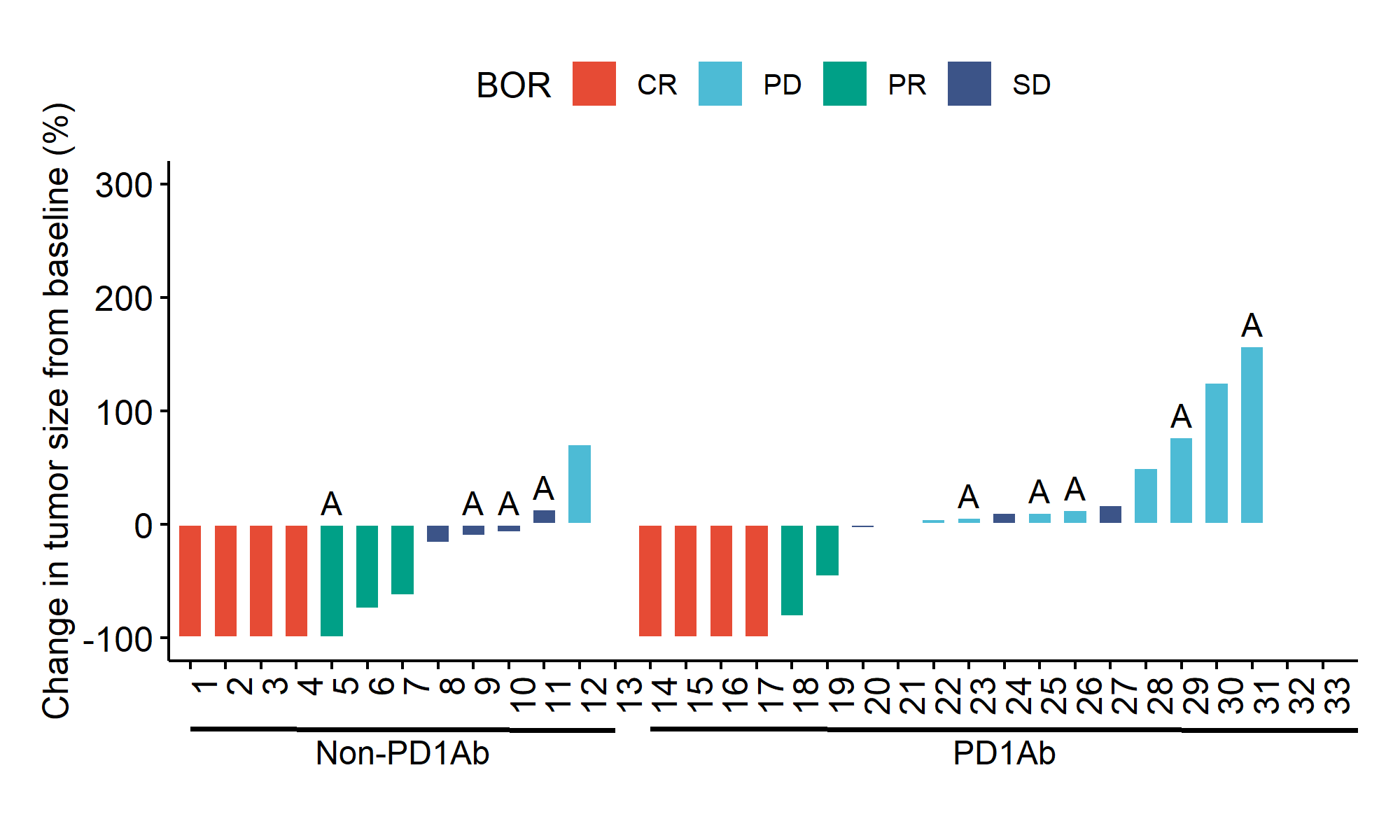

Supplement: Supplementary Figure 1 — Summary of frequently (Top 20) genomic characterized copy number alterations among 62 patients with HCC. [file DataSheet_1.zip › clinical/Fig3_flow_20220921.tiff]

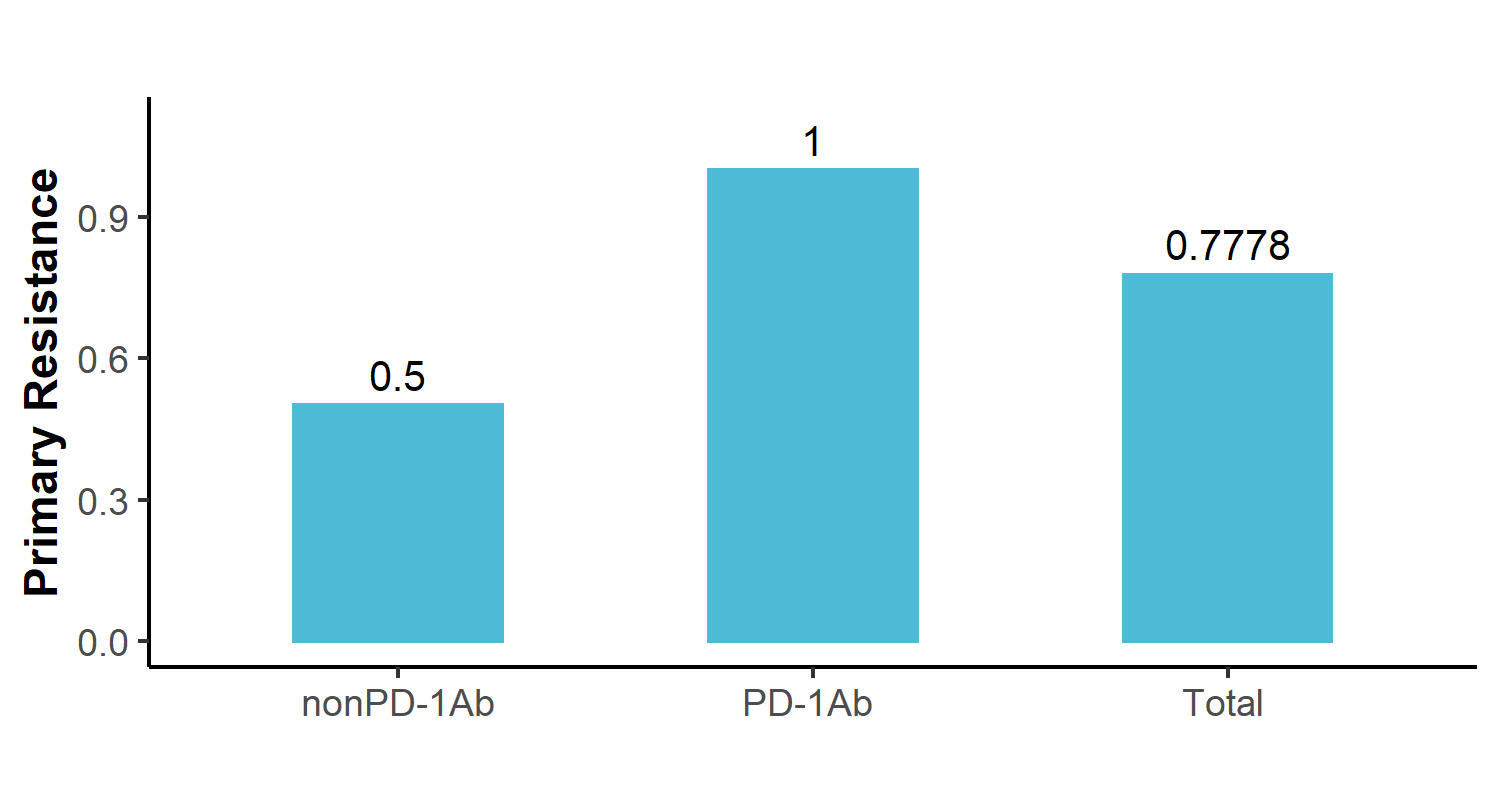

Supplement: Supplementary Figure 1 — Summary of frequently (Top 20) genomic characterized copy number alterations among 62 patients with HCC. [file DataSheet_1.zip › clinical/Fig3_Primary_Resistance_proportion_20220909.tiff]

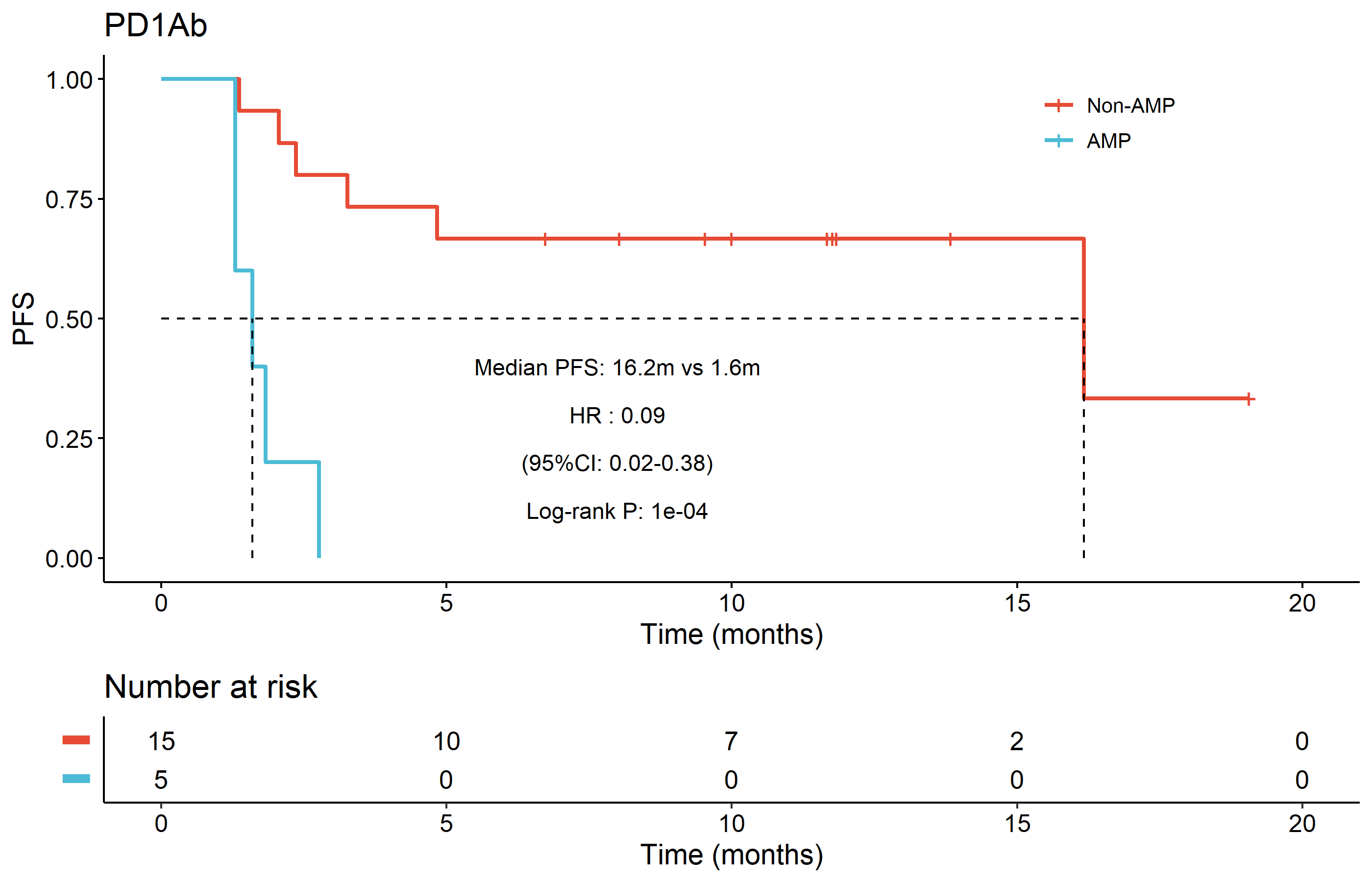

Supplement: Supplementary Figure 1 — Summary of frequently (Top 20) genomic characterized copy number alterations among 62 patients with HCC. [file DataSheet_1.zip › clinical/Fig4A1.tiff]

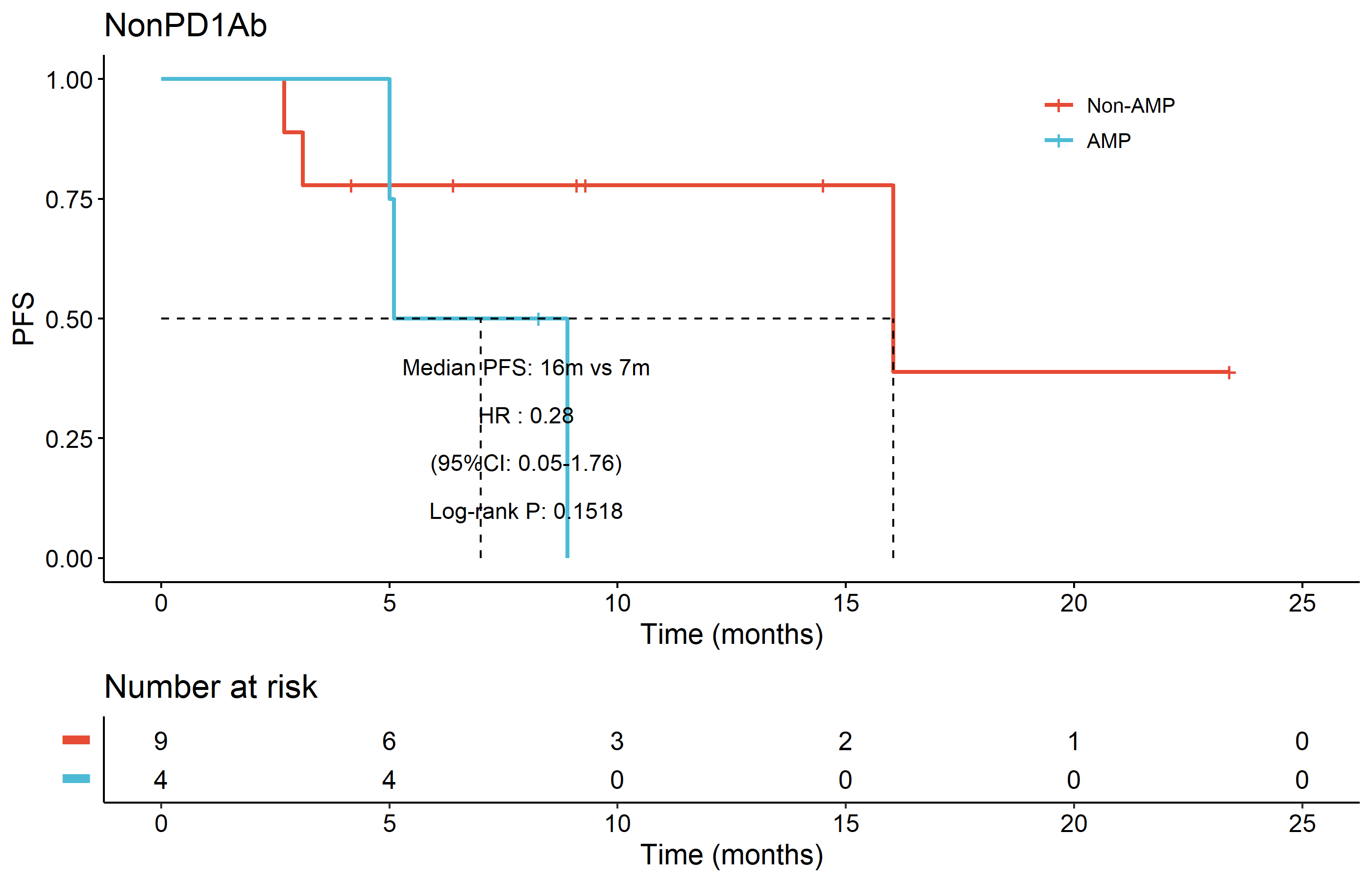

Supplement: Supplementary Figure 1 — Summary of frequently (Top 20) genomic characterized copy number alterations among 62 patients with HCC. [file DataSheet_1.zip › clinical/Fig4A2.tiff]

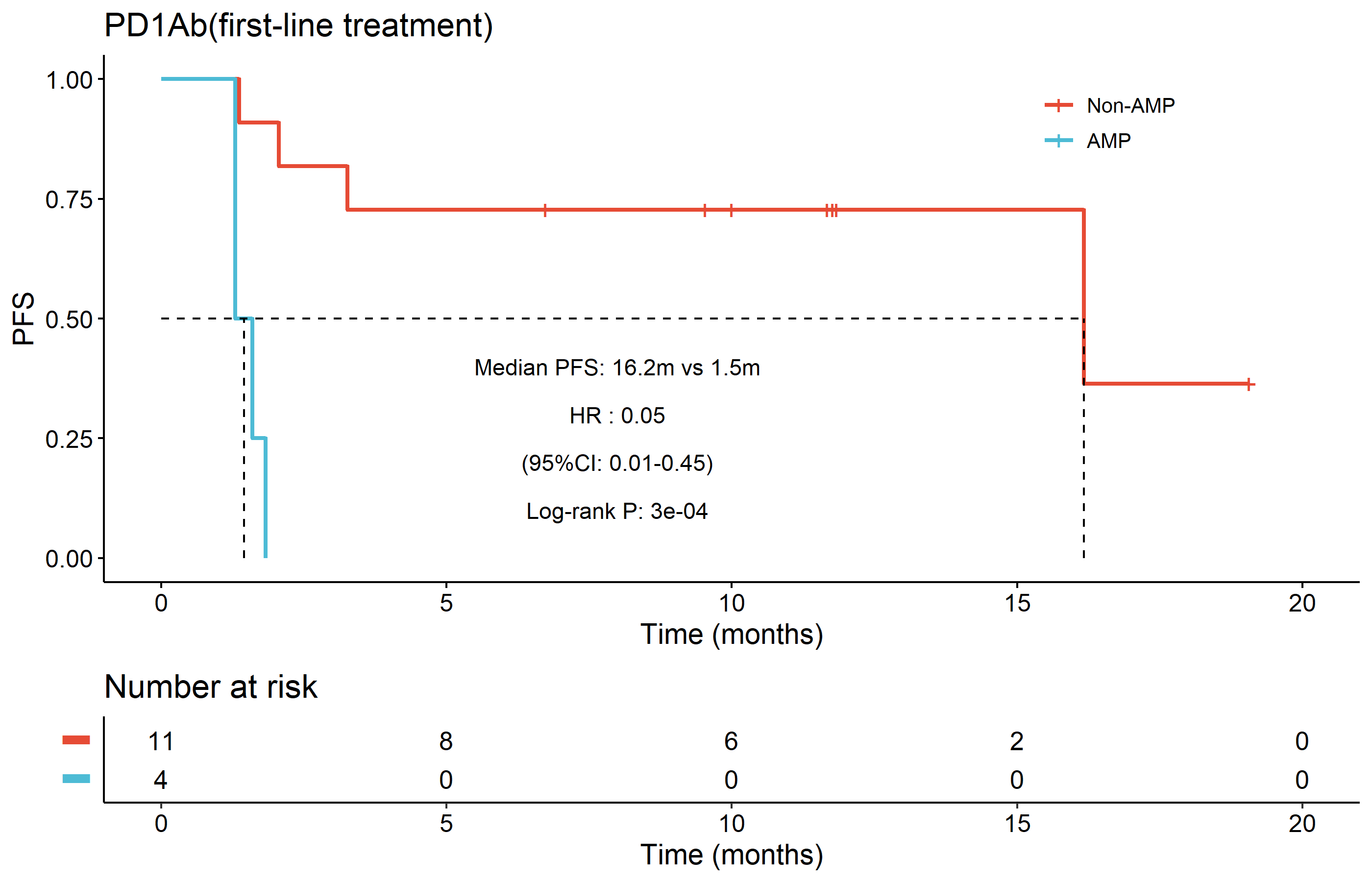

Supplement: Supplementary Figure 1 — Summary of frequently (Top 20) genomic characterized copy number alterations among 62 patients with HCC. [file DataSheet_1.zip › clinical/Fig4B1.tiff]

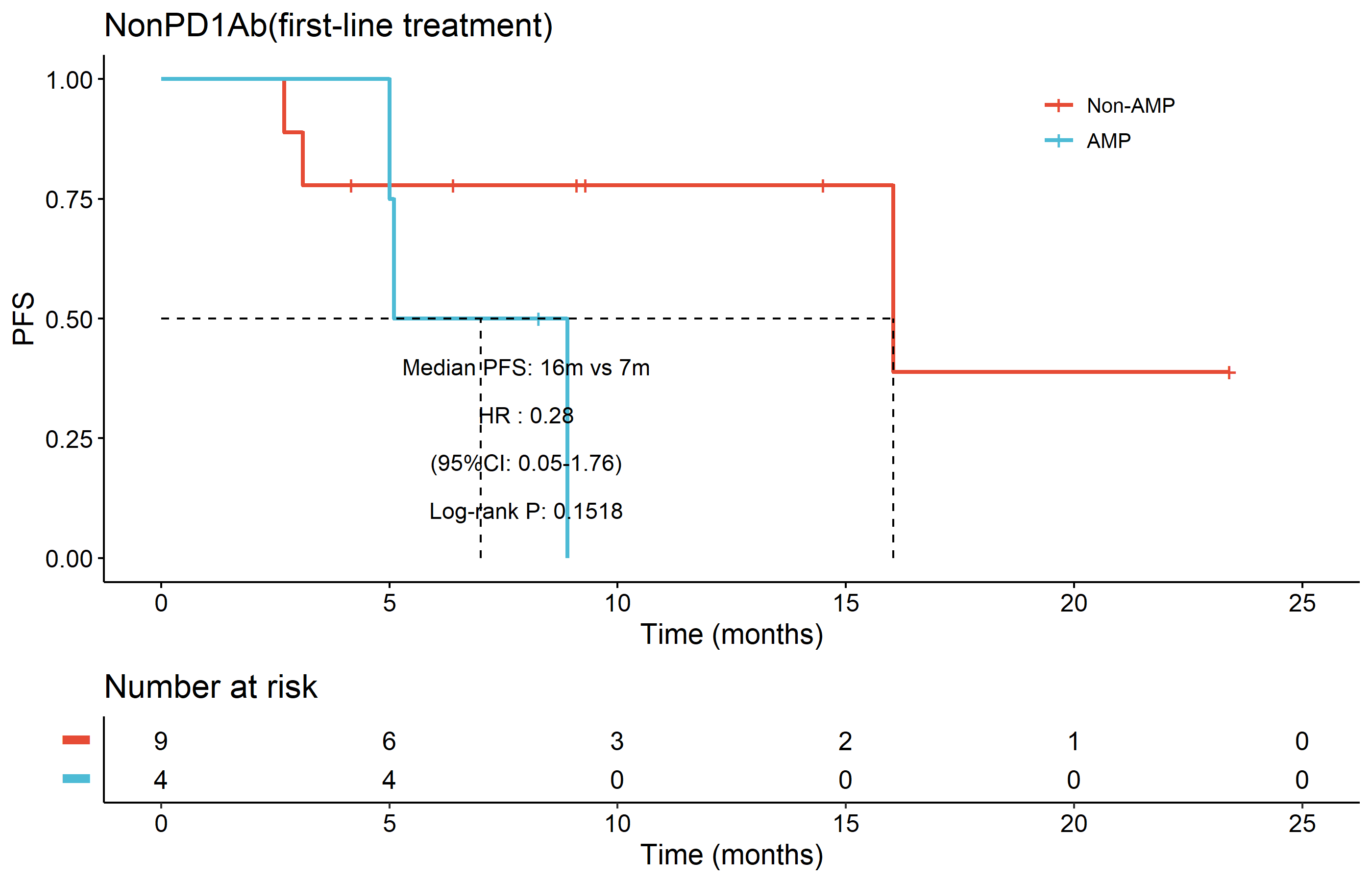

Supplement: Supplementary Figure 1 — Summary of frequently (Top 20) genomic characterized copy number alterations among 62 patients with HCC. [file DataSheet_1.zip › clinical/Fig4B2.tiff]

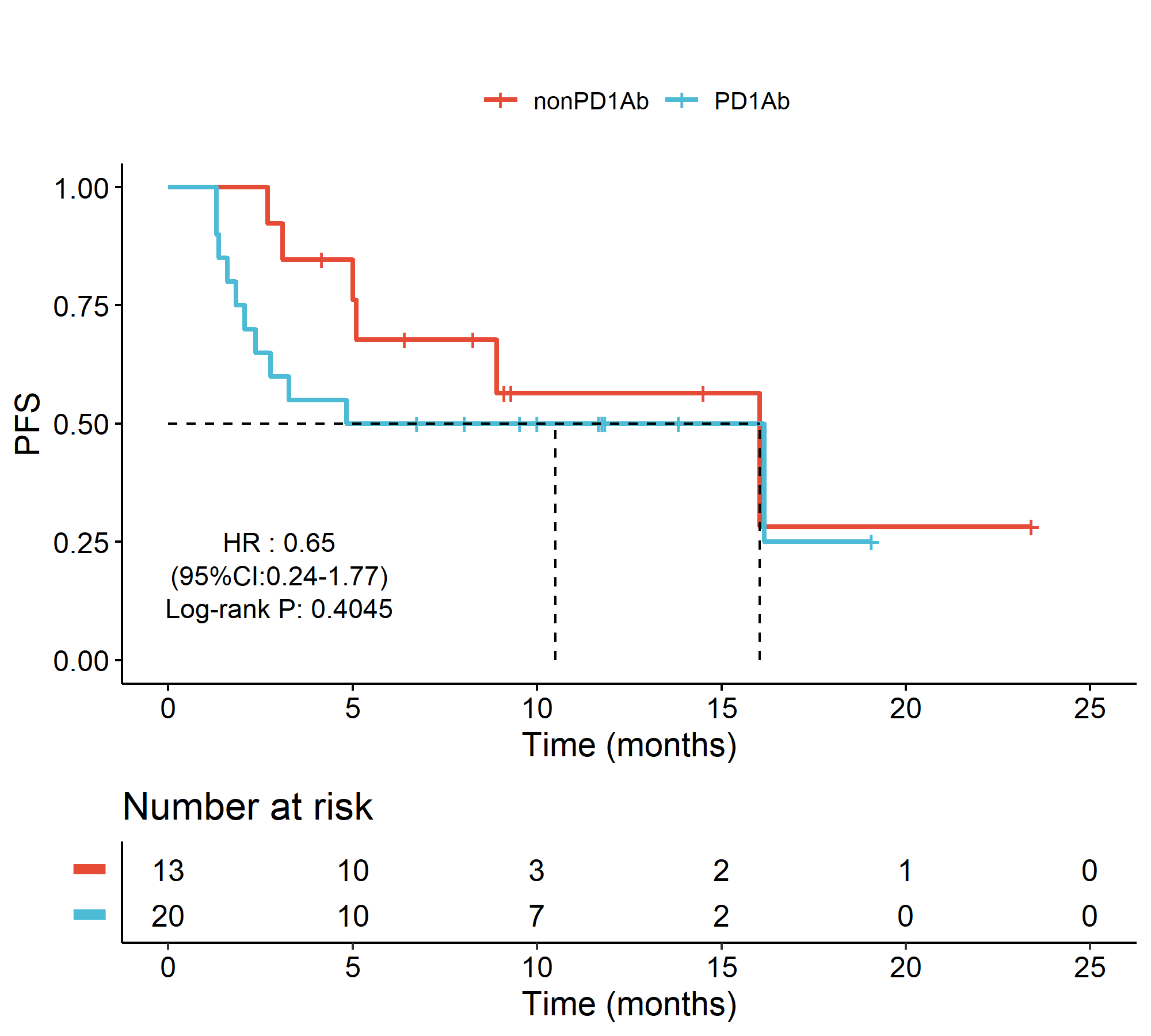

Supplement: Supplementary Figure 1 — Summary of frequently (Top 20) genomic characterized copy number alterations among 62 patients with HCC. [file DataSheet_1.zip › clinical/FigS4.tiff]

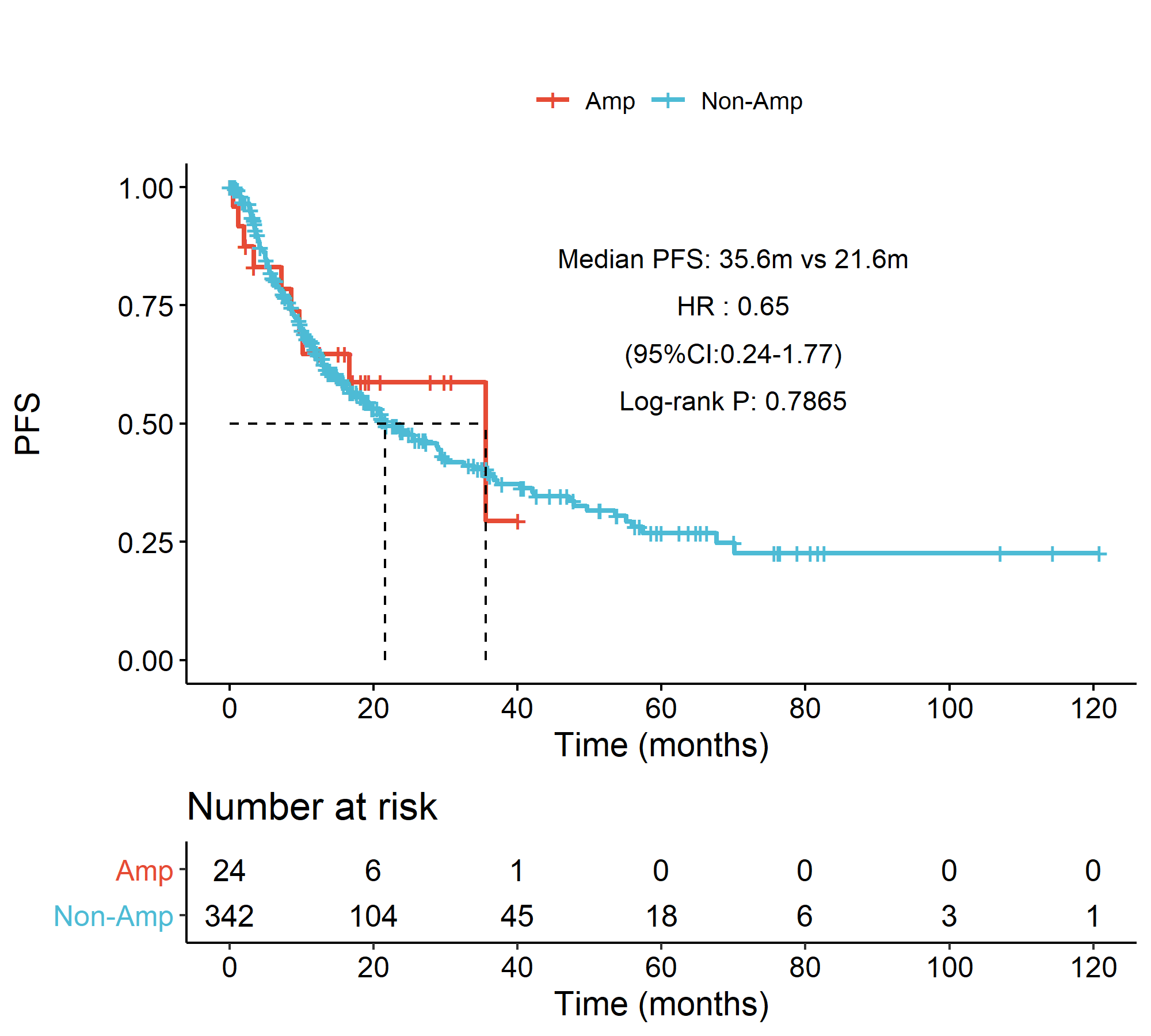

Supplement: Supplementary Figure 1 — Summary of frequently (Top 20) genomic characterized copy number alterations among 62 patients with HCC. [file DataSheet_1.zip › clinical/Supplementary Figure 3 _km_tcga_20220920.tiff]

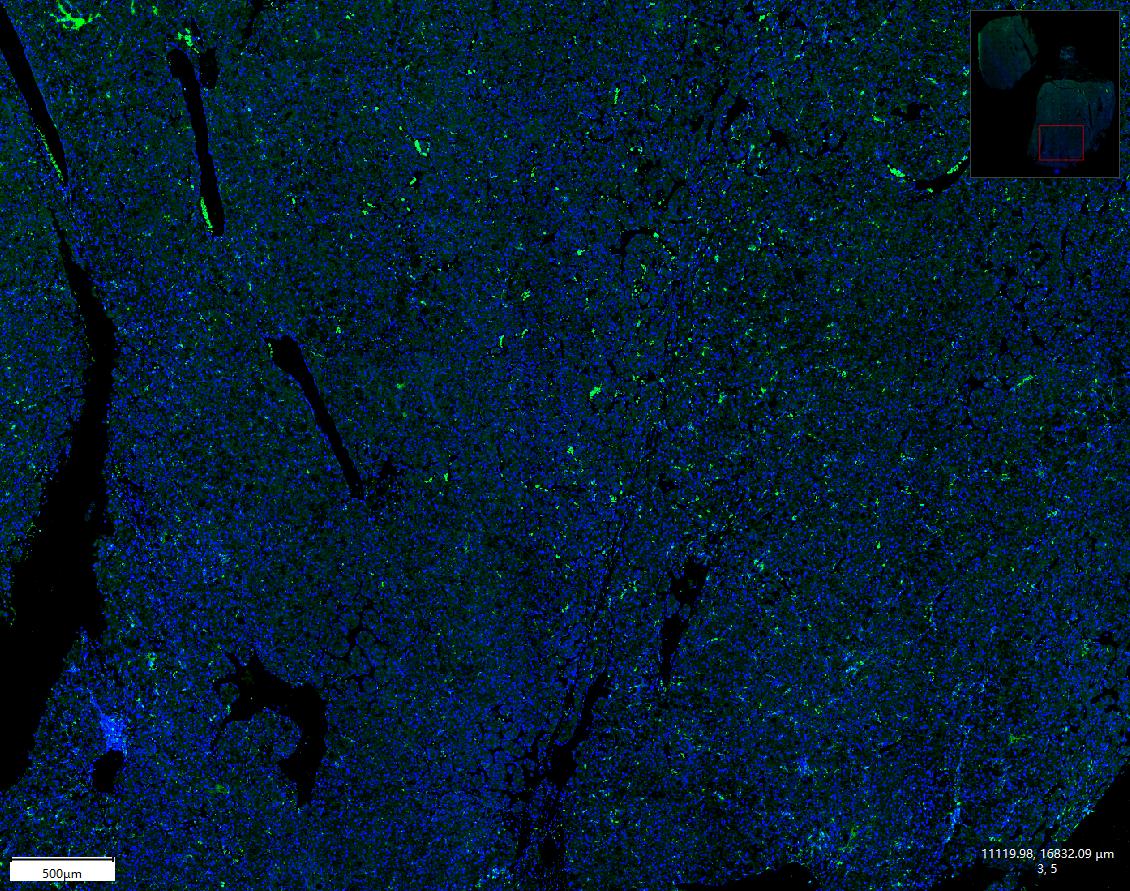

Supplement: Supplementary Figure 1 — Summary of frequently (Top 20) genomic characterized copy number alterations among 62 patients with HCC. [file DataSheet_1.zip › Immune_Infiltration_Estimation/102585S01-P1_Scan1.jpg]

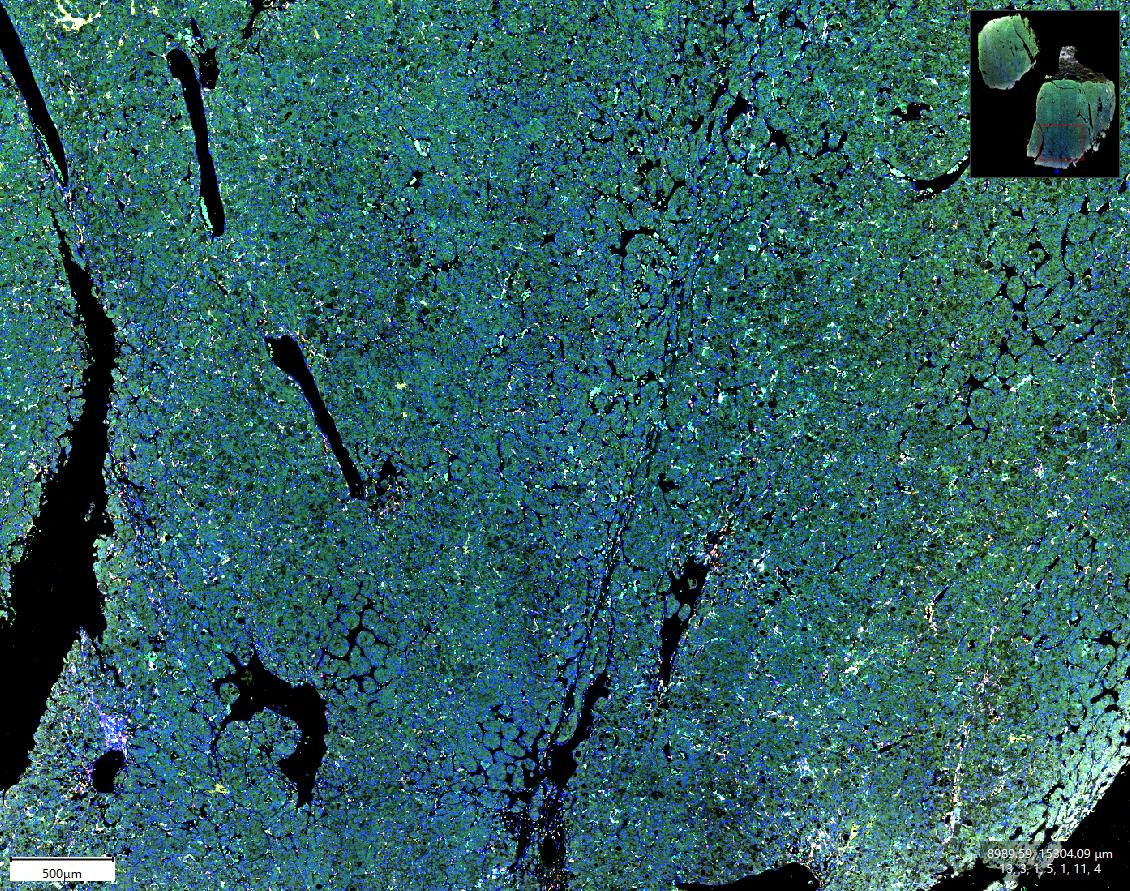

Supplement: Supplementary Figure 1 — Summary of frequently (Top 20) genomic characterized copy number alterations among 62 patients with HCC. [file DataSheet_1.zip › Immune_Infiltration_Estimation/102585S01-P1_Scan11.jpg]

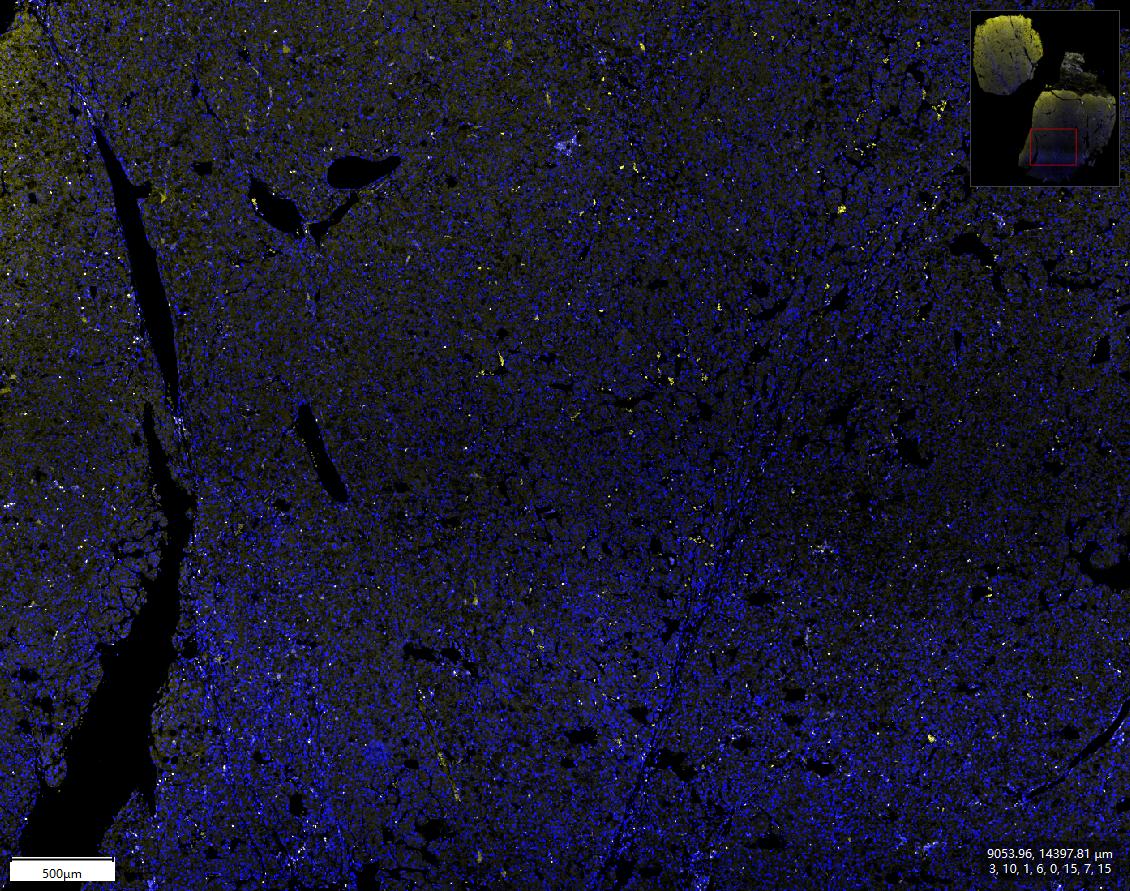

Supplement: Supplementary Figure 1 — Summary of frequently (Top 20) genomic characterized copy number alterations among 62 patients with HCC. [file DataSheet_1.zip › Immune_Infiltration_Estimation/102585S01-P2_Scan1.jpg]

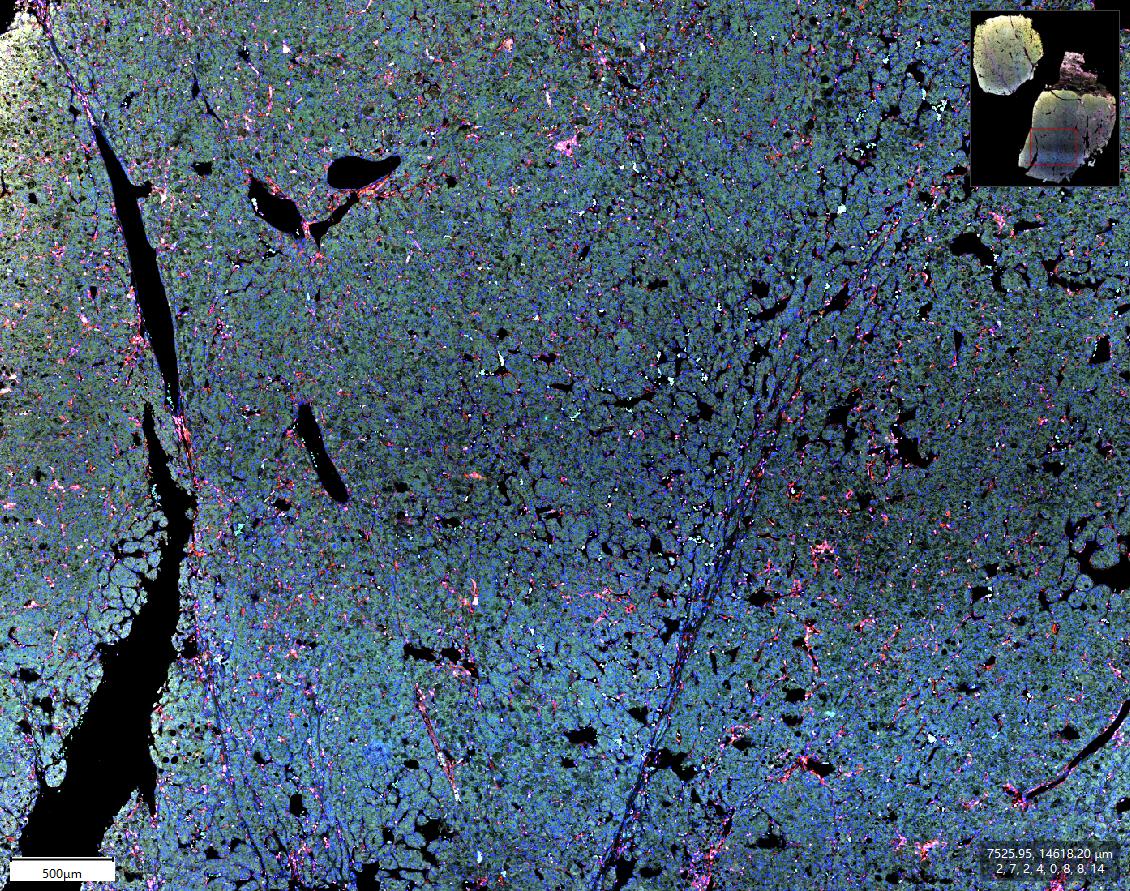

Supplement: Supplementary Figure 1 — Summary of frequently (Top 20) genomic characterized copy number alterations among 62 patients with HCC. [file DataSheet_1.zip › Immune_Infiltration_Estimation/102585S01-P2_Scan11.jpg]

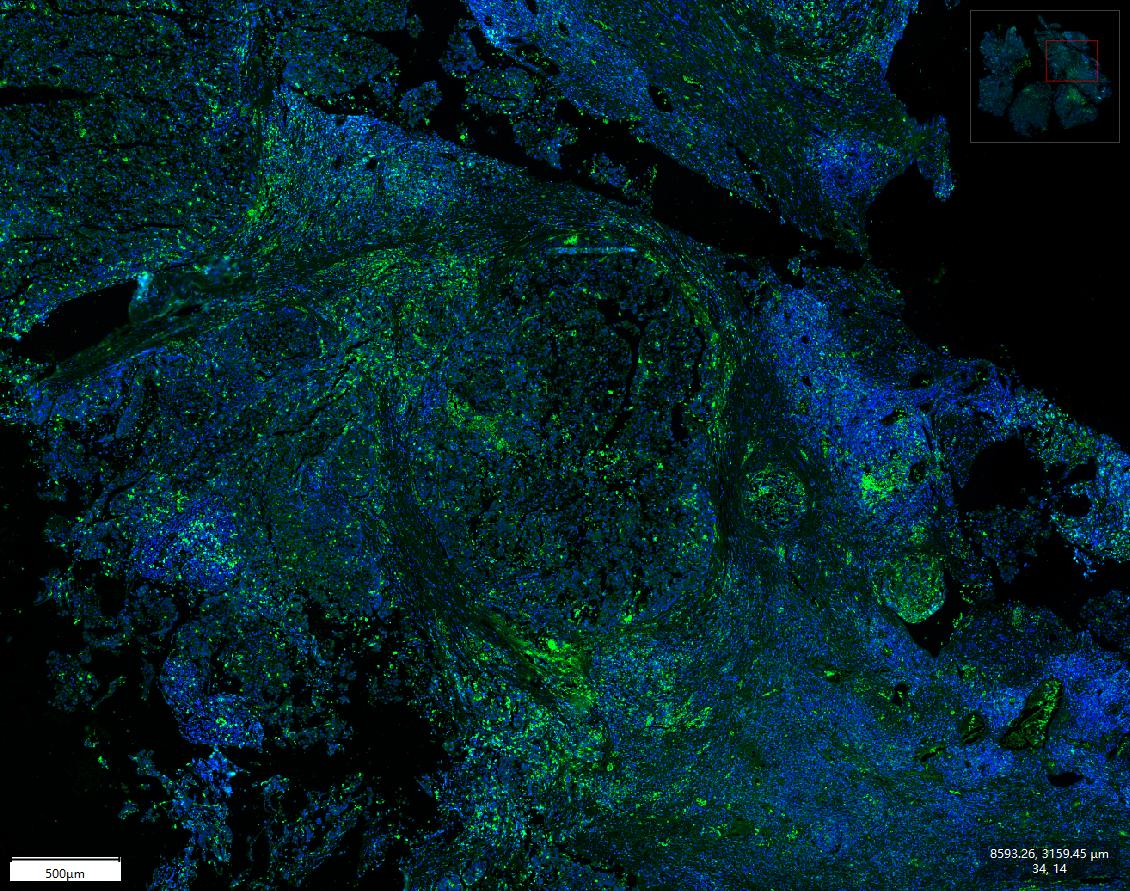

Supplement: Supplementary Figure 1 — Summary of frequently (Top 20) genomic characterized copy number alterations among 62 patients with HCC. [file DataSheet_1.zip › Immune_Infiltration_Estimation/86066S01-P1_Scan1.jpg]

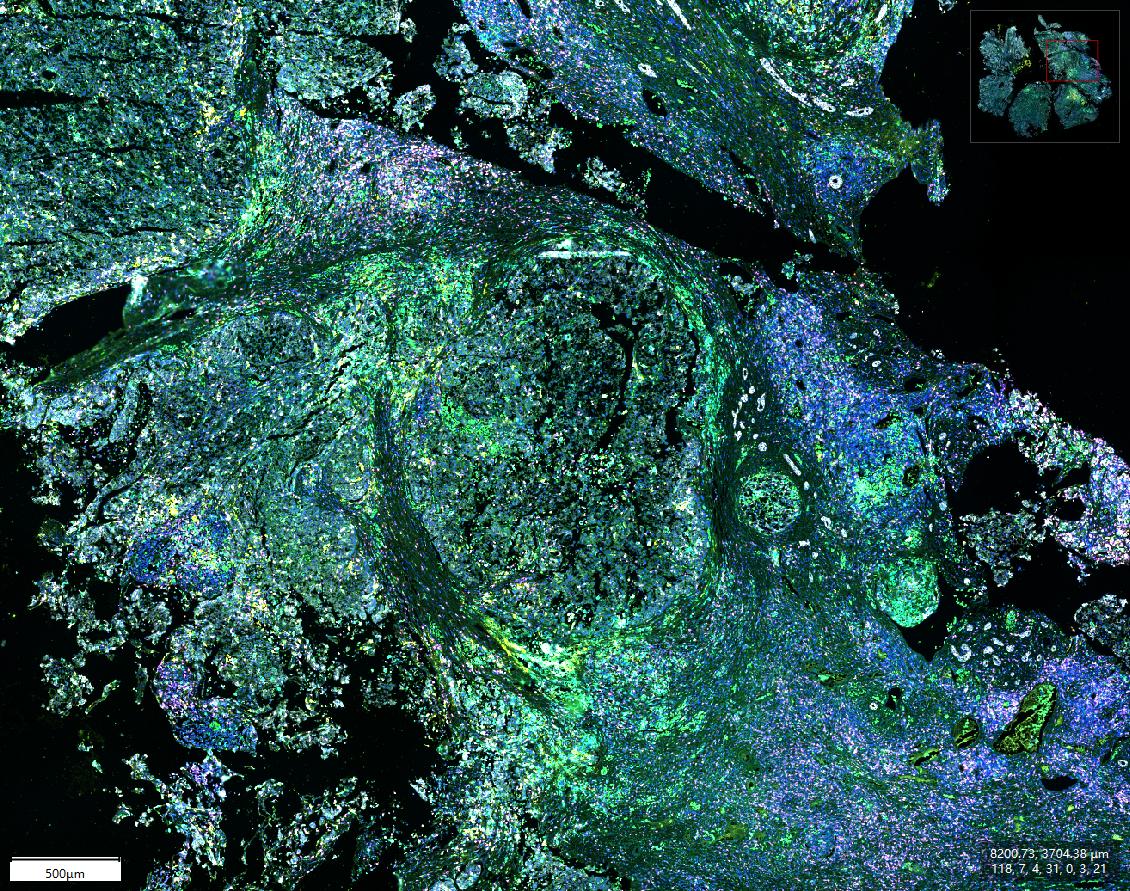

Supplement: Supplementary Figure 1 — Summary of frequently (Top 20) genomic characterized copy number alterations among 62 patients with HCC. [file DataSheet_1.zip › Immune_Infiltration_Estimation/86066S01-P1_Scan11.jpg]

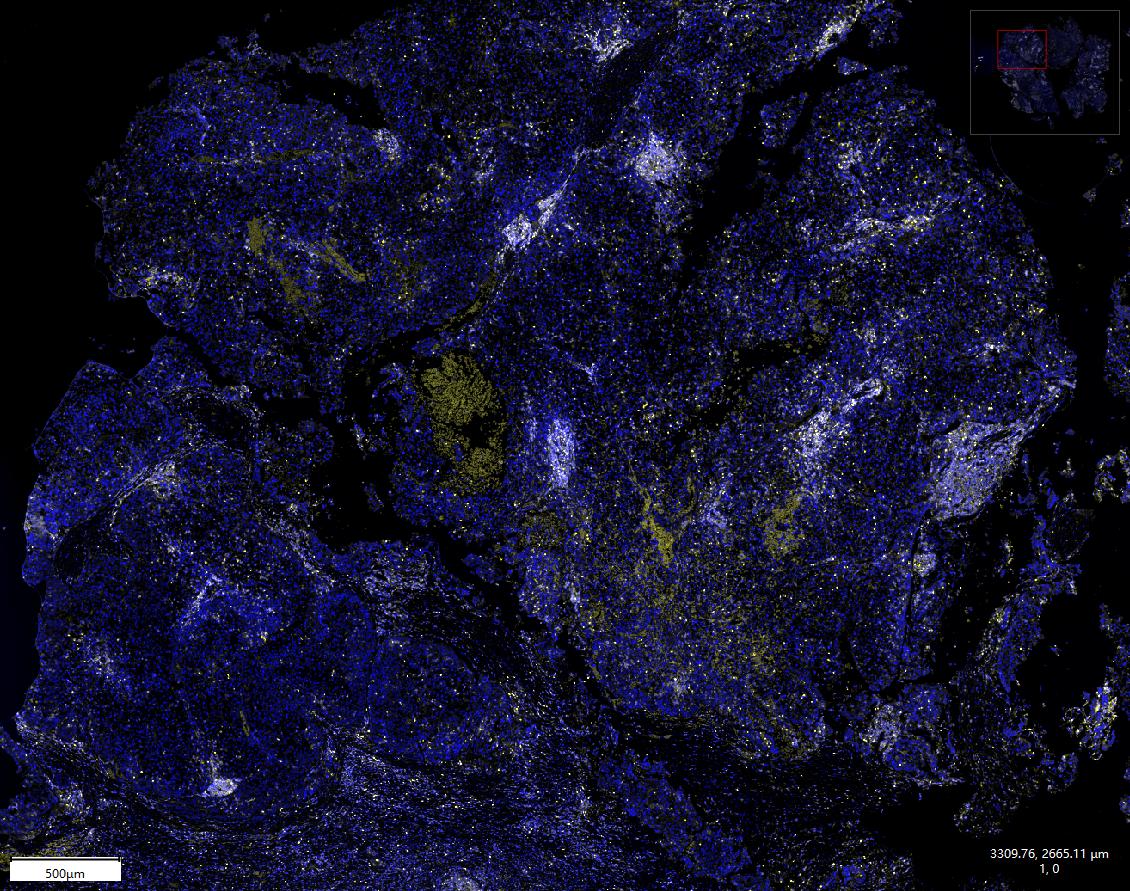

Supplement: Supplementary Figure 1 — Summary of frequently (Top 20) genomic characterized copy number alterations among 62 patients with HCC. [file DataSheet_1.zip › Immune_Infiltration_Estimation/86066S01-P2_Scan1.jpg]

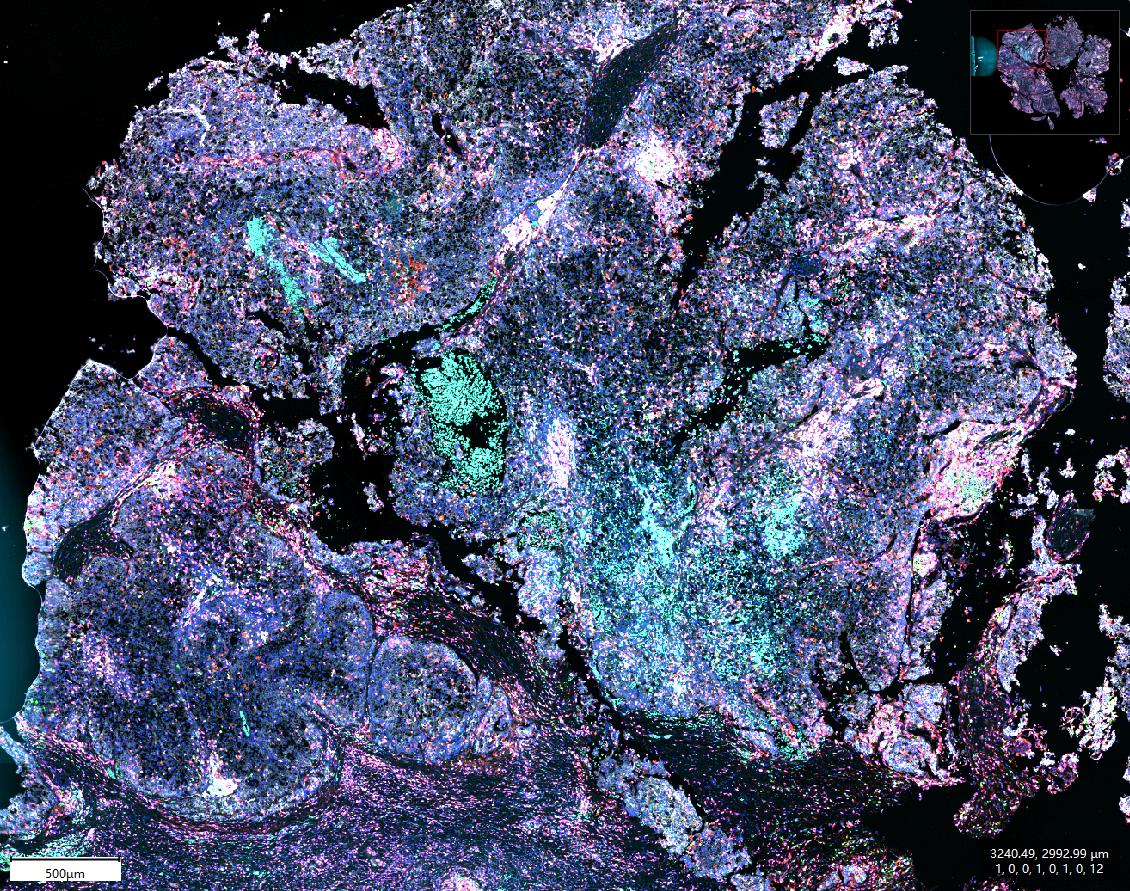

Supplement: Supplementary Figure 1 — Summary of frequently (Top 20) genomic characterized copy number alterations among 62 patients with HCC. [file DataSheet_1.zip › Immune_Infiltration_Estimation/86066S01-P2_Scan11.jpg]

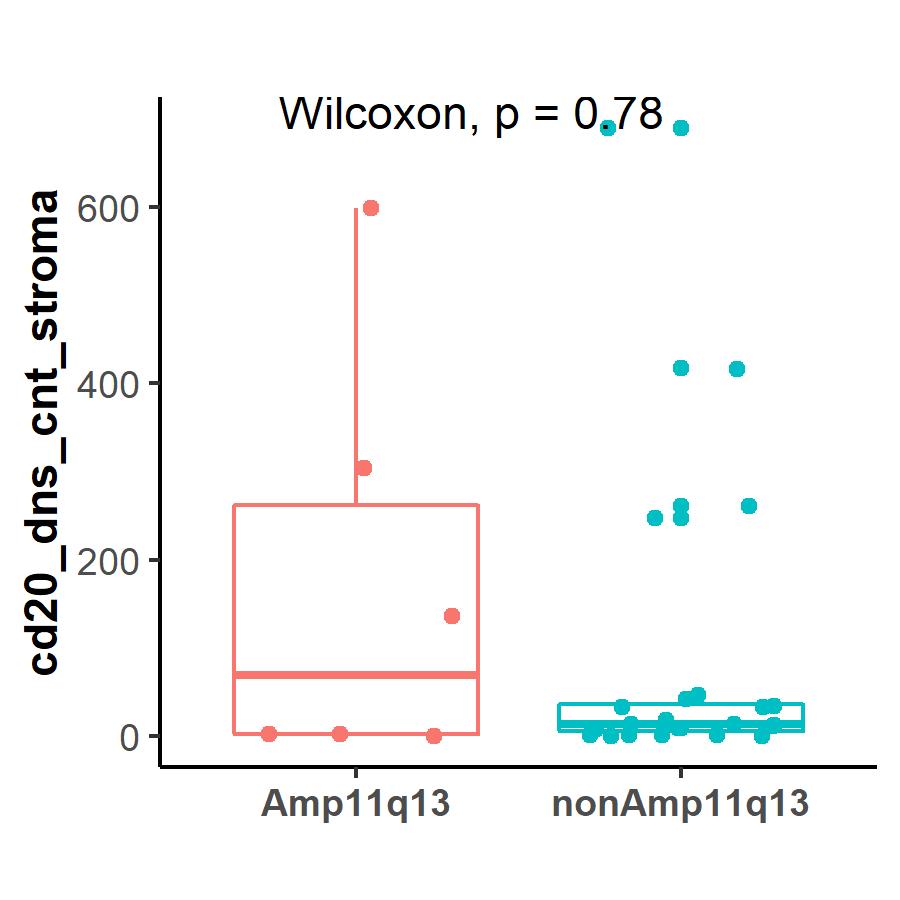

Supplement: Supplementary Figure 1 — Summary of frequently (Top 20) genomic characterized copy number alterations among 62 patients with HCC. [file DataSheet_1.zip › Immune_Infiltration_Estimation/cd20_dns_cnt_stroma_0.775_.jpeg]

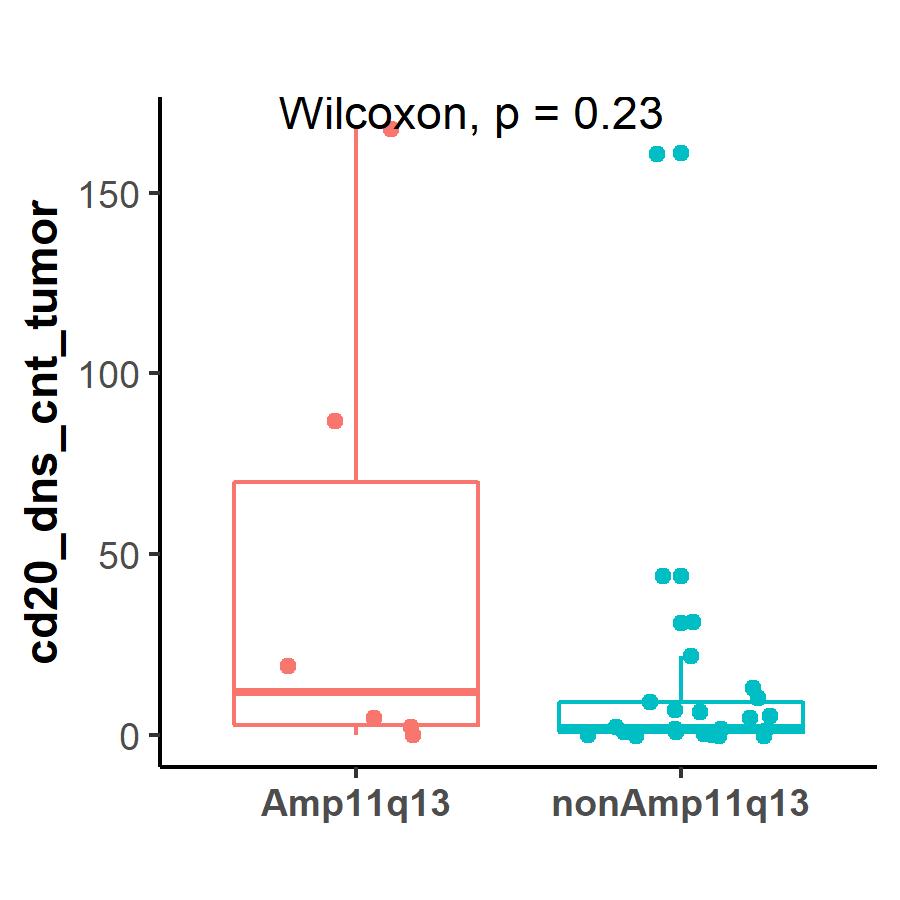

Supplement: Supplementary Figure 1 — Summary of frequently (Top 20) genomic characterized copy number alterations among 62 patients with HCC. [file DataSheet_1.zip › Immune_Infiltration_Estimation/cd20_dns_cnt_tumor_0.229_.jpeg]

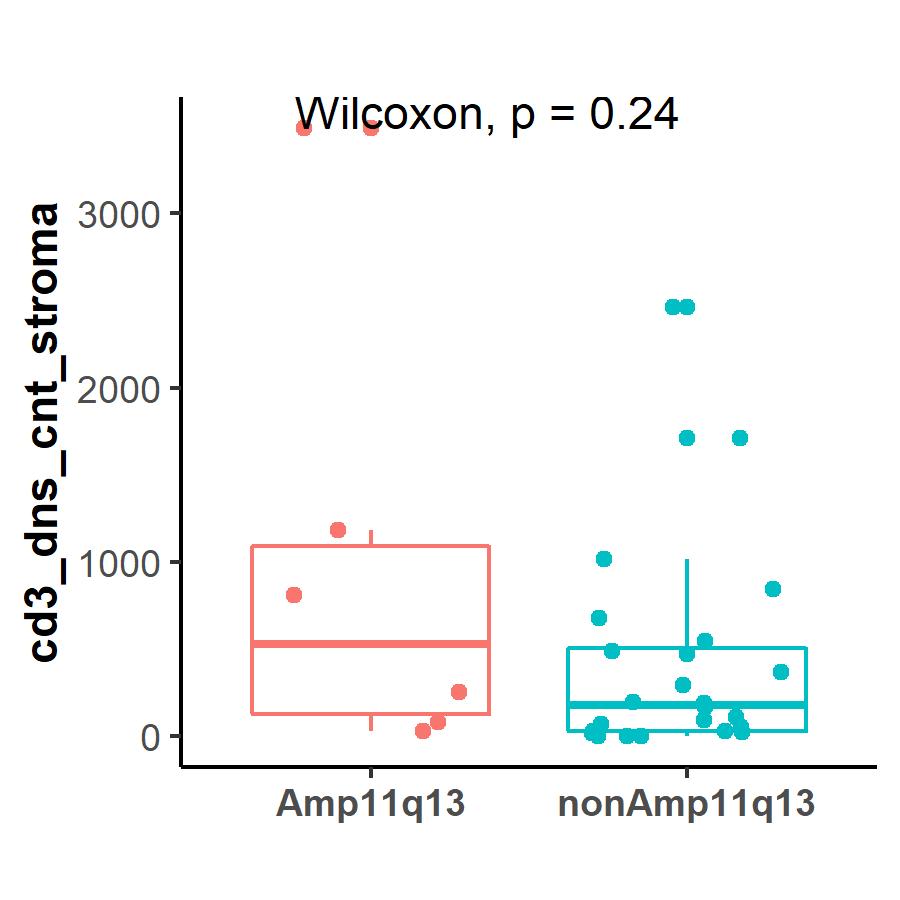

Supplement: Supplementary Figure 1 — Summary of frequently (Top 20) genomic characterized copy number alterations among 62 patients with HCC. [file DataSheet_1.zip › Immune_Infiltration_Estimation/cd3_dns_cnt_stroma_0.243_.jpeg]

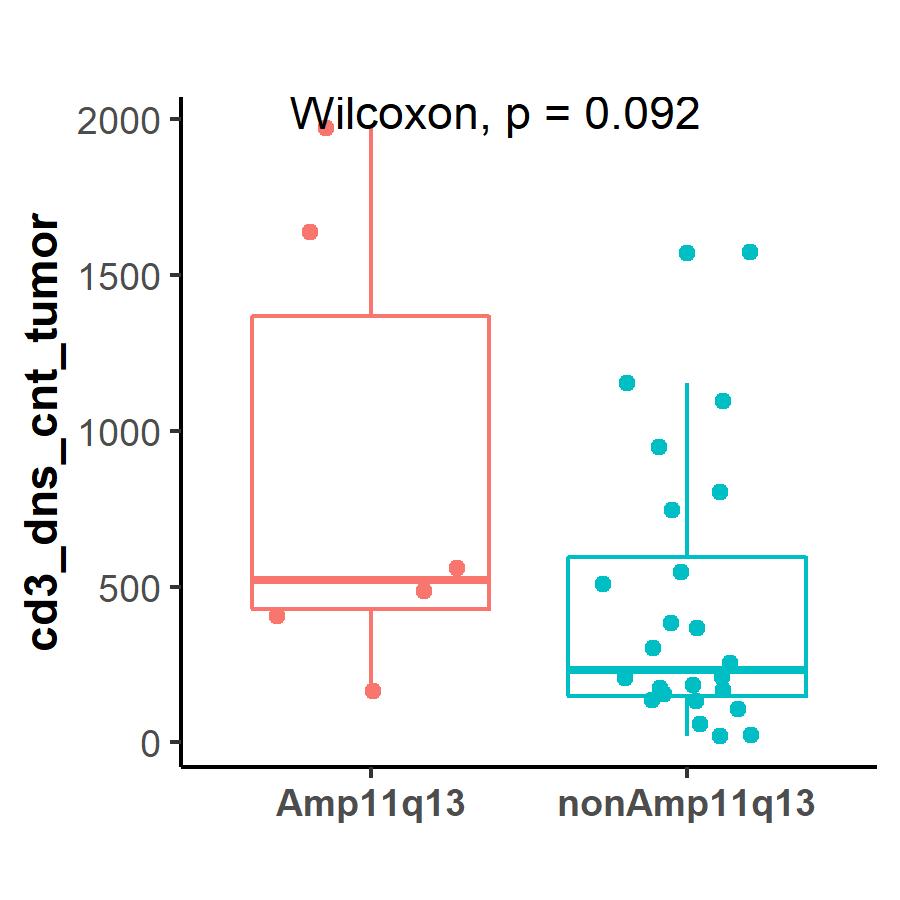

Supplement: Supplementary Figure 1 — Summary of frequently (Top 20) genomic characterized copy number alterations among 62 patients with HCC. [file DataSheet_1.zip › Immune_Infiltration_Estimation/cd3_dns_cnt_tumor_0.092_.jpeg]

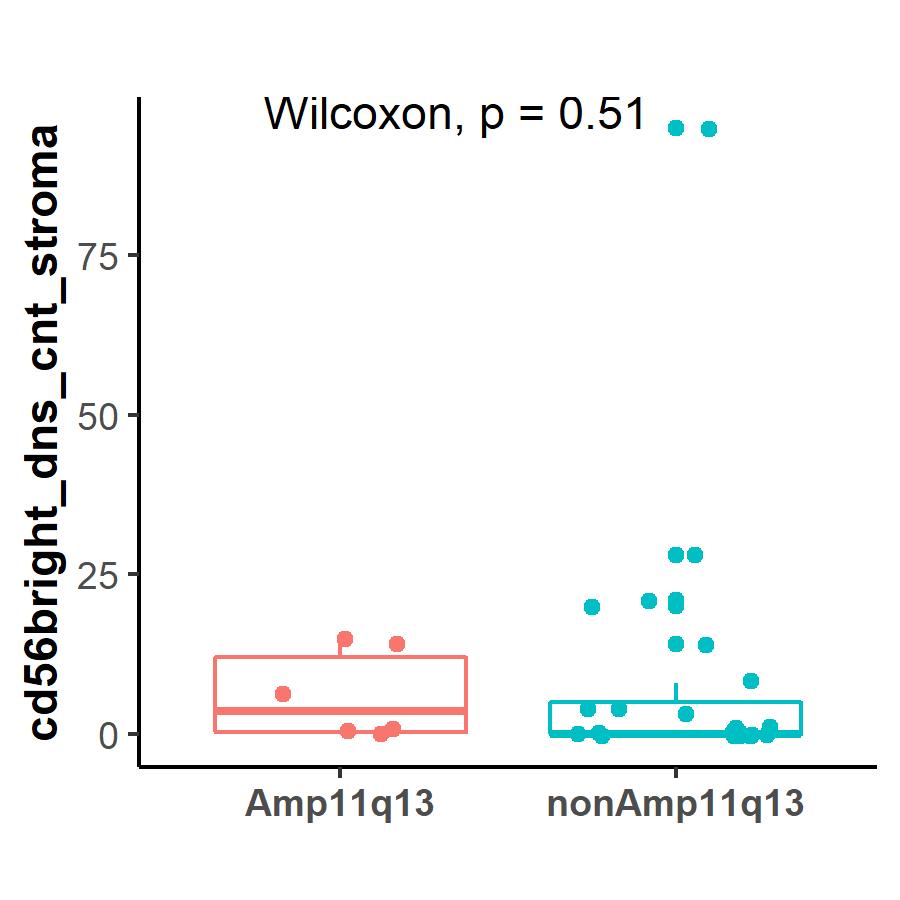

Supplement: Supplementary Figure 1 — Summary of frequently (Top 20) genomic characterized copy number alterations among 62 patients with HCC. [file DataSheet_1.zip › Immune_Infiltration_Estimation/cd56bright_dns_cnt_stroma_0.506_.jpeg]

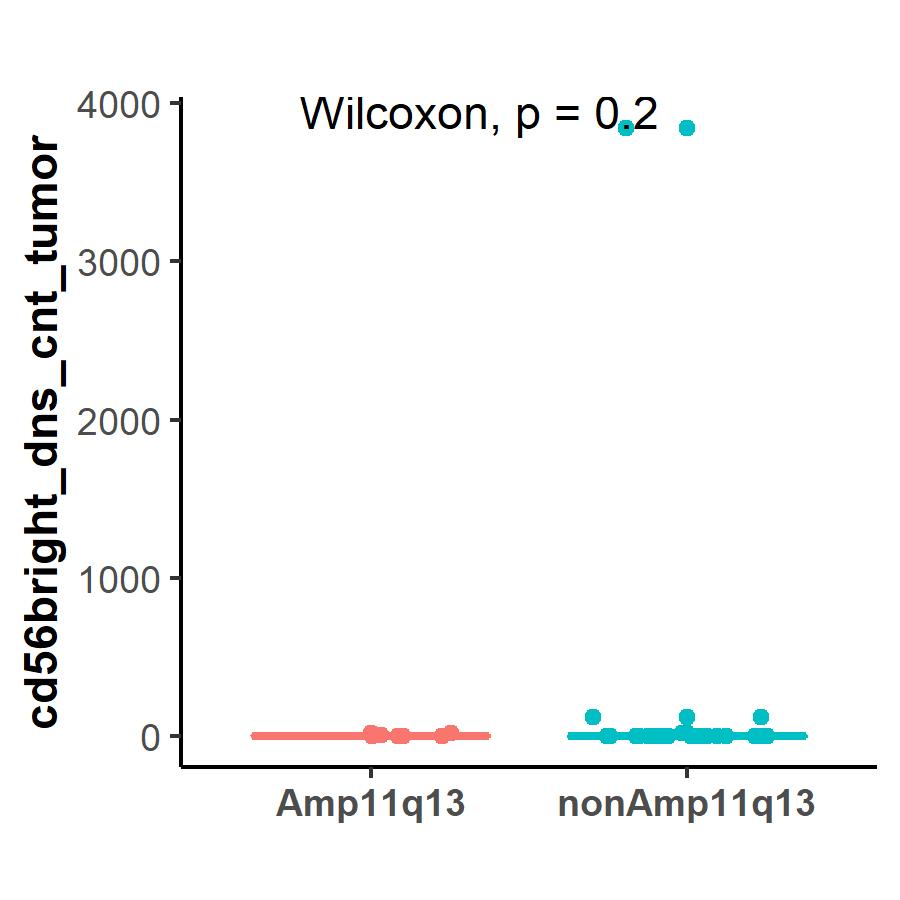

Supplement: Supplementary Figure 1 — Summary of frequently (Top 20) genomic characterized copy number alterations among 62 patients with HCC. [file DataSheet_1.zip › Immune_Infiltration_Estimation/cd56bright_dns_cnt_tumor_0.197_.jpeg]

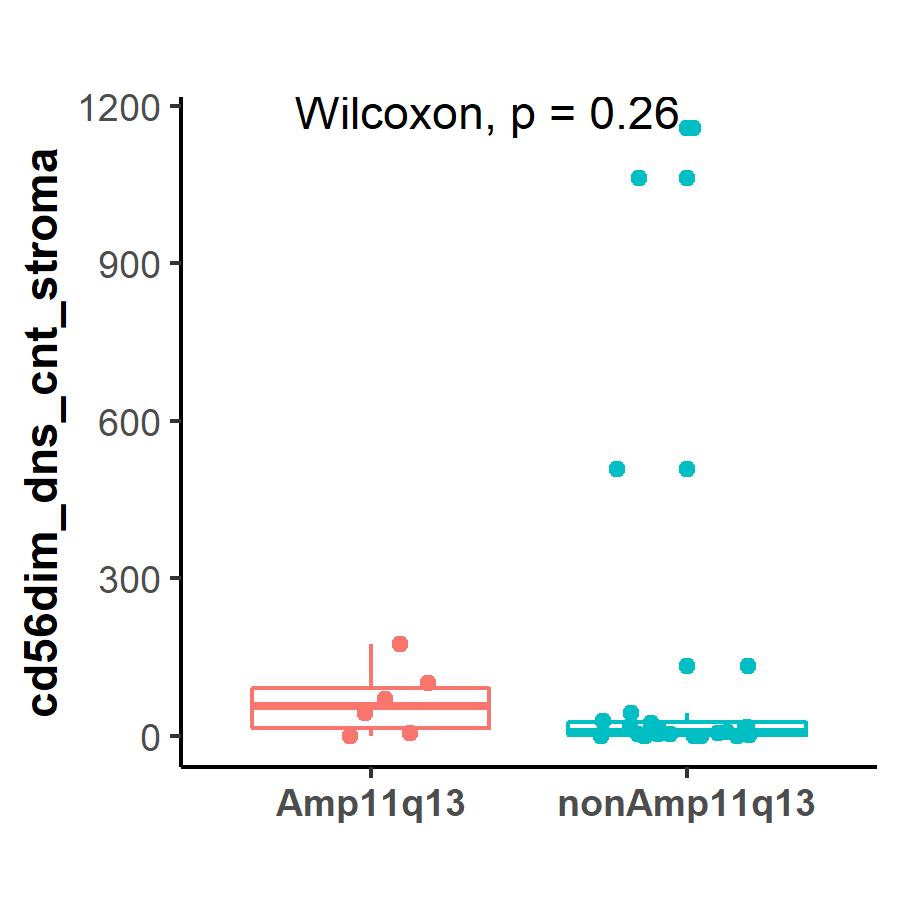

Supplement: Supplementary Figure 1 — Summary of frequently (Top 20) genomic characterized copy number alterations among 62 patients with HCC. [file DataSheet_1.zip › Immune_Infiltration_Estimation/cd56dim_dns_cnt_stroma_0.263_.jpeg]

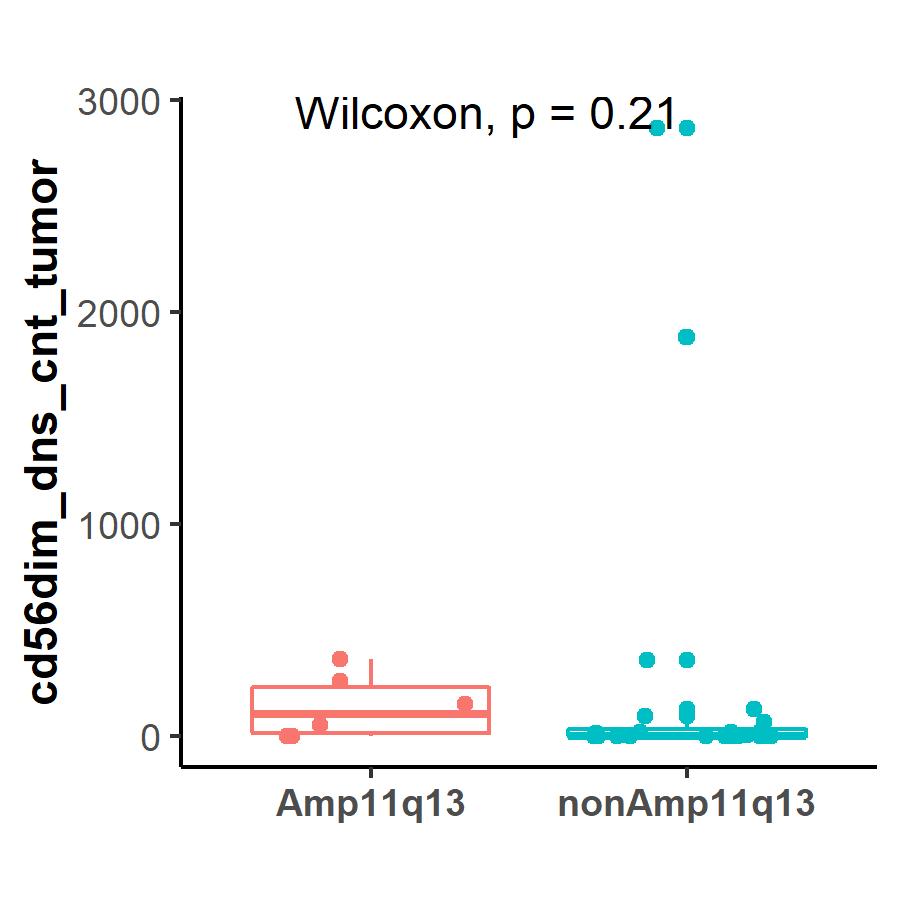

Supplement: Supplementary Figure 1 — Summary of frequently (Top 20) genomic characterized copy number alterations among 62 patients with HCC. [file DataSheet_1.zip › Immune_Infiltration_Estimation/cd56dim_dns_cnt_tumor_0.207_.jpeg]

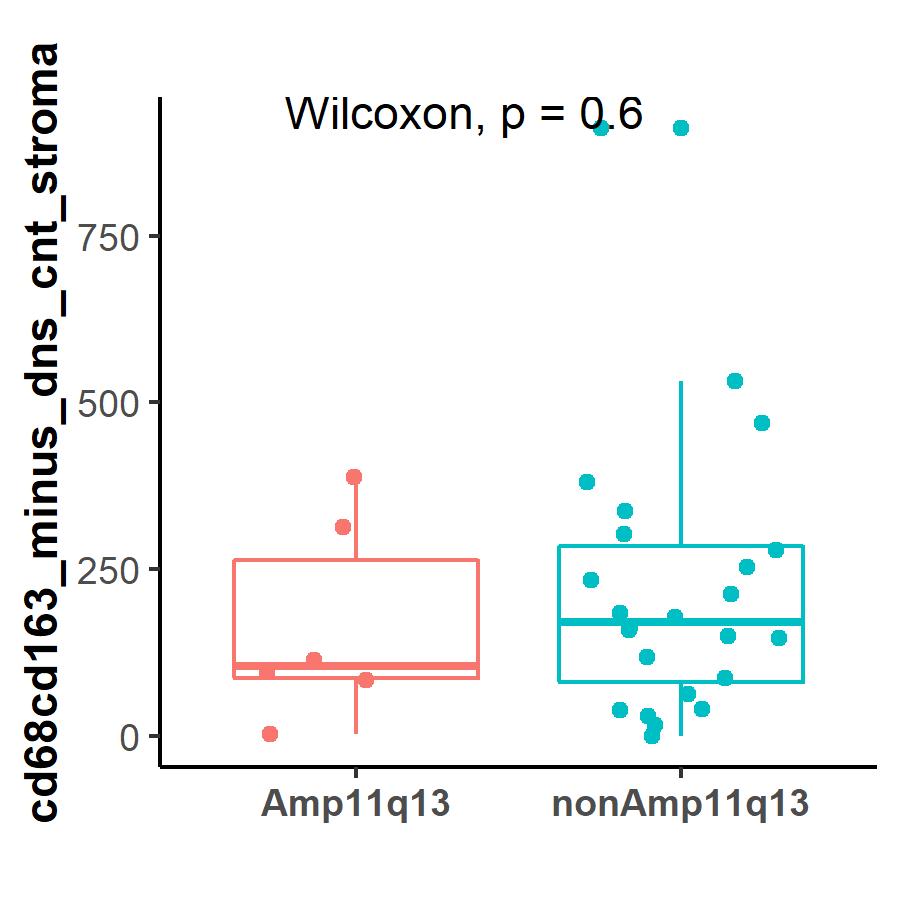

Supplement: Supplementary Figure 1 — Summary of frequently (Top 20) genomic characterized copy number alterations among 62 patients with HCC. [file DataSheet_1.zip › Immune_Infiltration_Estimation/cd68cd163_minus_dns_cnt_stroma_0.595_.jpeg]

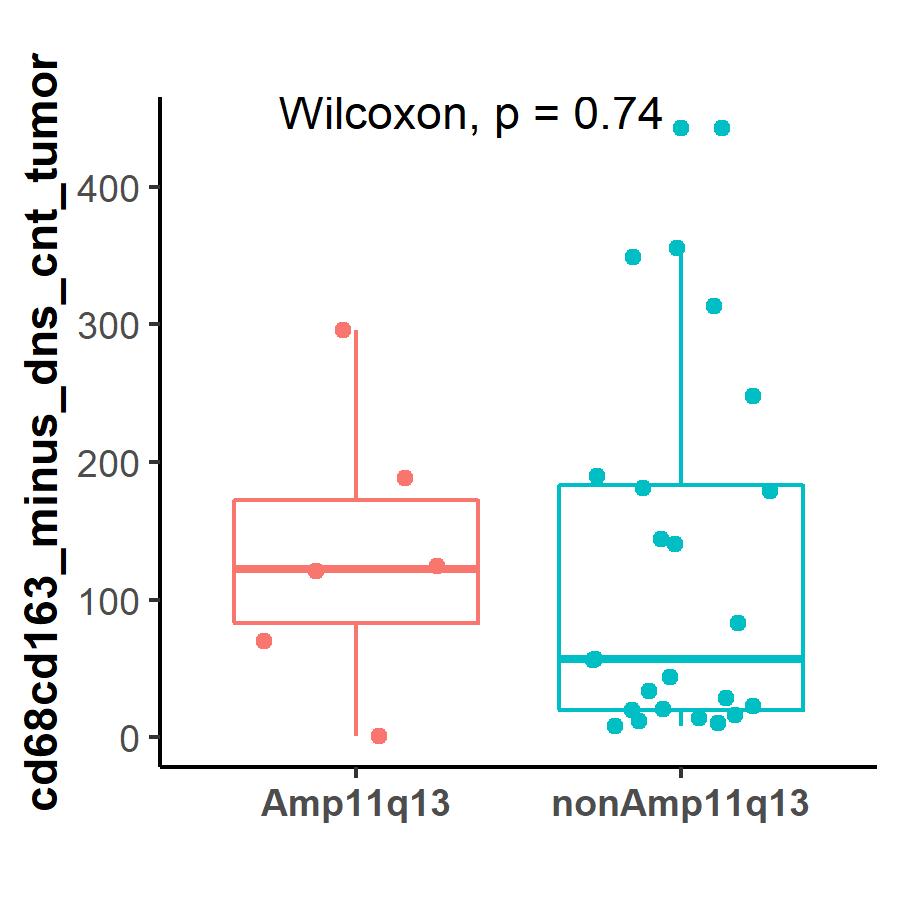

Supplement: Supplementary Figure 1 — Summary of frequently (Top 20) genomic characterized copy number alterations among 62 patients with HCC. [file DataSheet_1.zip › Immune_Infiltration_Estimation/cd68cd163_minus_dns_cnt_tumor_0.743_.jpeg]

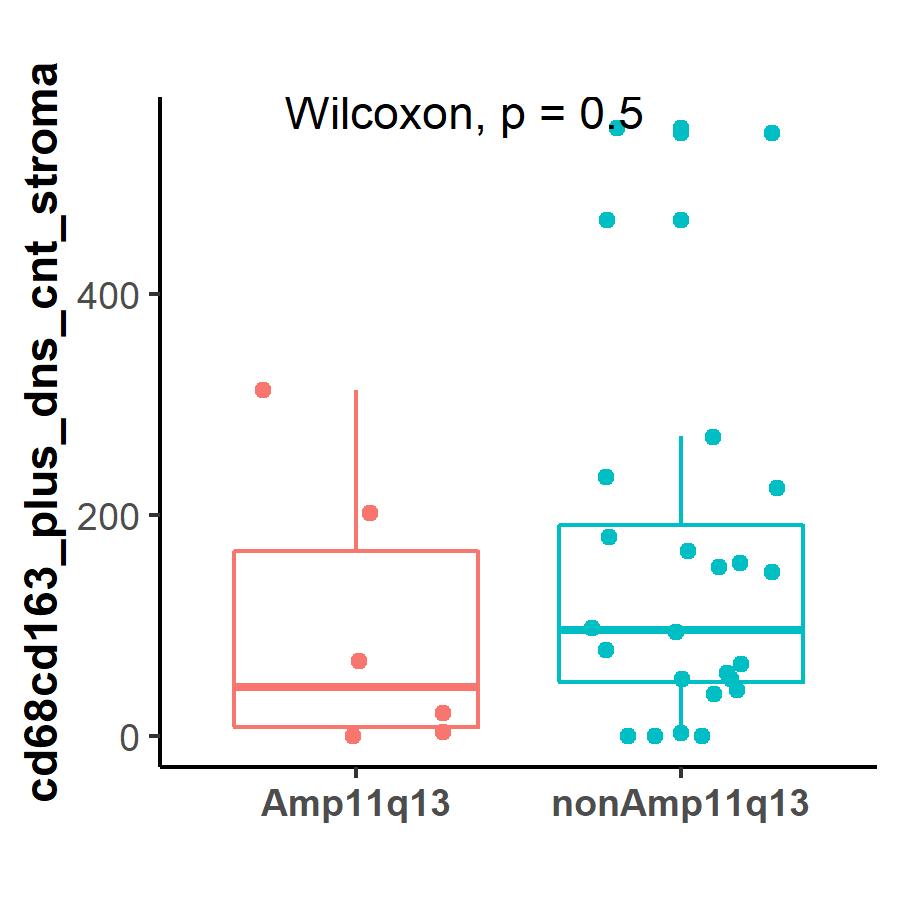

Supplement: Supplementary Figure 1 — Summary of frequently (Top 20) genomic characterized copy number alterations among 62 patients with HCC. [file DataSheet_1.zip › Immune_Infiltration_Estimation/cd68cd163_plus_dns_cnt_stroma_0.5_.jpeg]

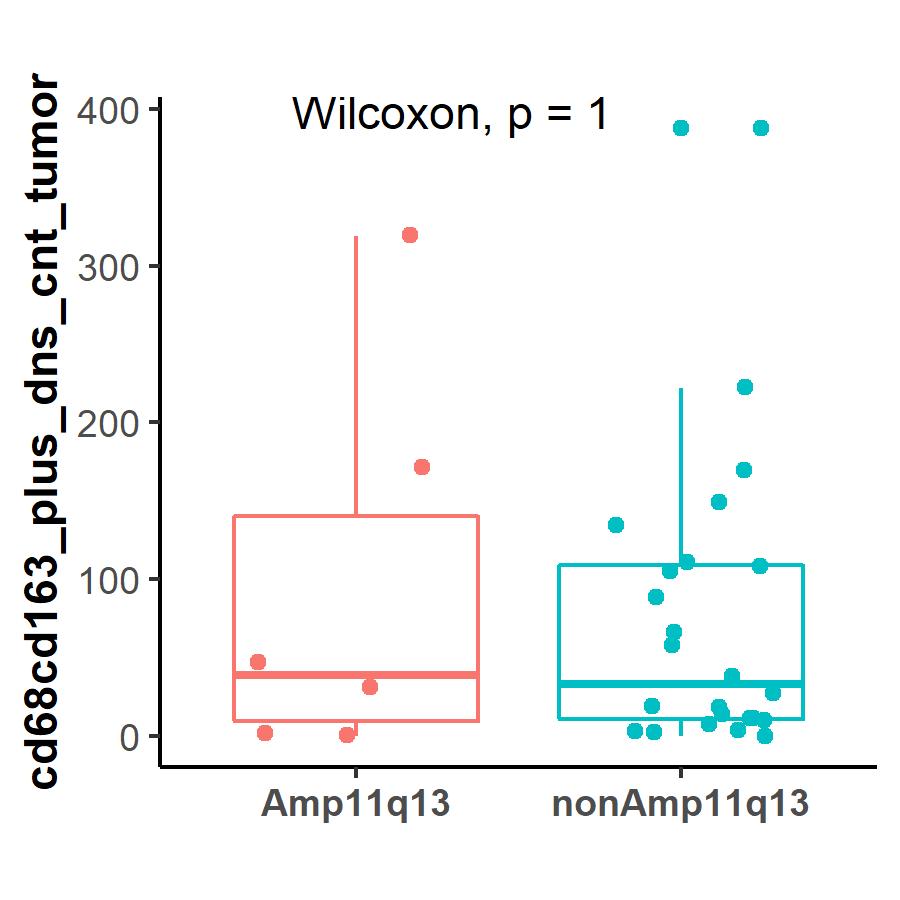

Supplement: Supplementary Figure 1 — Summary of frequently (Top 20) genomic characterized copy number alterations among 62 patients with HCC. [file DataSheet_1.zip › Immune_Infiltration_Estimation/cd68cd163_plus_dns_cnt_tumor_1_.jpeg]

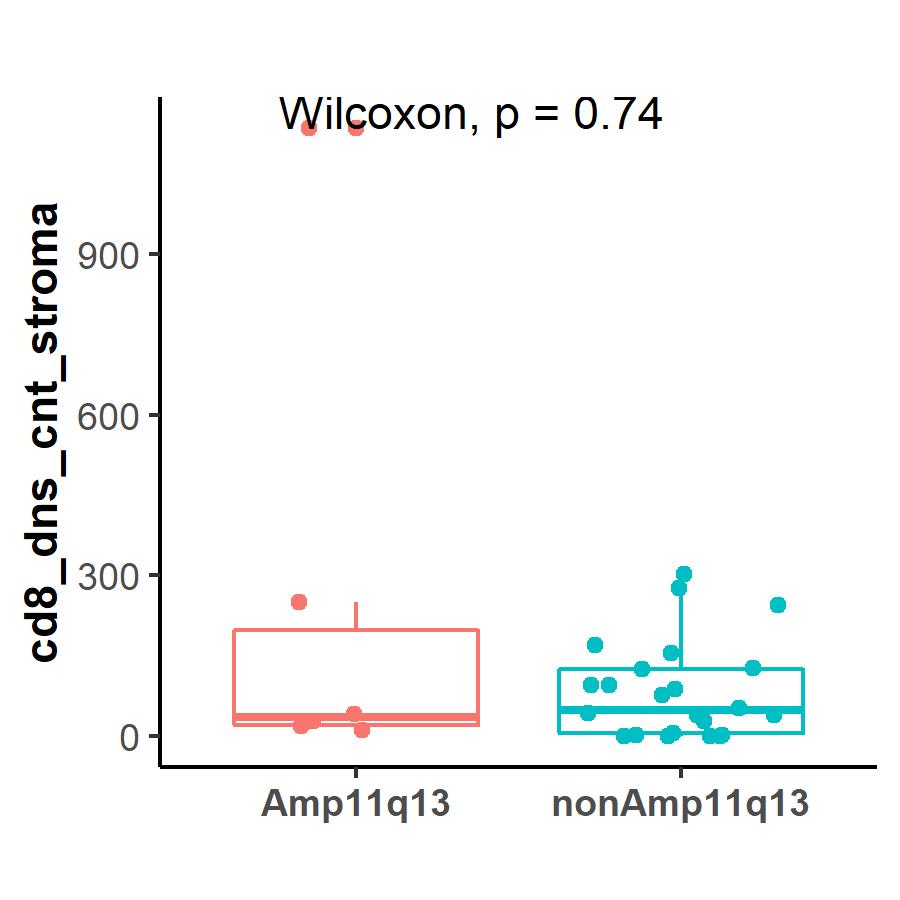

Supplement: Supplementary Figure 1 — Summary of frequently (Top 20) genomic characterized copy number alterations among 62 patients with HCC. [file DataSheet_1.zip › Immune_Infiltration_Estimation/cd8_dns_cnt_stroma_0.736_.jpeg]

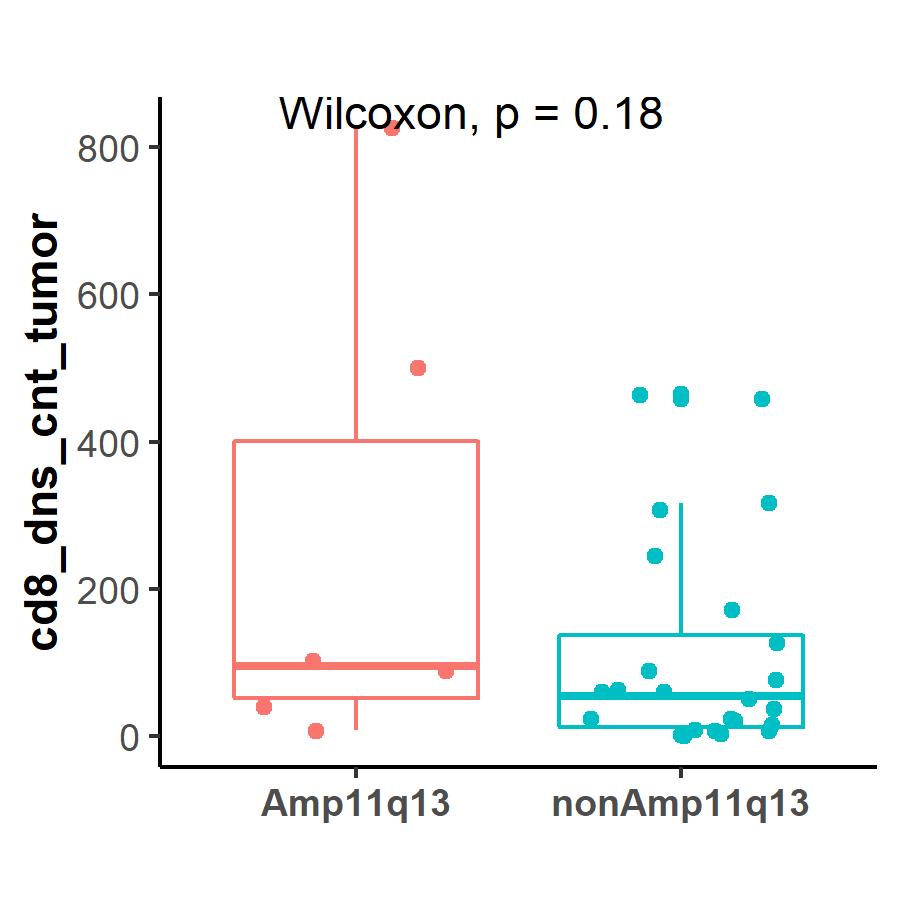

Supplement: Supplementary Figure 1 — Summary of frequently (Top 20) genomic characterized copy number alterations among 62 patients with HCC. [file DataSheet_1.zip › Immune_Infiltration_Estimation/cd8_dns_cnt_tumor_0.178_.jpeg]

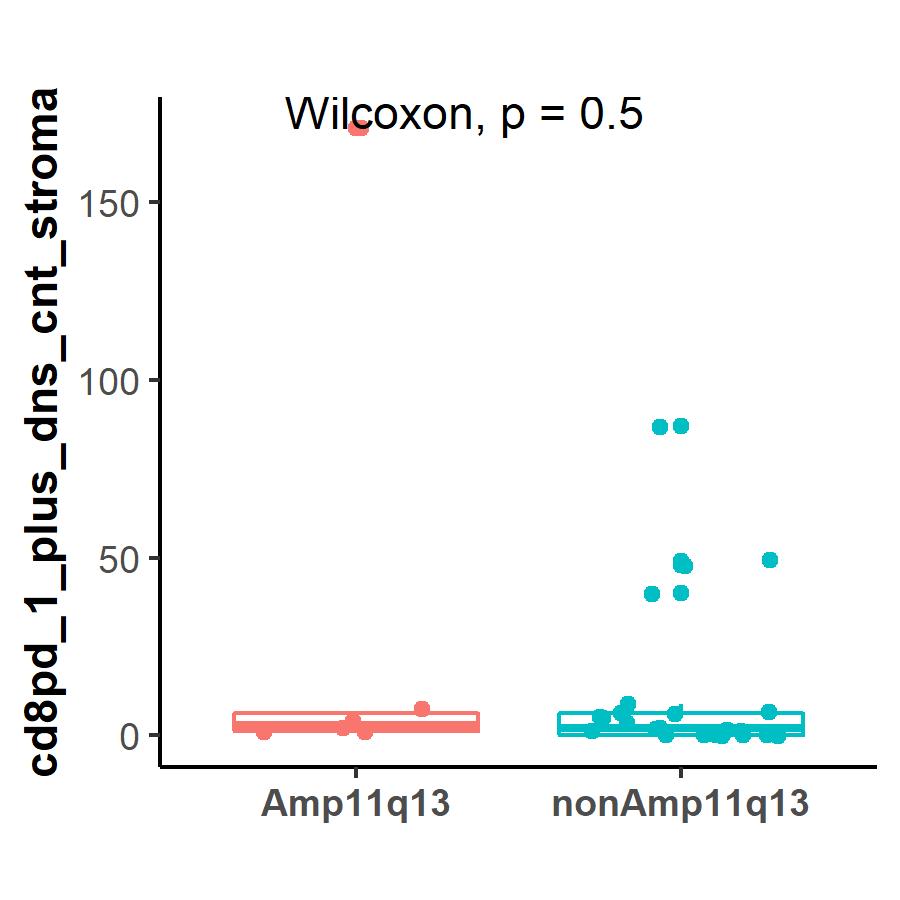

Supplement: Supplementary Figure 1 — Summary of frequently (Top 20) genomic characterized copy number alterations among 62 patients with HCC. [file DataSheet_1.zip › Immune_Infiltration_Estimation/cd8pd_1_plus_dns_cnt_stroma_0.495_.jpeg]

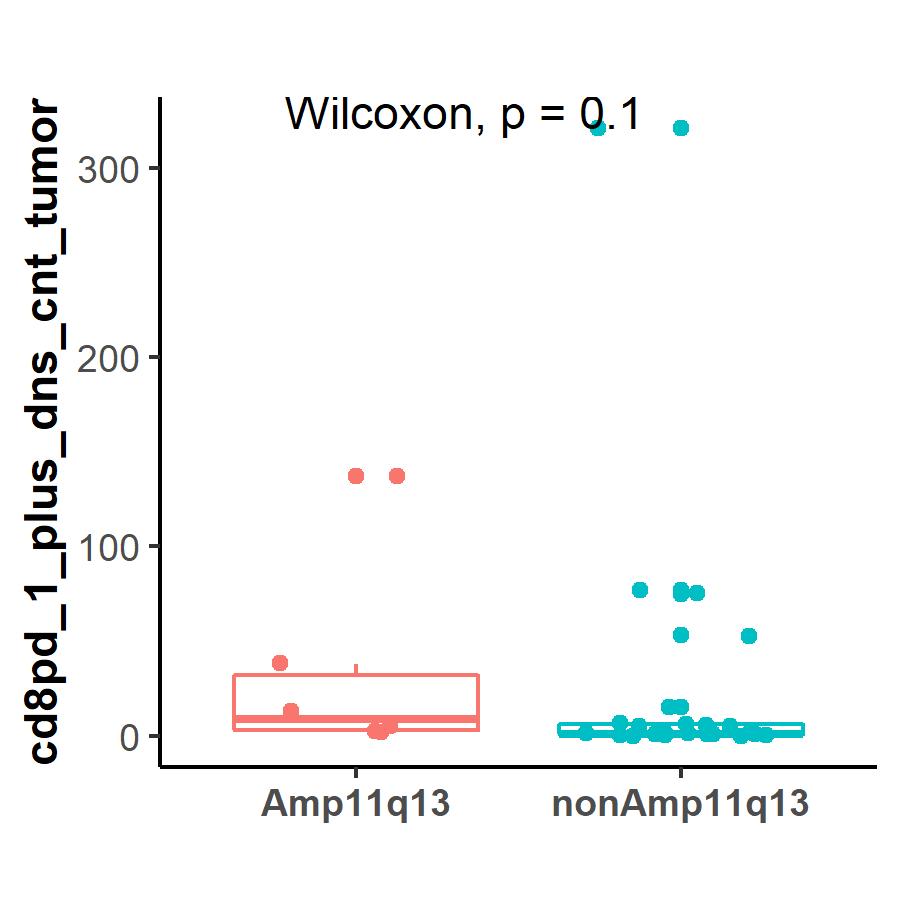

Supplement: Supplementary Figure 1 — Summary of frequently (Top 20) genomic characterized copy number alterations among 62 patients with HCC. [file DataSheet_1.zip › Immune_Infiltration_Estimation/cd8pd_1_plus_dns_cnt_tumor_0.103_.jpeg]

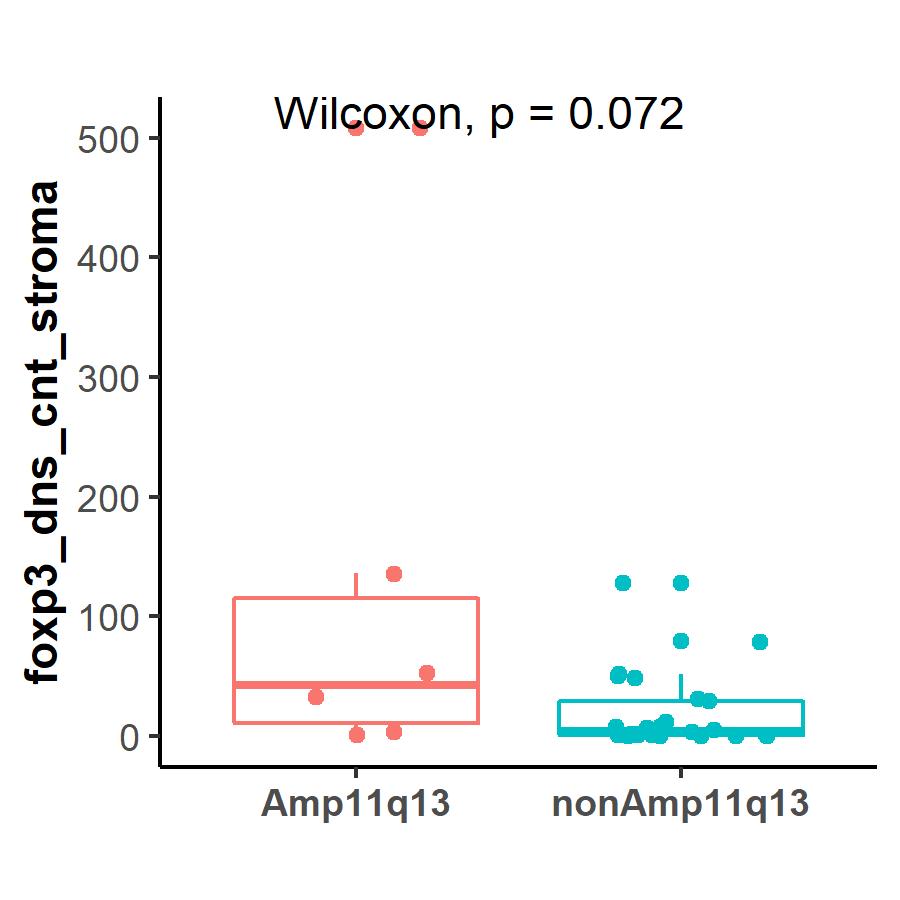

Supplement: Supplementary Figure 1 — Summary of frequently (Top 20) genomic characterized copy number alterations among 62 patients with HCC. [file DataSheet_1.zip › Immune_Infiltration_Estimation/foxp3_dns_cnt_stroma_0.072_.jpeg]

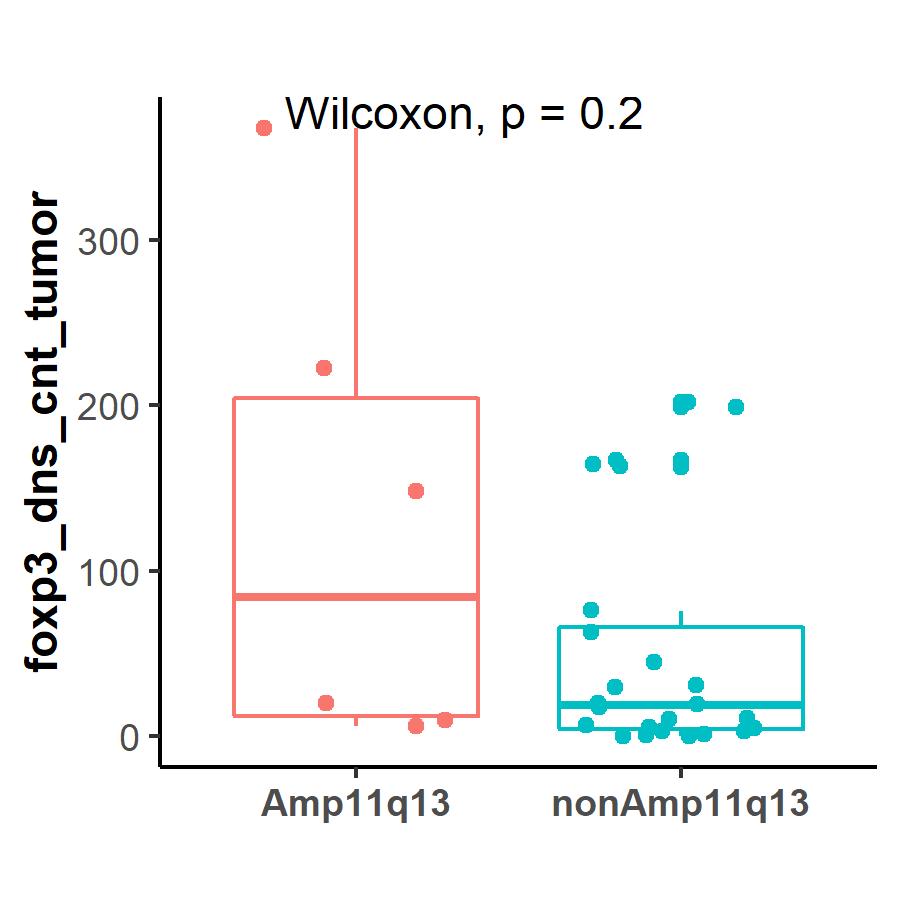

Supplement: Supplementary Figure 1 — Summary of frequently (Top 20) genomic characterized copy number alterations among 62 patients with HCC. [file DataSheet_1.zip › Immune_Infiltration_Estimation/foxp3_dns_cnt_tumor_0.204_.jpeg]

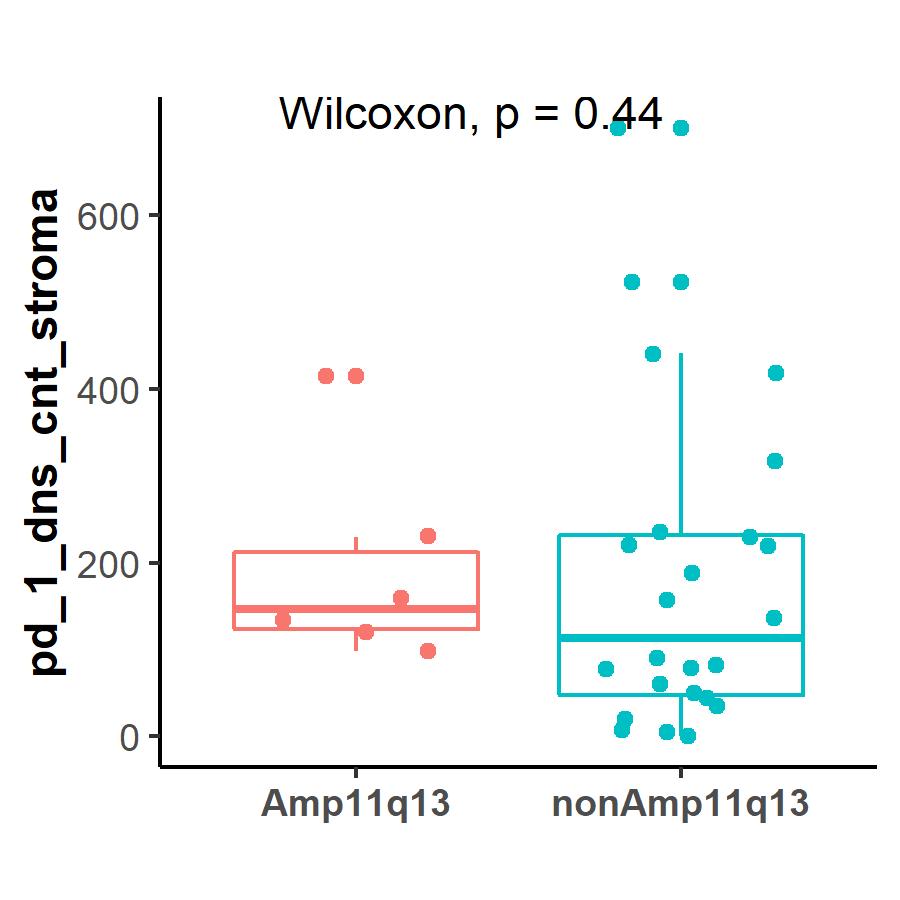

Supplement: Supplementary Figure 1 — Summary of frequently (Top 20) genomic characterized copy number alterations among 62 patients with HCC. [file DataSheet_1.zip › Immune_Infiltration_Estimation/pd_1_dns_cnt_stroma_0.437_.jpeg]

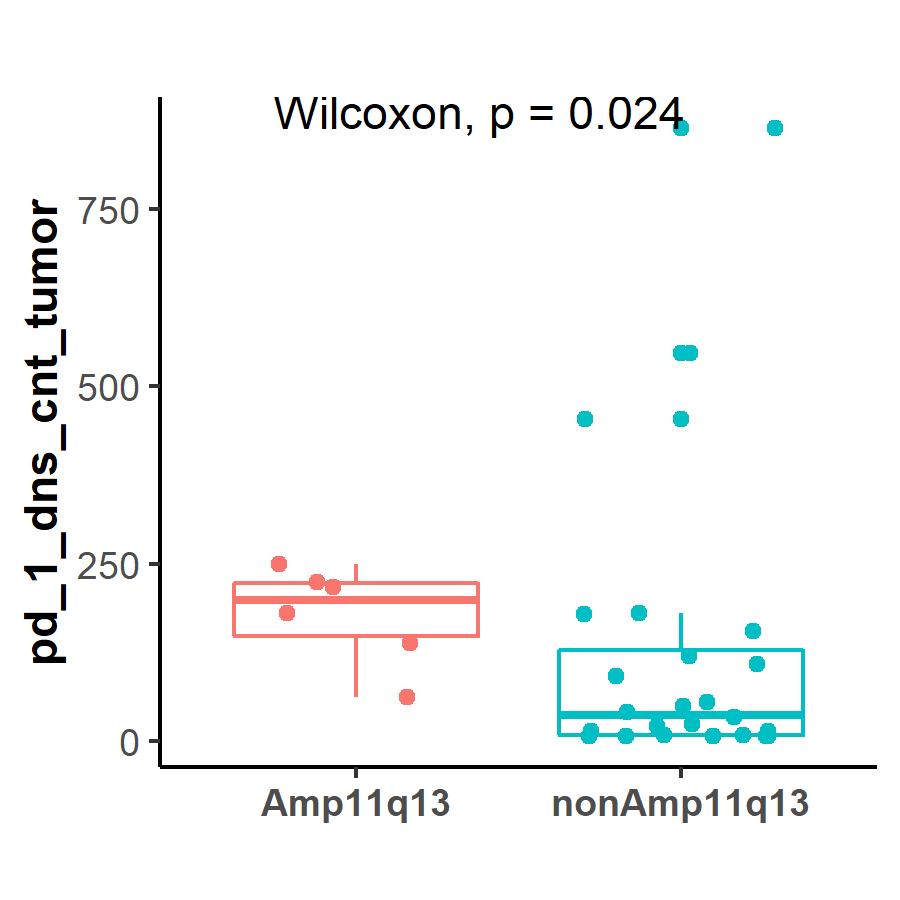

Supplement: Supplementary Figure 1 — Summary of frequently (Top 20) genomic characterized copy number alterations among 62 patients with HCC. [file DataSheet_1.zip › Immune_Infiltration_Estimation/pd_1_dns_cnt_tumor_0.024_.jpeg]

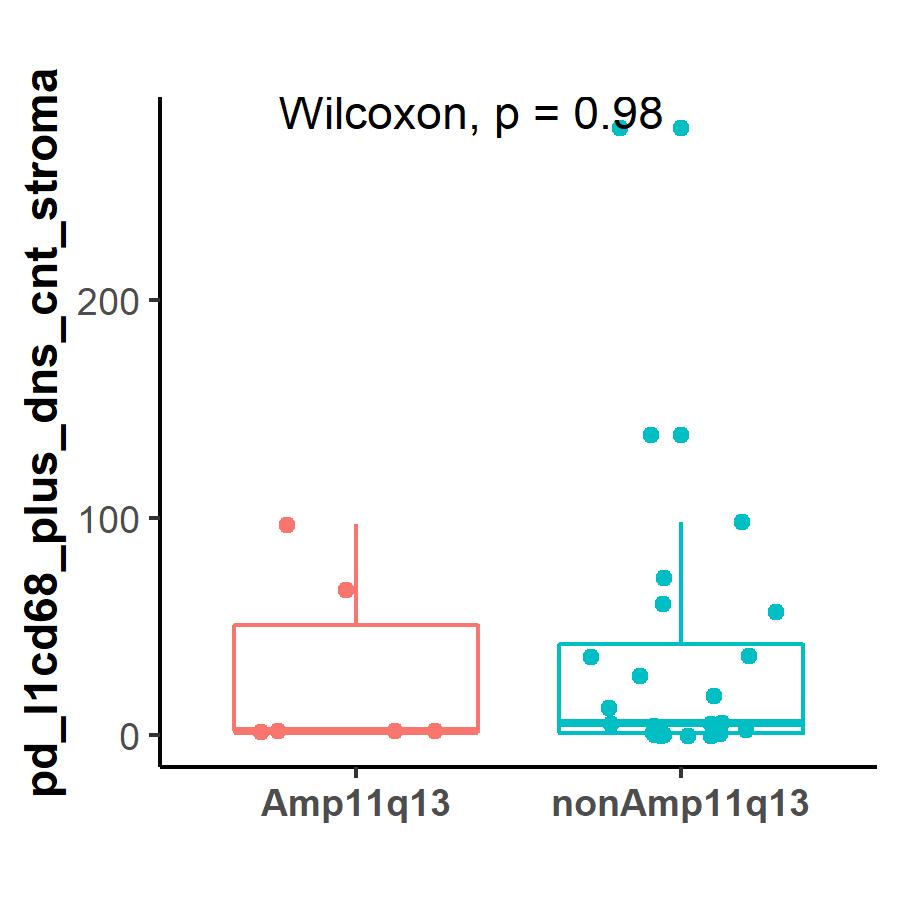

Supplement: Supplementary Figure 1 — Summary of frequently (Top 20) genomic characterized copy number alterations among 62 patients with HCC. [file DataSheet_1.zip › Immune_Infiltration_Estimation/pd_l1cd68_plus_dns_cnt_stroma_0.979_.jpeg]

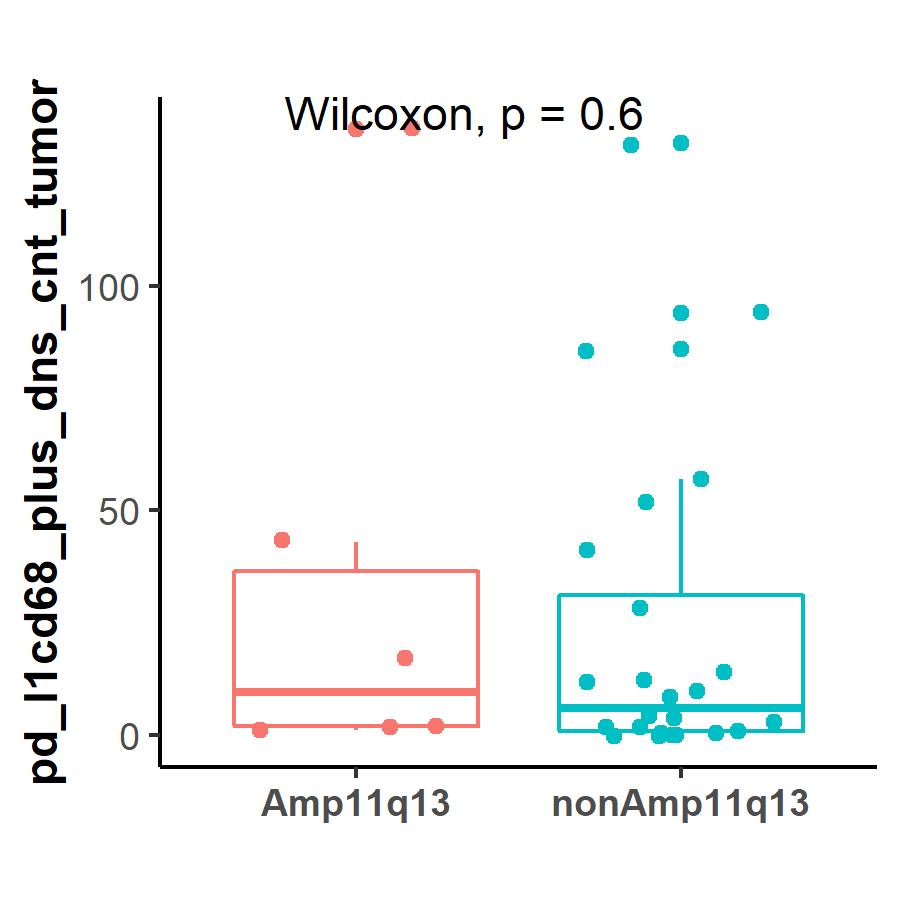

Supplement: Supplementary Figure 1 — Summary of frequently (Top 20) genomic characterized copy number alterations among 62 patients with HCC. [file DataSheet_1.zip › Immune_Infiltration_Estimation/pd_l1cd68_plus_dns_cnt_tumor_0.602_.jpeg]

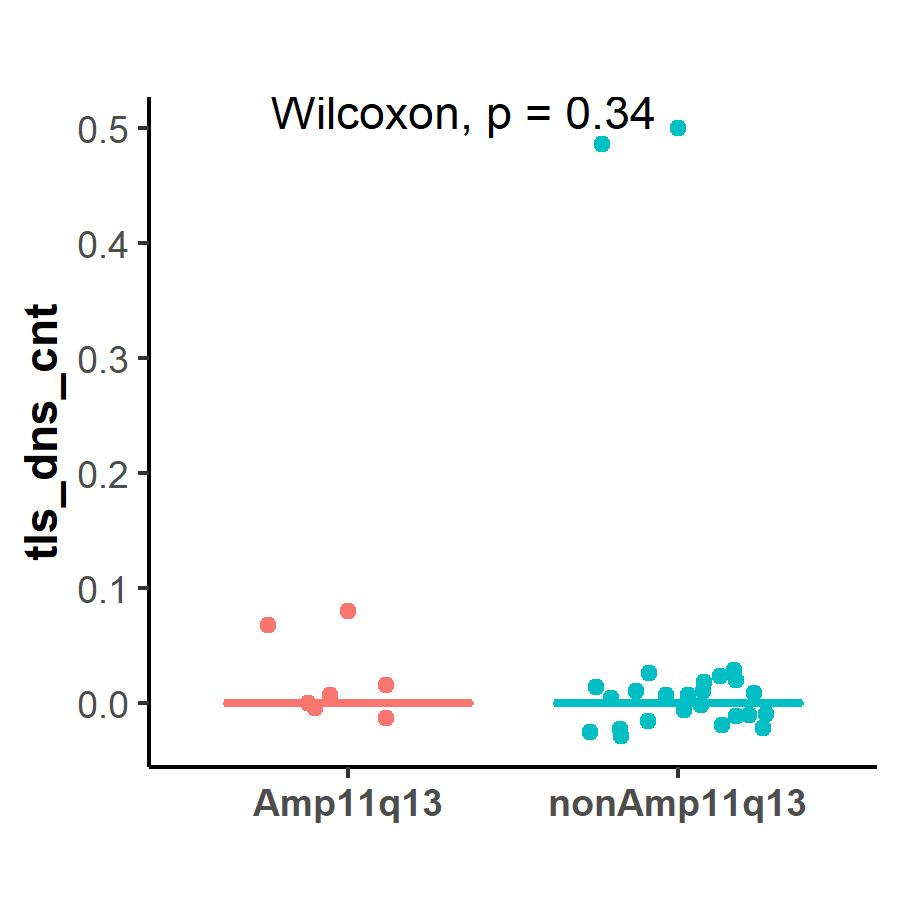

Supplement: Supplementary Figure 1 — Summary of frequently (Top 20) genomic characterized copy number alterations among 62 patients with HCC. [file DataSheet_1.zip › Immune_Infiltration_Estimation/tls_dns_cnt_0.338_.jpeg]

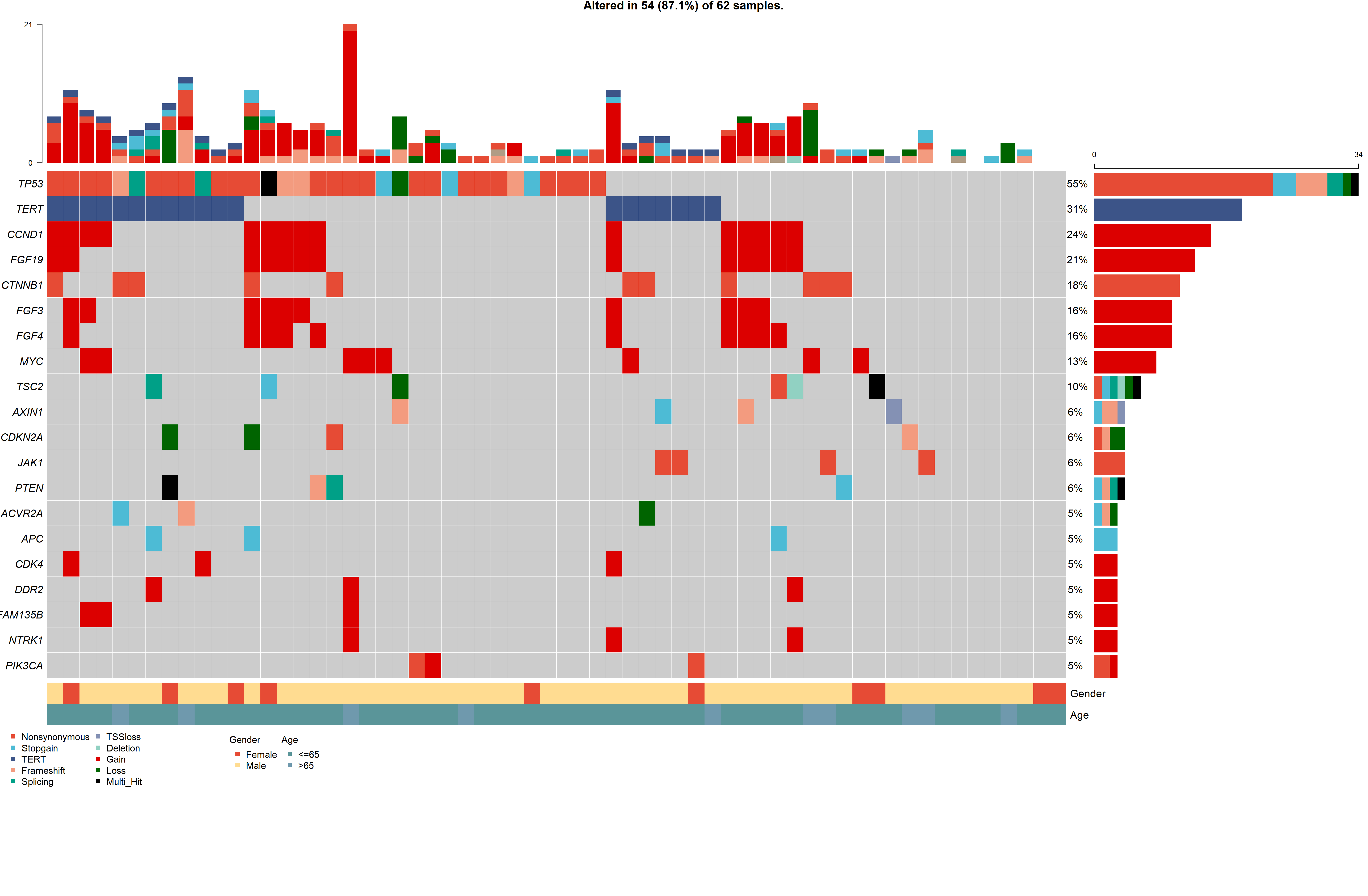

Supplement: Supplementary Figure 1 — Summary of frequently (Top 20) genomic characterized copy number alterations among 62 patients with HCC. [file DataSheet_1.zip › mutation_profile/Fig2_all_rmGermline_oncoprint_20220716.tiff]

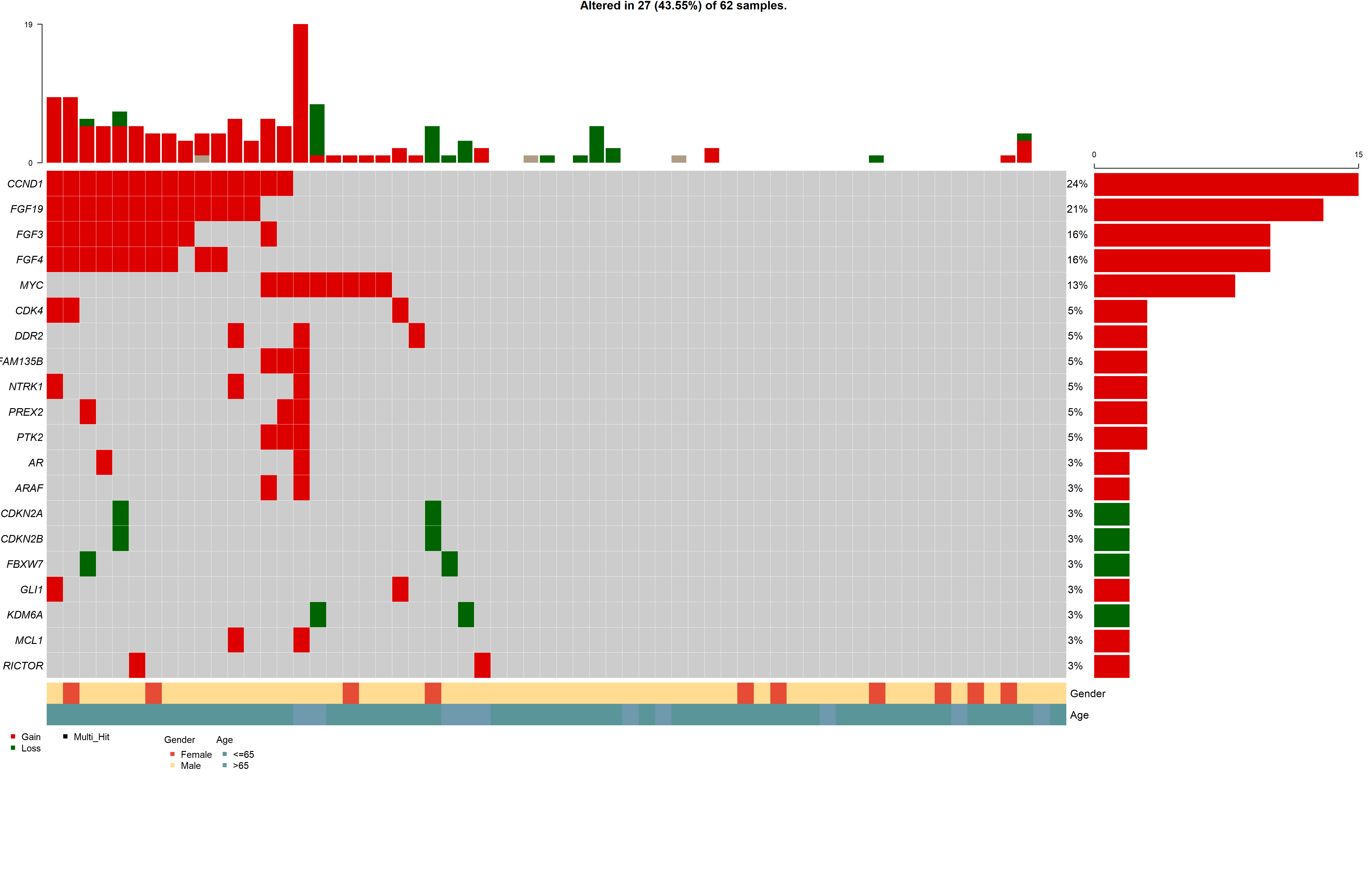

Supplement: Supplementary Figure 1 — Summary of frequently (Top 20) genomic characterized copy number alterations among 62 patients with HCC. [file DataSheet_1.zip › mutation_profile/Fig2_cnv_fusion_rmGermline_oncoprint_20220716.tiff]

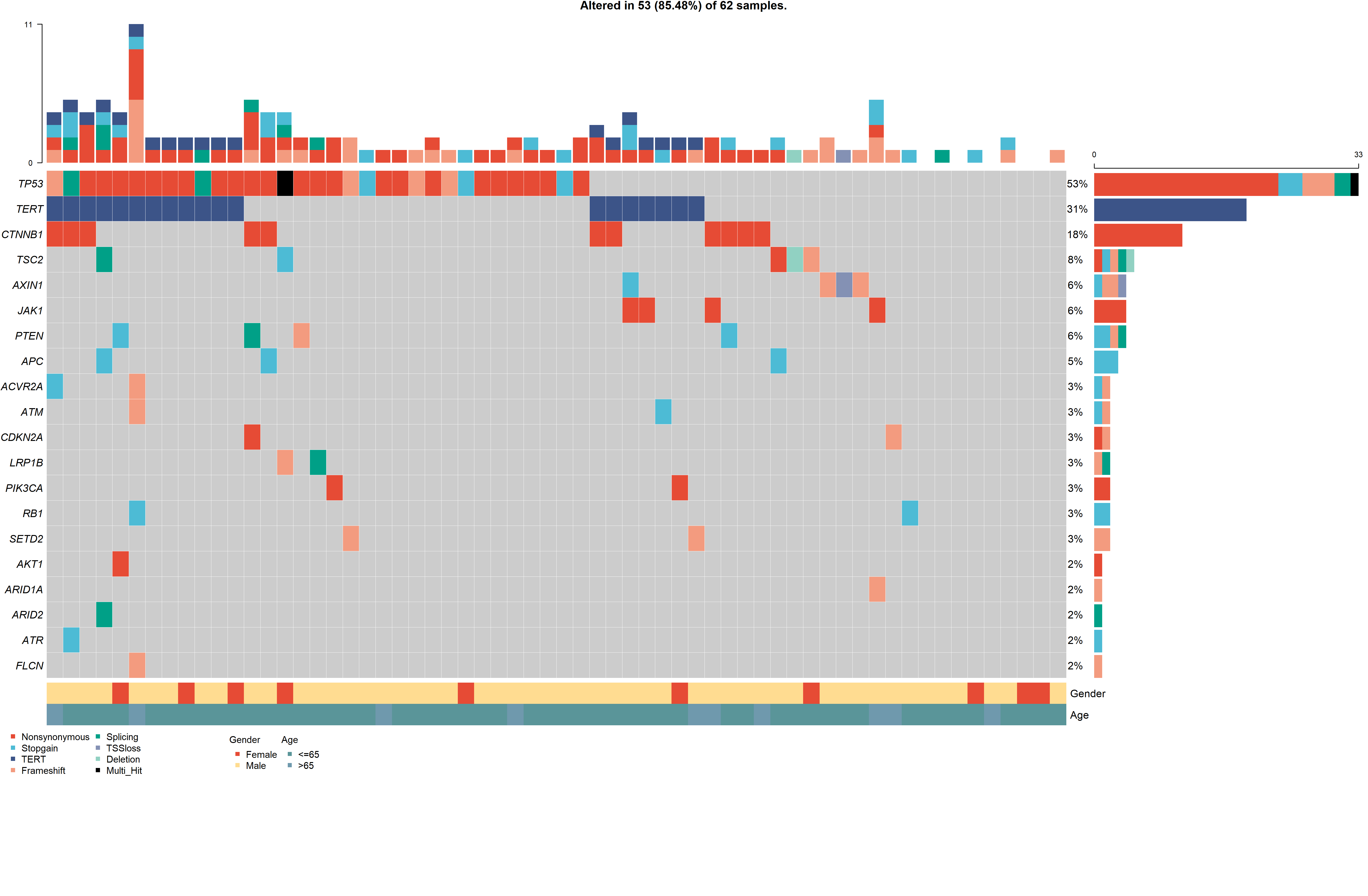

Supplement: Supplementary Figure 1 — Summary of frequently (Top 20) genomic characterized copy number alterations among 62 patients with HCC. [file DataSheet_1.zip › mutation_profile/Fig2_snv_rmGermline_oncoprint_20220716.tiff]

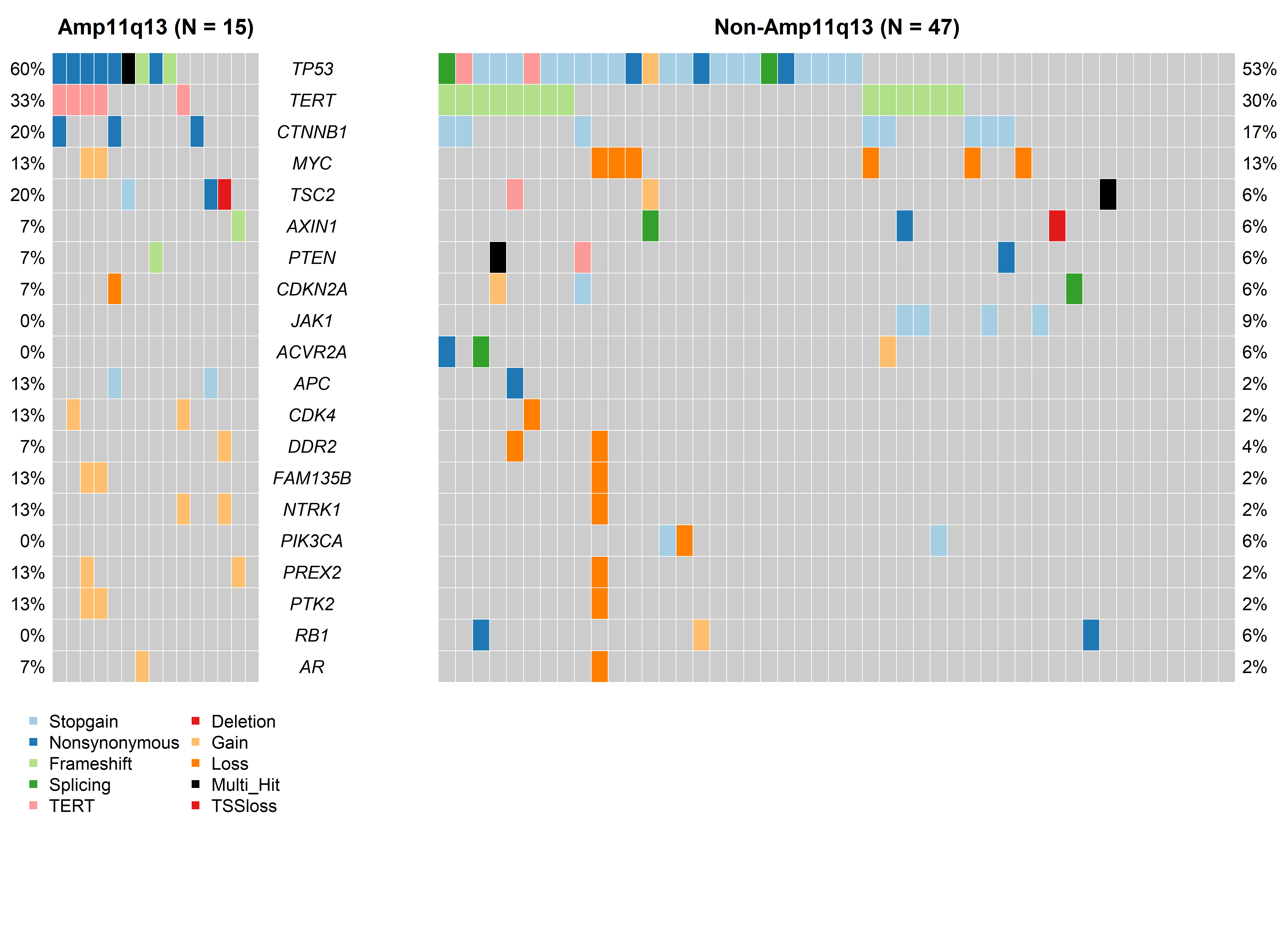

Supplement: Supplementary Figure 1 — Summary of frequently (Top 20) genomic characterized copy number alterations among 62 patients with HCC. [file DataSheet_1.zip › mutation_profile/Supplementary Figure 1 _Amp11q13_coOncoplot_20220821.tiff]
