# Supplementary material for: Chromosome 11q13 amplification correlates with poor response and prognosis to PD-1 blockade in unresectable hepatocellular carcinoma
Source: Front Immunol. 2023 Mar 28;14:1116057. doi: 10.3389/fimmu.2023.1116057 (PMC10086239; doi:10.3389/fimmu.2023.1116057)
Supplement: Supplementary file 2 [file DataSheet_2.docx]

Fig S1


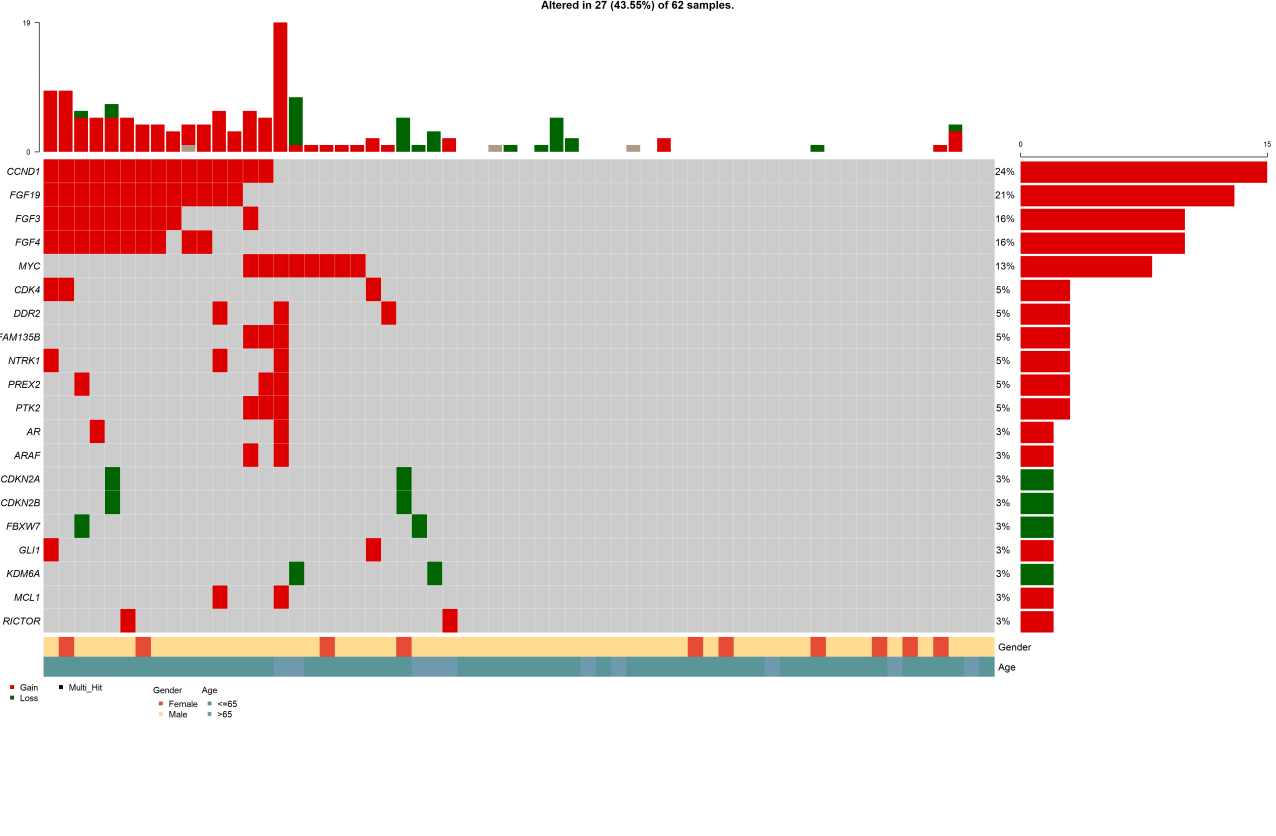


Fig S2


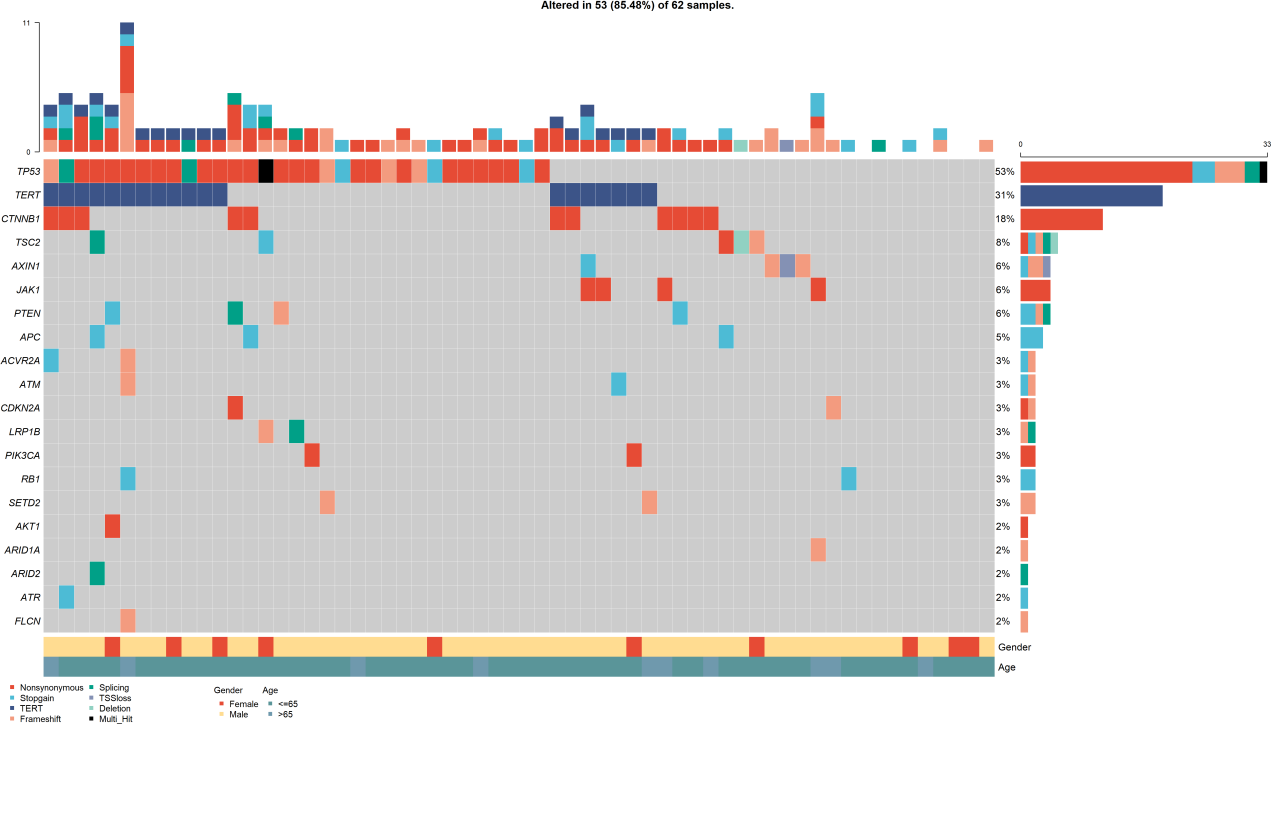


Fig S3


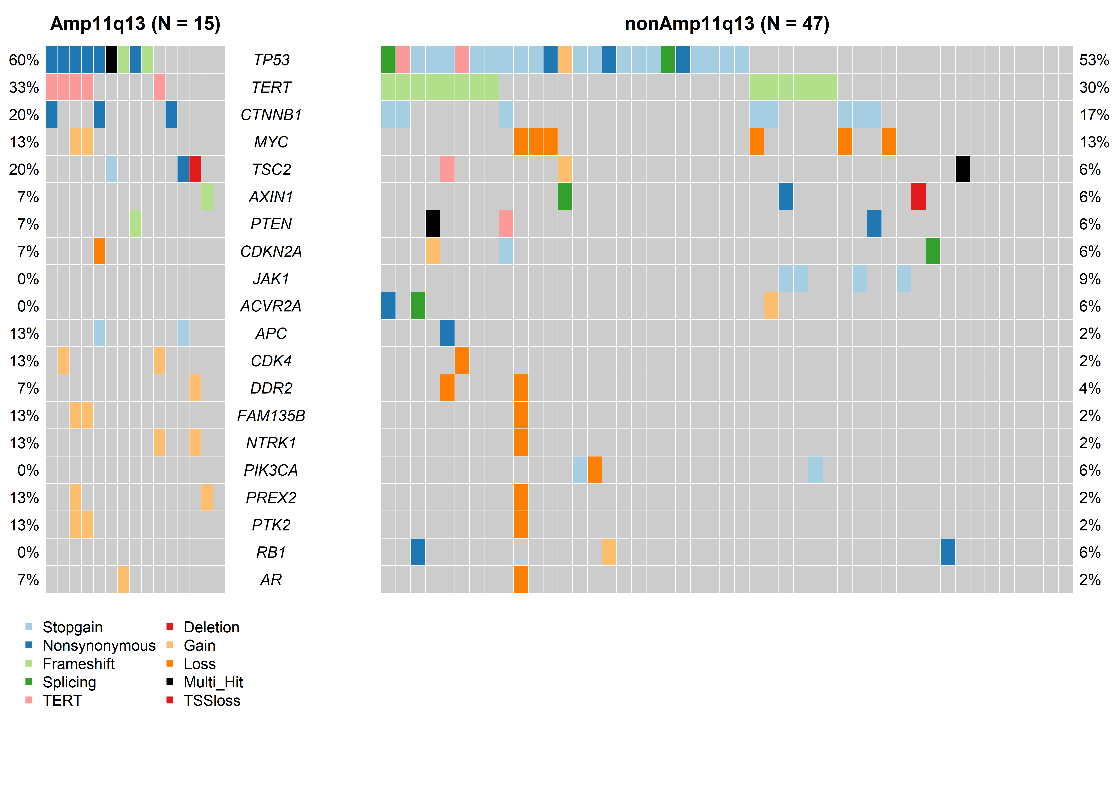


Fig S4


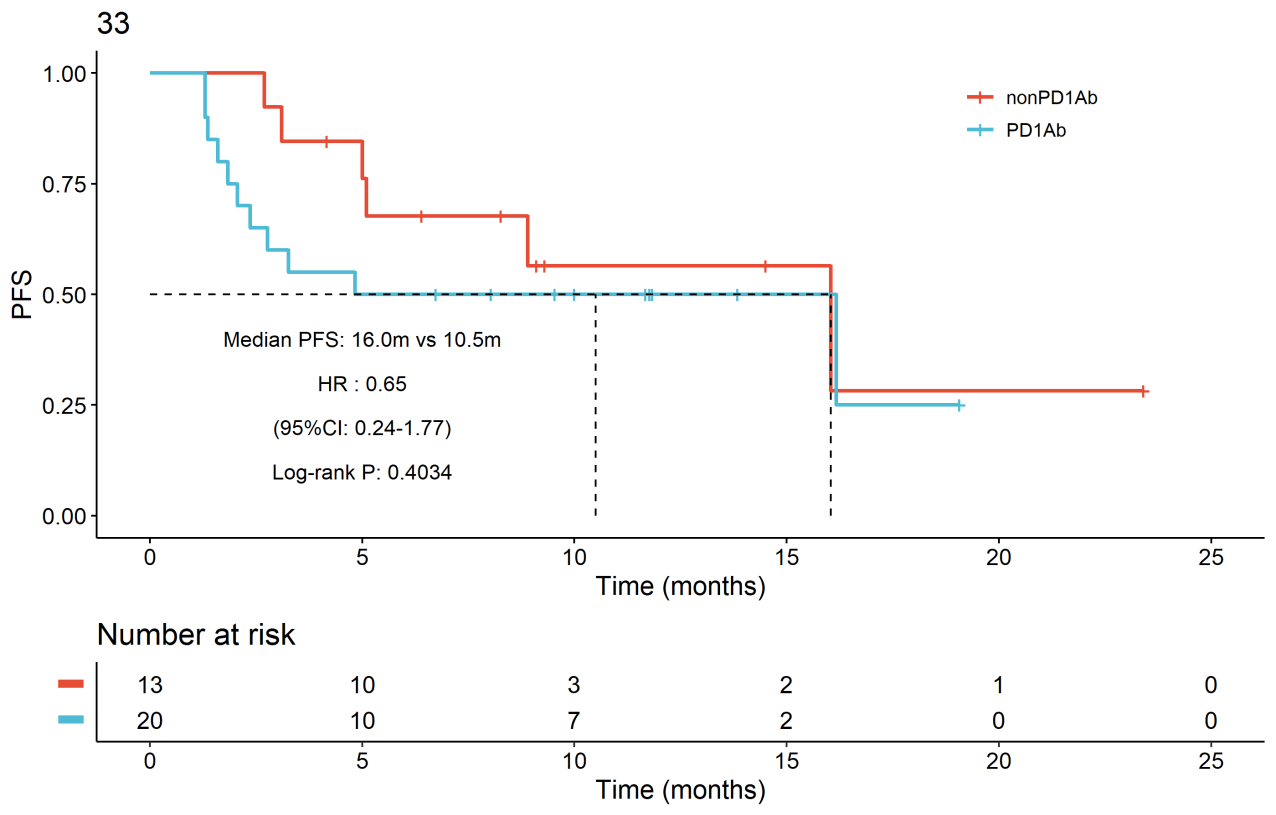


Fig S5


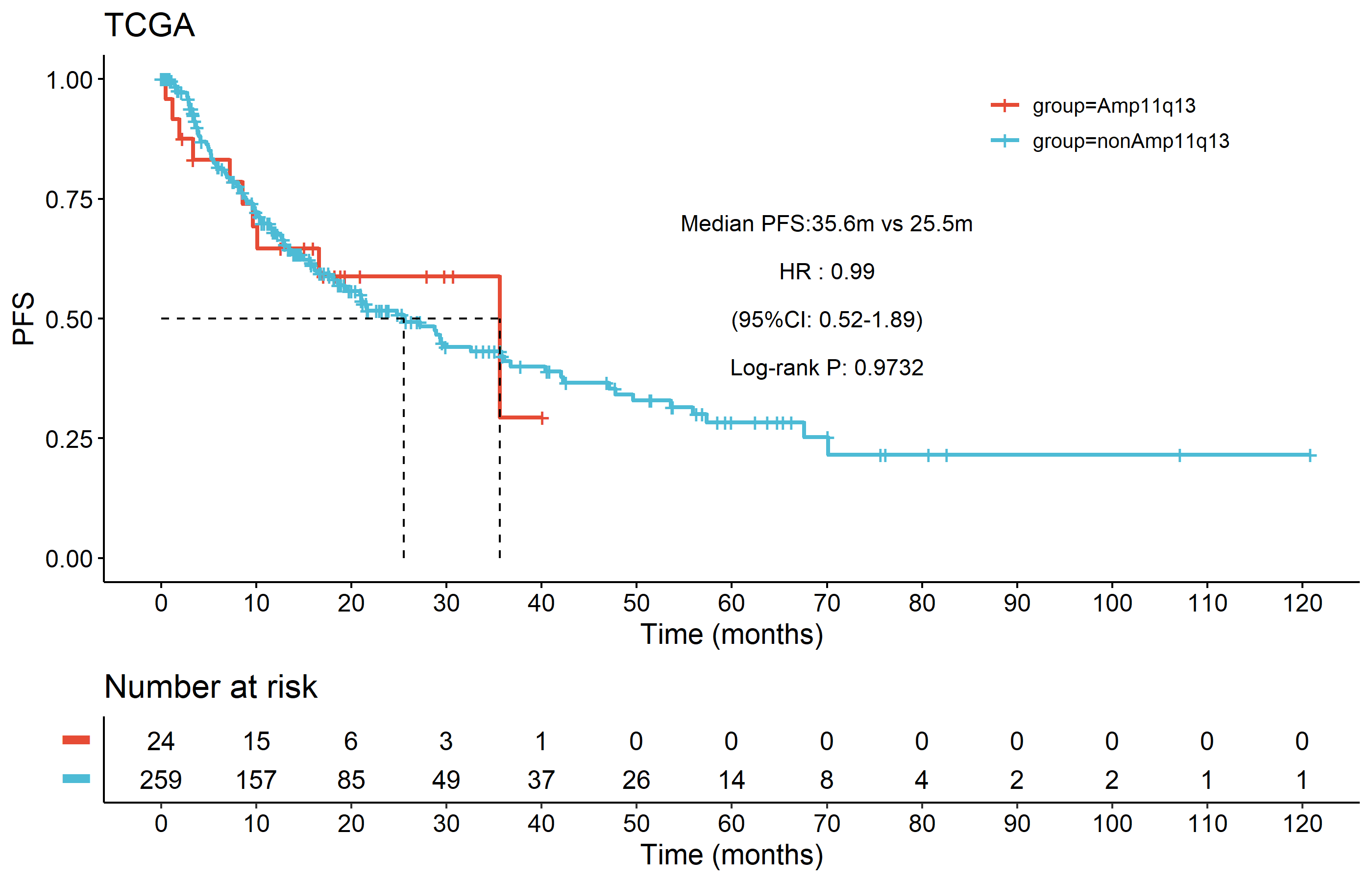


Fig S6


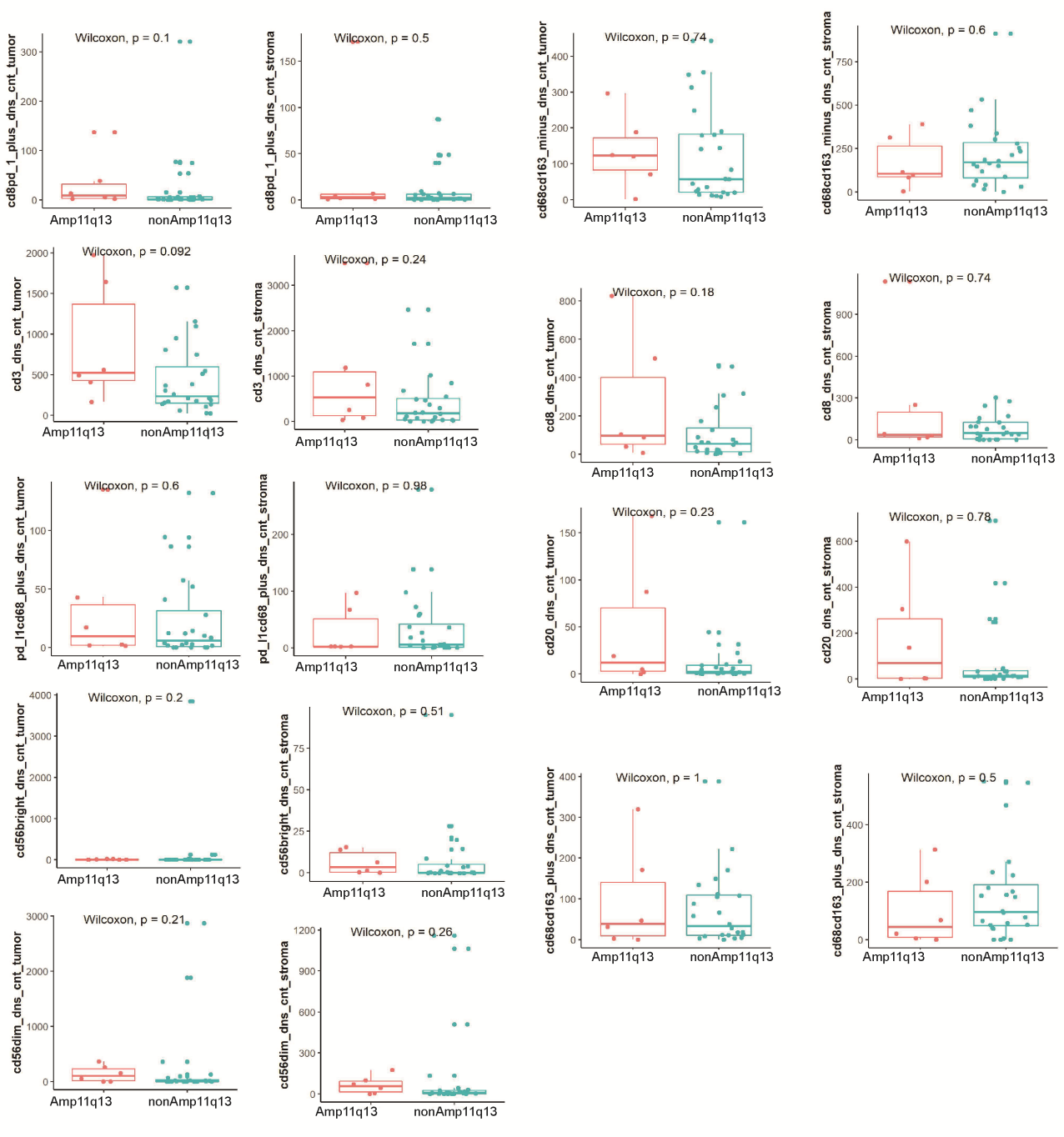


Fig S7


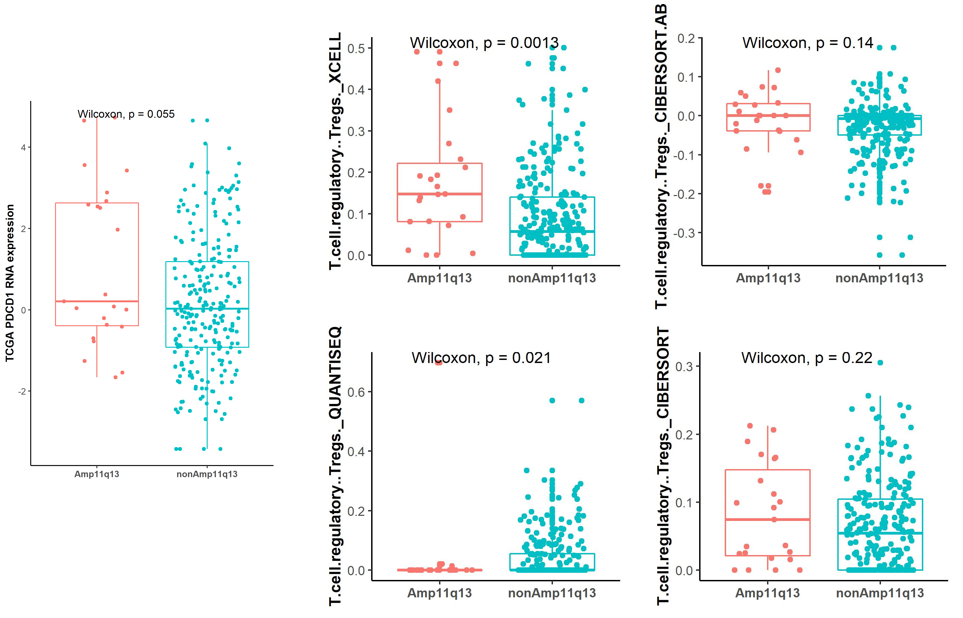


Table S1

|  | Case No. | Therapy strategies | | |
| --- | --- | --- | --- | --- |
|  |  | Locoregional therapy | Targeted therapies | ICIs therapy |
| First-line ICIs therapy | 1 | TACE | Lenvatinib | Toripalimab |
|  | 2 | No | Lenvatinib | Toripalimab |
|  | 3 | No | Regorafenib | Toripalimab |
|  | 4 | No | Lenvatinib | Camrelizumab |
|  | 5 | TACE | No | Camrelizumab |
|  | 6 | TAE | No | Camrelizumab |
|  | 7 | TACE；RT | Lenvatinib | Camrelizumab |
|  | 8 | TACE | Lenvatinib | Camrelizumab |
|  | 9 | TACE；RT | Lenvatinib | Camrelizumab |
|  | 10 | TACE；PVE | Lenvatinib | Toripalimab |
|  | 11 | TACE | Lenvatinib | Toripalimab |
|  | 12 | TACE | No | Sintilimab |
|  | 13 | TACE | Lenvatinib | Toripalimab |
|  | 14 | TACE；RT | Lenvatinib | Pembrolizumab |
|  | 15 | TACE | Lenvatinib | Camrelizumab |
| Second-line ICIs therapy | 16 | TACE | No | Toripalimab |
|  | 17 | RT | Regorafenib | Sintilimab |
|  | 18 | No | Lenvatinib | Toripalimab |
|  | 19 | No | Bevacizumab | Sintilimab |
|  | 20 | Ablation | Regorafenib | Camrelizumab |

Annotations: ICIs, immune checkpoint inhibitors; TACE, transcatheter arterial chemoembolization; RT, radiotherapy

Table S2

| Characteristics | Number of Cases (%) | Number of Amp11q13 Cases (%) | Number of nonAmp11q13 Cases (%) | *P* value |
| --- | --- | --- | --- | --- |
| Age |  |  |  | 0.93 |
| <=60 | 26(78.8) | 7(77.8) | 19(79.2) |  |
| >60 | 7(21.2) | 2(22.2) | 5(20.8) |  |
| Gender |  |  |  | 0.52 |
| male | 27(81.8) | 8(88.9) | 19(79.2) |  |
| female | 6(18.2) | 1(11.1) | 5(20.8) |  |
| Background liver disease | |  |  | 0.82 |
| Non | 23(69.7) | 6(66.7) | 17(70.8) |  |
| Yes | 10(30.3) | 3(33.3) | 7(29.2) |  |
| AFP |  |  |  | 0.94 |
| <200 | 15(45.5) | 4(44.4) | 11(45.8) |  |
| >=200 | 18(54.5) | 5(55.6) | 13(54.2) |  |
| DCP |  |  |  | 0.14 |
| <200 | 10(30.3) | 1(11.1) | 9(37.5) |  |
| >=200 | 20(60.6) | 7(77.8) | 13(54.2) |  |
| NA | 3(9.1) | 1(11.1) | 2(8.3) |  |
| BCLC stage |  |  |  | 0.55 |
| A and B | 21(63.6) | 5(55.6) | 16(66.7) |  |
| C | 12(36.4) | 4(44.4) | 8(33.3) |  |
| Tumor number | |  |  | 0.28 |
| 1 | 8(24.2) | 1(11.1) | 7(29.2) |  |
| multiple | 25(75.8) | 8(88.9) | 17(70.8) |  |
| Size |  |  |  | 0.13 |
| <=5 | 18(54.5) | 3(33.3) | 15(62.5) |  |
| >5 | 15(45.5) | 6(66.7) | 9(37.5) |  |
| Portal vein tumor thrombosis | | |  | 0.17 |
| Non | 24(72.7) | 5(55.6) | 19(79.2) |  |
| Yes | 9(27.3) | 4(44.4) | 5(20.8) |  |
| Extrahepatic metastases | |  |  | 0.71 |
| Non | 27(81.8) | 7(77.8) | 20(83.3) |  |
| Yes | 6(18.2) | 2(22.2) | 4(16.7) |  |
| HBsAg |  |  |  | 0.17 |
| 0 | 6(18.2) | 3(33.3) | 3(12.5) |  |
| 1 | 27(81.8) | 6(66.7) | 21(87.5) |  |
| HBV DNA |  |  |  | 0.71 |
| >0 | 6(18.2) | 2(22.2) | 4(16.7) |  |
| 0 | 27(81.8) | 7(77.8) | 20(83.3) |  |
| TB |  |  |  | 0.30 |
| <13 | 12(36.4) | 2(22.2) | 10(41.7) |  |
| >=13 | 21(63.6) | 7(77.8) | 14(58.3) |  |
| ALB |  |  |  | / |
| <50 | 33(100) | 9(100) | 24(100) |  |
| ALT |  |  |  | 0.30 |
| <41 | 21(63.6) | 7(77.8) | 14(58.3) |  |
| >=41 | 12(36.4) | 2(22.2) | 10(41.7) |  |
| AST |  |  |  | 0.48 |
| <38 | 18(54.5) | 4(44.4) | 14(58.3) |  |
| >=38 | 15(45.5) | 5(55.6) | 10(41.7) |  |
| Treatment Strategies | |  |  | 0.72 |
| ICI | 20(60.6) | 5(55.6) | 15(62.5) |  |
| Non-ICI | 13(39.4) | 4(44.4) | 9(37.5) |  |

Table S3

| Patient ID | *FGF3* | *FGF4* | *FGF19* | *CCND1* | Group | BOR | PD as BOR | Primary resistance |
| --- | --- | --- | --- | --- | --- | --- | --- | --- |
| 1 | 0 | 0 | 0 | 0 | nonPD-1Ab | CR | No | No |
| 2 | 0 | 0 | 0 | 0 | nonPD-1Ab | CR | No | No |
| 3 | 0 | 0 | 0 | 0 | nonPD-1Ab | CR | No | No |
| 4 | 0 | 0 | 0 | 0 | nonPD-1Ab | CR | No | No |
| 5 | 0 | 1 | 1 | 1 | nonPD-1Ab | PR | No | No |
| 6 | 0 | 0 | 0 | 0 | nonPD-1Ab | PR | No | No |
| 7 | 0 | 0 | 0 | 0 | nonPD-1Ab | PR | No | No |
| 8 | 0 | 0 | 0 | 0 | nonPD-1Ab | SD | No | No |
| 9 | 1 | 1 | 1 | 1 | nonPD-1Ab | SD | No | Yes |
| 10 | 1 | 1 | 1 | 1 | nonPD-1Ab | SD | No | No |
| 11 | 1 | 0 | 1 | 1 | nonPD-1Ab | SD | No | Yes |
| 12 | 0 | 0 | 0 | 0 | nonPD-1Ab | PD | No | No |
| 13 | 0 | 0 | 0 | 0 | nonPD-1Ab | N/A | Yes | Yes |
| 14 | 0 | 0 | 0 | 0 | PD-1Ab | CR | No | No |
| 15 | 0 | 0 | 0 | 0 | PD-1Ab | CR | No | No |
| 16 | 0 | 0 | 0 | 0 | PD-1Ab | CR | No | No |
| 17 | 0 | 0 | 0 | 0 | PD-1Ab | CR | No | No |
| 18 | 0 | 0 | 0 | 0 | PD-1Ab | PR | No | No |
| 19 | 0 | 0 | 0 | 0 | PD-1Ab | PR | No | No |
| 20 | 0 | 0 | 0 | 0 | PD-1Ab | SD | No | Yes |
| 21 | 0 | 0 | 0 | 0 | PD-1Ab | SD | No | Yes |
| 22 | 0 | 0 | 0 | 0 | PD-1Ab | PD | Yes | Yes |
| 23 | 0 | 0 | 1 | 1 | PD-1Ab | PD | Yes | Yes |
| 24 | 0 | 0 | 0 | 0 | PD-1Ab | SD | No | Yes |
| 25 | 0 | 1 | 1 | 1 | PD-1Ab | PD | Yes | Yes |
| 26 | 1 | 0 | 0 | 1 | PD-1Ab | PD | Yes | Yes |
| 27 | 0 | 0 | 0 | 0 | PD-1Ab | SD | No | No |
| 28 | 0 | 0 | 0 | 0 | PD-1Ab | PD | Yes | Yes |
| 29 | 1 | 1 | 1 | 1 | PD-1Ab | PD | Yes | Yes |
| 30 | 0 | 0 | 0 | 0 | PD-1Ab | PD | Yes | Yes |
| 31 | 1 | 1 | 1 | 1 | PD-1Ab | PD | Yes | Yes |
| 32 | 0 | 0 | 0 | 0 | PD-1Ab | N/A | Yes | Yes |
| 33 | 0 | 0 | 0 | 0 | PD-1Ab | N/A | Yes | Yes |

Annotations: “0” means no amplification in genes (*FGF3, FGF4, FGF19* and *CCND1*). “1” means the amplification in genes (*FGF3, FGF4, FGF19* and *CCND1*). BOR, best overall response. N/A, not available.

Table S4. The association of amplification of individual genes with poor response (PD as BOR) to systemic therapy

|  | | | PD as BOR | | Fisher's exact test |
| --- | --- | --- | --- | --- | --- |
|  |  |  | No | Yes |  |
| PD-1Ab group | FGF3 amplification | No | 10 | 7 | 0.211 |
|  |  | Yes | 0 | 3 |  |
|  | FGF4 amplification | No | 10 | 7 | 0.211 |
|  |  | Yes | 0 | 3 |  |
|  | FGF19 amplification | No | 10 | 6 | 0.087 |
|  |  | Yes | 0 | 4 |  |
|  | CCND1 amplification | No | 10 | 5 | 0.033 |
|  |  | Yes | 0 | 5 |  |
|  | Amp11q13 | No | 10 | 5 | 0.033 |
|  |  | Yes | 0 | 5 |  |
| nonPD-1Ab group | FGF3 amplification | No | 9 | 1 | 1 |
|  |  | Yes | 3 | 0 |  |
|  | FGF4 amplification | No | 9 | 1 | 1 |
|  |  | Yes | 3 | 0 |  |
|  | FGF19 amplification | No | 8 | 1 | 1 |
|  |  | Yes | 4 | 0 |  |
|  | CCND1 amplification | No | 8 | 1 | 1 |
|  |  | Yes | 4 | 0 |  |
|  | Amp11q13 | No | 8 | 1 | 1 |
|  |  | Yes | 4 | 0 |  |

Annotation: BOR, best overall response.
